# Comparative analysis of mitochondrial genomes between a wheat K-type cytoplasmic male sterility (CMS) line and its maintainer line

**DOI:** 10.1186/1471-2164-12-163

**Published:** 2011-03-29

**Authors:** Huitao Liu, Peng Cui, Kehui Zhan, Qiang Lin, Guoyin Zhuo, Xiaoli Guo, Feng Ding, Wenlong Yang, Dongcheng Liu, Songnian Hu, Jun Yu, Aimin Zhang

**Affiliations:** 1 The State Key Laboratory of Plant Cell and Chromosome Engineering, Institute of Genetics and Developmental Biology, Chinese Academy of Sciences, Beijing, 100101, China; 2 The CAS Key Laboratory of Genome Sciences and Information, Beijing Institute of Genomics, Chinese Academy of Sciences, Beijing, 100029, China; 3 Agronomy College of Henan Agricultural University, Zhengzhou, 450002, China; 4 College of Biological Sciences of China Agricultural University, Beijing, 100094, China; 5 Graduate University of Chinese Academy of Sciences, Beijing, 100049, China

## Abstract

**Background:**

Plant mitochondria, semiautonomous organelles that function as manufacturers of cellular ATP, have their own genome that has a slow rate of evolution and rapid rearrangement. Cytoplasmic male sterility (CMS), a common phenotype in higher plants, is closely associated with rearrangements in mitochondrial DNA (mtDNA), and is widely used to produce F1 hybrid seeds in a variety of valuable crop species. Novel chimeric genes deduced from mtDNA rearrangements causing CMS have been identified in several plants, such as rice, sunflower, pepper, and rapeseed, but there are very few reports about mtDNA rearrangements in wheat. In the present work, we describe the mitochondrial genome of a wheat K-type CMS line and compare it with its maintainer line.

**Results:**

The complete mtDNA sequence of a wheat K-type (with cytoplasm of *Aegilops kotschyi*) CMS line, Ks3, was assembled into a master circle (MC) molecule of 647,559 bp and found to harbor 34 known protein-coding genes, three rRNAs (18 S, 26 S, and 5 S rRNAs), and 16 different tRNAs. Compared to our previously published sequence of a K-type maintainer line, Km3, we detected Ks3-specific mtDNA (> 100 bp, 11.38%) and repeats (> 100 bp, 29 units) as well as genes that are unique to each line: *rpl5 *was missing in Ks3 and *trnH *was absent from Km3. We also defined 32 single nucleotide polymorphisms (SNPs) in 13 protein-coding, albeit functionally irrelevant, genes, and predicted 22 unique ORFs in Ks3, representing potential candidates for K-type CMS. All these sequence variations are candidates for involvement in CMS. A comparative analysis of the mtDNA of several angiosperms, including those from Ks3, Km3, rice, maize, *Arabidopsis thaliana*, and rapeseed, showed that non-coding sequences of higher plants had mostly divergent multiple reorganizations during the mtDNA evolution of higher plants.

**Conclusion:**

The complete mitochondrial genome of the wheat K-type CMS line Ks3 is very different from that of its maintainer line Km3, especially in non-coding sequences. Sequence rearrangement has produced novel chimeric ORFs, which may be candidate genes for CMS. Comparative analysis of several angiosperm mtDNAs indicated that non-coding sequences are the most frequently reorganized during mtDNA evolution in higher plants.

## Background

Mitochondria, as semiautonomous organelles, function as manufacturers of cellular ATP through the process of oxidative phosphorylation in all eukaryotes. It is believed that mitochondria originated from a free-living eubacterial ancestor and became an endosymbiotic organelle through engulfment by a eukaryotic host cell [1,2]. The sizes of mitochondrial genomes (mtDNA) vary among eukaryotes, ranging from 6 kb in *Plasmodium *to 200-2000 kb in higher plants [3,4]. Due to frequent mtDNA recombination and extraneous DNA incorporation from the chloroplast (cp) and nuclear genomes, extensive size expansion of mtDNA in higher plants occurs very frequently. In higher plants, in addition to their large genome sizes, mtDNAs display distinctive features, including slow evolutionary rate, rapid rearrangement, frequent insertion, complex multipartite structure, specific mode of gene expression, *cis*-/*trans*-splicing, RNA editing, and use of the universal genetic code [5]. In higher plants, protein-coding genes in mtDNA are extremely conserved but their gene order and non-protein-coding sequences are rather variable [6-8], and their structural organization is very dynamic [9]. The dynamic multipartite structures in higher plants exhibit redundancy and copy number variation [10]. Gene shuffling and variations may result in different phenotypes, such as cytoplasmic male sterility (CMS) [11].

CMS is a common phenotype in higher plants, and is closely associated with mutations in mtDNAs that cause pollen abortion. CMS systems have been widely used as a convenient way to produce F1 hybrid seeds in a variety of valuable crop species, including rice, maize, sugar beet, and cotton. In addition, CMS is exploited to study nucleocytoplasmic interactions [12]. mtDNAs in higher plants are known to have the ability to undergo extensive recombination, resulting in sequence rearrangements. When these rearrangements produce "chimeric genes", they may directly or indirectly alter normal physiological functions, such as pollen abortion. Therefore, comparative analysis of mtDNAs between a CMS line and its normal fertile counterpart should lead to the molecular details underlying the sterility phenotype in higher plants.

Wheat K-type CMS, which lacks adverse cytoplasmic effects and has more restoration line resources than other types of CMS, has been widely used in the production of hybrid seeds. Moreover, we recently sequenced the complete mtDNA genome for fertile Yumai 3 (*Triticum aestivum cv*. Yumai 3, Km3), which is a maintainer line of K-type CMS [13]. In this study, we acquired and analyzed another complete mtDNA from a wheat K-type CMS line, Ks3, with the sterilizing cytoplasm derived from *Aegilops kotschyi*, Boiss.

## Results

### Organization of Ks3 mtDNA

**Figure 1:**
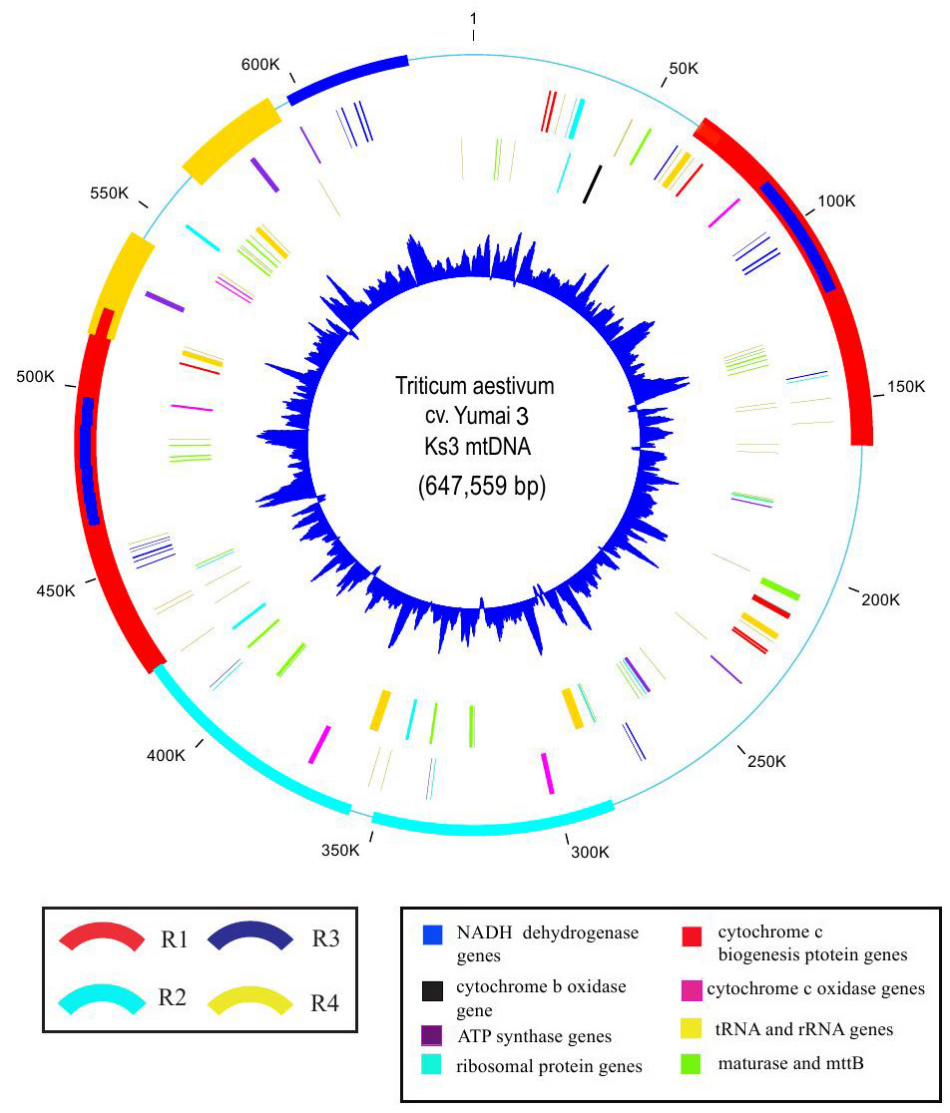
**Physical map of the *Triticum aestivum cv*. Yumai 3 K-type CMS line (Ks3) mitochondrial genome**. Circles display (from outside): **(1) **physical map scaled in kilobase pairs; different colored arcs indicate repeats of more than 20 kb, as in the legend shown in the bottom left corner: red, R1; blue-green, R2; blue, R3; golden yellow, R4; **(2) **and **(3) **coding sequences transcribed clockwise and counterclockwise, respectively: different colors represent the different genes, as in the legend shown in the bottom right corner: blue violet, ATP synthases; blue, NADH dehydrogenases; red, cytochrome c biogenesis proteins; purple-red, cytochrome c oxidases; black, cytochrome b oxidase; blue-green, ribosomal proteins; golden yellow, tRNA and rRNA genes; yellow-green, maturase and mttB; **(4) **GC content variations (in a 1000-bp window and 100-bp increments).

We acquired the Ks3 mtDNA sequence by exploiting a BAC-based cloning strategy, which yielded a circular molecule 647,559 bp in length with 44.3% G+C content (Figure 1). In this master circle (MC) molecule, there were four large repeat sequences of more than 20 kb. The largest was 98,977 bp, extending from 63707 to 162682, including 22 genes (Figure 1 and Additional File 1). The actual Ks3 mtDNA was 400 kb as estimated by removing one copy each of the large repeats with more than 500 bp from the MC molecule. We used similarity searches (BLAST and tRNA scan-SE) and found 53 genes in total; among them, we identified 34 known protein-coding genes, three rRNAs (18 S, 26 S, and 5 S rRNAs), and 16 tRNAs, accounting for 6.22% of the genome (Additional File 1). In addition, using sequence analysis, we classified 248 ORFs longer than 300 bp, which summed to 19.8% or 128,277 bp in total.

We also analyzed the transposable elements in Ks3 mtDNA using TIGR's transposable element database as a reference http://www.tigr.org/tdb/e2k1/plant.repeats/index.shtml with a minimum match of 50 bp. The results showed that there were 12 small fragments, ranging from 59 bp to 230 bp, that were identical to known retrotransposons (Additional File 2). Ten retrotransposons were identical to those of rice and the remaining retrotransposons were identical to those of wheat, with identities ranging from 79% to 98%. The overall length of the retrotransposons was 1476 bp, 0.23% of the total Ks3 mtDNA.

### Ks3-specific mtDNA regions

**Figure 2:**
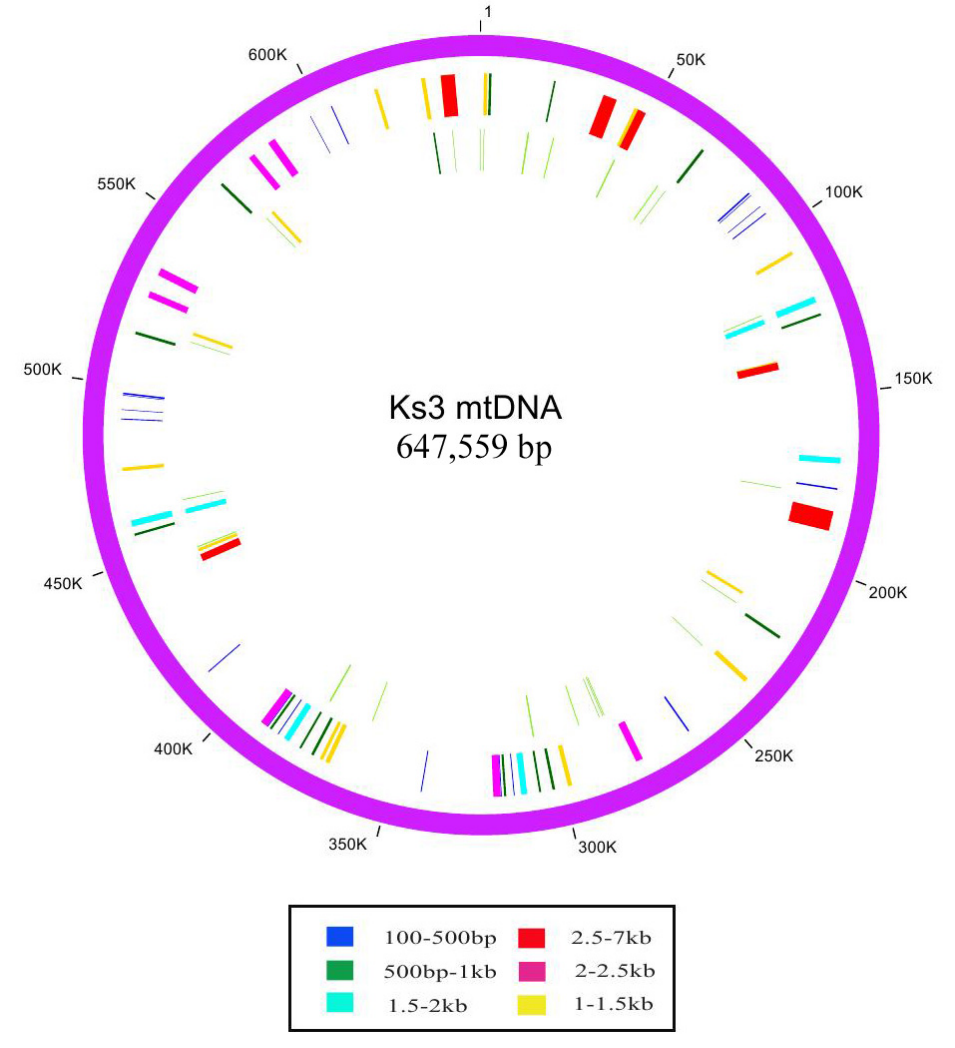
**Distribution of unique sequences and sequences showing homology to wheat ctDNA sequences in Ks3 mtDNA**. Circles display (from outside): **(1) **physical map scaled in kilobase pairs; **(2) **the locations of unique sequences; **(3) **the locations of chloroplast homologous sequences. Different colors represent different sequence lengths: blue, 100-500 bp; dark green, 500-1000 bp; golden yellow, 1-1.5 kb; blue-green, 1.5-2 kb; purple-red, 2-2.5 kb; and red, 2.5-7 kb.

We compared Ks3 mtDNA with that of Km3 using BLAST2, and the analysis revealed 385,765 shared base pair, i.e., 85.2% of the total Km3 sequence. In addition, Ks3 mtDNA had a 574,215-bp sequence that was homologous to Km3 mtDNA, accounting for 88.7% of the total. The conserved sequences in Km3 and Ks3 were broken into 43 and 44 sequence segments, respectively. We also revealed 38 segments (designated U1-U38) of more than 100 bp in Ks3 mtDNA that were not maintained in Km3 mtDNA (Figure 2 Additional Files 3 and 4) and totaled 73,670 bp (11.38%), ranging in size from 120 to 6371 bp and interspersed over 62 locations in the Ks3 MC molecule. It is notable that there were multiple copies in some specific regions. For example, there were four copies of U18, and three copies each of unique regions of U1, U5, and U21. Other unique regions had double or single copies. In the following description, the sum of the length of different specific regions includes every copy unless stated otherwise.

We annotated these 38 Ks3-specific sequences using BlastN and BlastX searching against NCBI databases. Four integrated segments, U17, U18, U19, and U28, were found in the databases with a total of 6727 bp, while 10 segments (10,445 bp) could not be detected, and 24 segments were partially annotated in 37 pieces (19,590 bp). As a result, 26,317 bp were explained, accounting for 35.7% of Ks3-specific sequences and 4% of the Ks3 mitochondrial genome. Furthermore, 21 Ks3-specific segments (20,858 bp), ranging from 33 to 3301 bp, were homologous to several previously determined mitochondrial sequences in higher plants, e.g., *Zea mays*, *Sorghum bicolor*, *Oryza sativa, Bambusa oldhamii*, and *Tripsacum dactyloides*. In addition, partial segments (3991 bp) in U23, U26, and U30 were found to be significantly homologous to wheat chloroplast DNA. Five segments, U8, U11, U14, U24, and U32, partially matched several nuclear genome sequences of different higher plants. Nevertheless, Ks3-specific regions, which accounted for about 47,353 bp and 7.3% of Ks3 mtDNA, were novel to the current NCBI databases.

### Homology of Ks3 mtDNA to wheat ctDNA

We analyzed homology between Ks3 mtDNA and the wheat chloroplast genome (ctDNA) using BLAST2, and revealed 123 segments (25,714 bp, 4%) with more than 81% identity (Additional File 5) and a size range of 24 to 2790 bp. Thirty-eight of these segments were more than 100 bp in length, and summed to 21,040 bp (3.2%) (Figure 2 Additional File 6).

We noticed that some segments in Ks3 mtDNA were homologous to wheat ctDNA with multiple copies; Ct7, Ct11, Ct16, and Ct17 were duplicated and Ct3 has four copies. Fifty-six segments covered the full length or parts of known genes; six segments contained tRNA genes derived from ctDNA (*trnS, trnW, trnC, trnN-1, trnN-2*, and *trnN-3*). The other 50 fragments were classified into 10 mtDNA-derived genes (3792 bp, 0.6%): *atp1, rrn18-1, rrn18-2, rrn18-3, rrn18-4, rrn26-1, rrn26-2, trnM-1*, and *trnM-2*, corresponding to wheat ctDNA genes *atpA, rrn16, rrn23*, and *ct-trnM*.

In addition, by comparing the wheat ctDNA homologies to Ks3 mtDNA with wheat ctDNA homologies to Km3 mtDNA, we observed that most of these homologies with Ks3 and Km3 mtDNA were identical. Only two homologous segments (1930 bp) between wheat ctDNA and Km3 mtDNA were not shared with Ks3 mtDNA. Similarly, four segments (3991 bp) in Ks3 mtDNA were uniquely homologous with wheat ctDNA, and were located in Ks3-specific mtDNA regions, U23, U26, and U30 (Additional File 7). Additional File 6 also indicates that these unique homologous segments of Ks3 mtDNA and wheat ctDNA were contained in Ct18, Ct21, Ct24, and Ct24R. The results reveal that the mitochondrial genomes of Ks3 and Km3 may incorporate some specific extraneous DNA from the wheat chloroplast genome.

### Ks3 mtDNA repeat sequences

**Figure 3:**
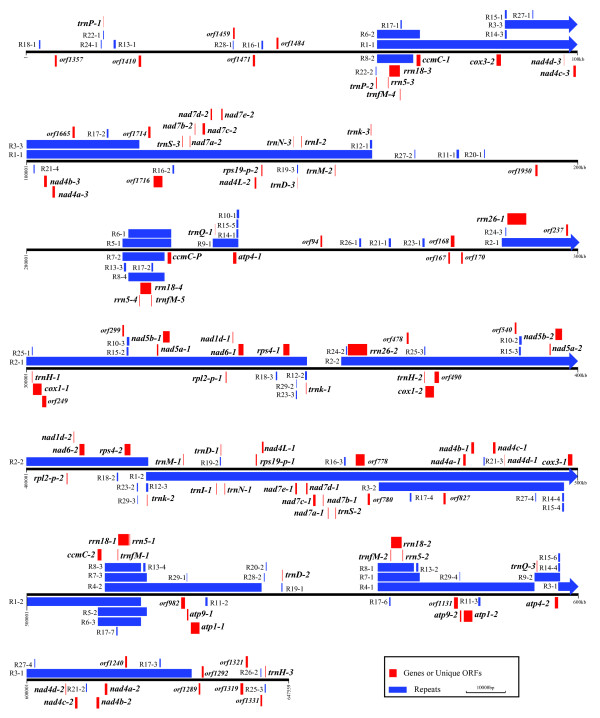
**Distribution of repeats on the physical map of Ks3 mtDNA**. Repeats are indicated by blue bars above or below the horizontal lines; repeats marked with bars above and below the line are direct and inverted, respectively. Genes or Ks3-specific ORFs are indicated by red bars above and below the horizontal lines.

The mtDNAs of higher plants harbor massive repeated sequences. In the Ks3 mtDNA, we defined 29 repeats (> 100 bp), comprising both direct (DR) and inverted repeats (IR) (Table 1); among them, nine involved two copies, twelve had three copies, and six had four copies. There were four large repeats, R1, R2, R3, and R4, which exceeded 20 kb, with lengths of 98,977, 64,991, 33605, and 28,476 bp, respectively. Other repeats were smaller in size and had distinct distributions and copy number variations (Figure 3 Additional File 8).

**Table 1:** Repeats (> 100 bp) found in Ks3 mtDNA.

**No**.	Type^a^	Size (bp)	MC coordinates^b^ Copy-1	Copy-2	Copy-3	Copy-4	Difference between copies	[%IDY]^e^
R1	IR	98977	63707-162682	**520734-421758**			copy-1 1 bp del. ^c^; 3 bp mismatch^d ^	99.99
R2	DR	64991	285968-350958	357152-422142			identical	100
R3	IR/DR	33605	596378-629979	**497569-463965**	86872-120476		copy-1 4 bp del.; 20 bp mismatch	99.93
R4	DR	28476	563639-592113	514143-542618			copy-1 1 bp del.; 3 bp mismatch	99.99
R5	IR	8853	217390-226242	**521779-512927**			2 bp mismatch	99.98
R6	DR/IR	7808	218435-226242	63707-71514	**520734-512927**		3 bp mismatch	99.96
R7	IR/DR	7637	563639-571274	**225026-217390**	514143-521779		copy-1 1 bp del.; 3 bp mismatch	99.97
R8	IR/DR	6592	563639-570299	**70298-63707**	514143-520734	**225026-218435**	copy-1 1 bp del.; 4 bp mismatch	99.92
R9	DR	4645	233766-238410	592108-596752			3 bp mismatch	99.94
R10	DR	442	238110-238550	389430-389871	318246-318687		copy-1 1 bp del.; 7 bp mismatch	98.19
R11	IR	409	178030-178438	**532798-532390**	**582293-581885**		identical	100
R12	IR	385	162298-162682	**350958-350574**	**422142-421758**		identical	100
R13	DR/IR	391	15949-16326	570623-571013	**218041-217651**	521128-521518	copy-1 13 bp del.; 26 bp mismatch	90.03
R14	IR/DR	378	238036-238410	**497569-497192**	86872-87249	596378-596752	copy-1 4 bp del.; 20 bp mismatch	93.4
R15	DR/IR	352	86898-87249	318195-318546	389379-389730	**497543-497192**	copy-1 1 bp del.; copy-5 4 bp del.;	93.1
			238062-238410	596404-596752			20 bp mismatch	
			R15 Copy-5	R15 Copy-6				
R16	IR/DR	295	42766-43060	**126790-126496**	457651-457945		identical	100
R17	DR/IR	275	67932-68206	114585-114859	624088-624362	**469856-469582**	identical	100
			222660-222934	**566004-565730**	**516509-516235**			
			R17 Copy-5	R17 Copy-6	R17 Copy-7			
R18	IR	246	2411-2646	**416690-416445**	**345506-345261**		copy-1 10 bp del.; 5 bp mismatch	94.31
R19	DR/IR	201	546169-546367	435151-435351	**149290-149090**		copy-1 2 bp del.; 2 bp mismatch	98.01
R20	DR	197	182981-183177	543334-543530			1 bp mismatch	99.49
R21	IR/DR	193	265694-265885	**610987-610795**	482957-483149	**101484-101292**	copy-1 1 bp del.; 1 bp mismatch	98.96
R22	IR	190	13994-14183	**63634-63445**			4 bp mismatch	97.89
R23	IR	189	271802-271990	**420288-420100**	**349104-348916**		identical	100
R24	DR	185	13588-13772	358007-358191	286823-287007		identical	100
R25	DR/IR	179	301013-301190	**643373-643195**	372197-372374		copy-1,3 1 bp del.; 9 bp mismatch	94.41
R26	DR	154	260325-260478	642452-642605			4 bp mismatch	97.4
R27	DR/IR	154	91917-92070	170372-170525	**492524-492371**	601420-601573	8 bp mismatch	94.81
R28	DR	145	37571-37703	543007-543151			copy-1 12 bp del.; 2 bp mismatch	90.34
R29	IR/DR	119	529005-529122	**349116-348998**	**420300-420182**	578500-578617	copy-1,4 1 bp del.; 2 bp mismatch	97.48

^a^DR and IR: direct and reverse repeats, respectively; IR/DR: both direct repeat and reverse repeat among multiple copies.

^b ^Boldface: IR copy, compared with copy-1 as control.

^c ^Number of deleted (del.) bases compared to other copies.

^d ^Number of bases without match among the copies.

^e ^IDY: identity.

**Figure 4:**
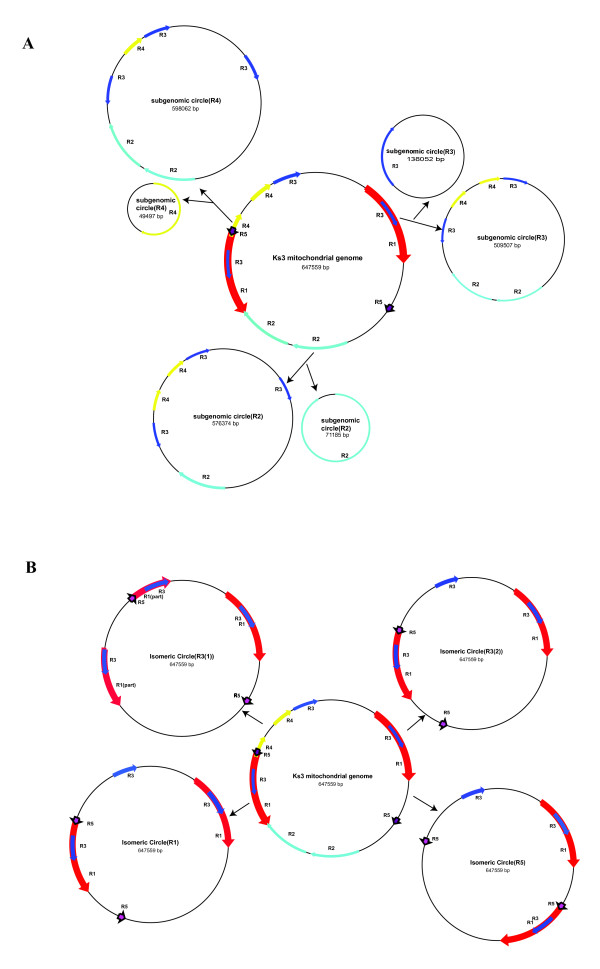
**The predicted multipartite structures of Ks3 mtDNA**. **(A) **Three pairs of subgenomic molecules produced by recombination of the DR pairs: 65 kb (R2), 33 kb (R3), and 28 kb (R4). **(B) **Four isomers of the MC molecule produced by recombination of the IR pairs: 99 kb (R1), 33 kb (R3), 33 kb (R3), and 8.8 kb (R5).

Plant mtDNA is known to contain multipartite structures [14-17]. The isomeric forms of the MC molecule and subgenomic circles are decipherable based on assumptions of intra-molecular homologous recombination [18]. We produced various molecular forms of the Ks3 MC molecule by intra-molecular recombination between different repeat pairs, including three DR of more than 10 kb and four IR of more than 8-kb (Figure 4). Other repeat pairs may also produce possible sites for additional recombination. These subgenomic structures are real. For tobacco mtDNA, subgenomic circles were directly observed using electron microscopy [19], and Sugiyama *et al. *[15] proved that long-range PCR could be used to test recombinant molecules formed by inter-molecule recombination.

Moreover, we also compared repeats between Ks3 mtDNA and Km3 mtDNA. It is known that the Km3 mtDNA sequence is almost identical to the previously reported sequence of *T. aestivum *cv. Chinese Spring, except for seven single nucleotide polymorphisms (SNPs) and 10 indels (insertions and deletions) [13,20]. As a result, Km3 mtDNA and Chinese Spring mtDNA have almost identical repeats (Additional File 9). We found that four repeats (< 500 bp) were almost identical in the two mitochondrial genomes; R12, R19, R20, and R22 in Ks3 mtDNA corresponded to R11, R15, R13, and R14 in Km3 mtDNA. Ten repeats were specific to Ks3 mtDNA and there were six specific repeats in Km3 mtDNA (Additional Files 10, 11). As shown in Additional File 11 the relationship between the large repeats in Km3 and Ks3 mtDNA is complicated. Four large repeats, R1, R2, R3, and R4, in Ks3 mtDNA were much bigger than the corresponding repeats in Km3 mtDNA. R2 and R8 of Km3 showed homology to a fragment located at one end of R2 in Ks3 mtDNA. The two ends of R1 of Km3 were also homologous to the two ends of R2 of Ks3, whereas the central fragment of R1 of Km3 displayed no homology to the repeats of Ks3. A majority of R3 of Km3 was homologous to R4 of Ks3, but it was split in two locations.

### Protein-coding and RNA genes between Ks3 and Km3 mtDNAs

The cytoplasm of Km3 and Ks3 originated from common wheat and *Aegilops kotschyi *belongs to two different genera, *Triticum *and *Aegilops*, respectively. Most of the protein-coding genes are highly conserved, especially in size, except *atp6*, *nad6*, *nad9*, and *rps19-p *(KM is prefixed to the names of genes/ORFs encoded in Km3 and KS is prefixed to those in Ks3; Additional File 12). For instance, the 5'-end of *KSapt6 *and *KMatp6 *is conserved but the 3'-end of *KSapt6 *is extended by 78 bp. Another example is *nad9*: due to deletion of four bases (TGTG) upstream of *KSnad9*, its ORF is 291 bp shorter than that of *KMnad9*. An extreme case is *rpsl5*, which is absent in Ks3 but present in Km3. A DNA exchange between mtDNA and nuclear DNA might have occurred, resulting in a nuclear *rps15 *protein, if the *rps15 *protein is proven to exist; otherwise, we have a defective Ks3 mitochondrial ribosome without *rpl5*.

We identified 32 SNPs scattered among 13 protein-coding genes: 12 were synonymous (*KSapt1, KSmatR, KSrps13*, and *KSnad4*) and 20 were non-synonymous (Table 2). Most of these variations were actually transversions rather than the expected transitions. It is also remarkable that, when compared with Km3, many variations were found among ribosomal protein-coding genes, such as *KSrps1*, *KSrps2*, *KSrps3*, and *KSrps4*. These non-synonymous changes in protein sequences are candidates for functional scrutiny in searching for molecular mechanisms of CMS, since protein-coding genes in plant mtDNA are extraordinarily conservative and their evolutionary rate is very low among different types of plants [6-8].

**Table 2:** Differences in genes coding proteins between the mtDNA of Km3 and Ks3.

Gene	Km3-Ks3 ^a^ (nucleic acid)	Km3-Ks3 ^b^ (amino acid)	Mutation type^c^	SNP type
*atp1*	204T-G		S	transversion
*atp4*	249G-T	84Glu-Pro	N	transversion
	250G-C	84Glu-Pro	N	transversion
	251A-C	84Glu-Pro	N	transversion
*ccmFN*	306C-A		S	transversion
	906A-C		S	transversion
	1103A-T	368Gln-Leu	N	transversion
*cox1*	1575A-G		S	transition
	1456T-A	486Cys-Ser	N	transversion
*cox3*	157A-C	53Ile-Leu	N	transversion
	687G-T	229Gln-His	N	transversion
	688A-C	230Met-Leu	N	transversion
*matR*	1302T-G		S	transversion
*nad3*	185T-C	62Leu-Pro	N	transition
*nad4*	804C-A		S	transversion
	822G-A		S	transition
	1290G-T		S	transversion
	1476C-A		S	transversion
*rps1*	398C-T	133Thr-Ile	N	transition
*rps13*	45A-C		S	transversion
*rps2*	837A-T	280Gln-Lys	N	transversion
	838C-A	280Gln-Lys	N	transversion
	845G-C	282Ser-Thr	N	transversion
	961C-A	321His-Asn	N	transversion
	1002G-T		S	transversion
	1062C-A		S	transversion
*rps3*	256C-A	86Gln-Lys	N	transversion
	505G-T	169Asp-Phe	N	transversion
	505A-T	169Asp-Phe	N	transversion
	1333A-C	445Lys-Gln	N	transversion
*rps4*	146G-T	49Arg-Leu	N	transversion
	236T-G	79Leu-Arg	N	transversion

^a ^Location of base mutation.

^b ^Location of amino acid mutation.

^c ^S, Synonymous; N, Non-synonymous.

A majority of rRNA and tRNA genes were highly conserved between Ks3 and Km3 mtDNAs. Both, however, had missing sequences: Ks3 lost *trnA *and Km3 lost *trnH*. A similar case was also seen among rRNAs. For instance, Ks3 mtDNA did not include *KSrrn26-*p. Moreover, there were more genes and exons in Ks3 than in Km3 mtDNA, as several large-sized repeats were unique to Ks3 mtDNA.

### ORFs between Ks3 and Km3 mtDNAs

**Figure 5:**
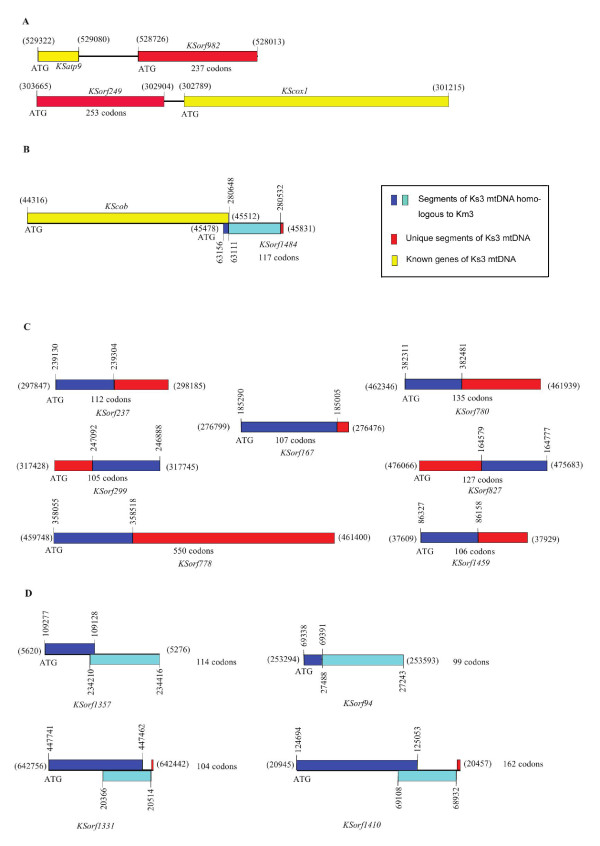
**Unique ORFs of Ks3 mtDNA**. **(A) **ORFs located in the vicinity of known genes; **(B) **ORFs overlapping with known genes; **(C) **partial sequences of ORFs that are homologous to Km3 mtDNA; **(D) **ORFs that are homologous to two discrete segments of Km3 mtDNA. Red bars indicate the unique sequences of Ks3 mtDNA. Blue bars and blue-green bars indicate the homology of Ks3 mtDNA to Km3 mtDNA. Yellow bars indicated known genes of Ks3 mtDNA. The vertical numbers show the coordinates of the homologous fragments of ORFs in the Km3 mtDNA MC molecule. The numbers in parentheses indicate the coordinates of ORFs in the Ks3 mtDNA MC molecule.

Since novel ORFs may be relevant to CMS [21,22], we classified all possible ORFs in the Km3 and Ks3 mtDNA. We found 149 in Km3 and 248 in Ks3 with a length equal to or greater than 300 bp. The additional ORFs in Ks3 reflect the greater length of the Ks3 mtDNA. In addition to copy number and length variations, we also found some ORFs that were unique to Ks3, based on BLAST2 searches (Table 3 Figure 5). Among them, six (*KSorf1289*, *KSorf170*, *KSorf1950*, *KSorf174*, *KSorf168*, and *KSorf982*) were novel; a database search performed with the Blast network service using default parameters revealed no homologies to other sequences in the NCBI databases. Two ORFs (*KSorf1292 *and *KSorf778*), which were situated in two Ks3-specific mtDNA regions (U23 and U30), showed significant homology to wheat chloroplast DNA. As mentioned above, two Ks3 mtDNA fragments homologous to wheat ctDNA were unique and not found in Km3 mtDNA. This indicates that *KSorf1292 *and *KSorf778 *were probably derived from an extraneous wheat chloroplast genome. Another pair of ORFs, *KSorf1321 *and *KSorf1319*, located in a Ks3 mtDNA unique region, U36, exhibited homology to a DNA polymerase in rye mtDNA. This result indicates that *KSorf1321 *and *KSorf1319 *likely encode proteins with similar function in Ks3 mtDNA, but further empirical data are needed. It is notable that *KSorf249 *in Ks3 mtDNA is homologous to *orf256*, a candidate for a sterile gene associated with wheat T-CMS, which originated from the transfer of the wheat nuclear counterpart into *Triticum timopheevii *cytoplasm. In wheat T-CMS, the chimeric gene *orf256 *is situated upstream of *cox1*, is transcribed together with *cox1*, and expresses a 7-kDa protein that is not found in fertile lines [23,24]. Our data showed that *KSorf249 *resembled *orf256 *upstream of *KScox1*, and deserves further study. Previous studies have shown that the ORFs involved in CMS are usually located in the vicinity of known genes or form a chimeric gene by overlapping with parts of known genes in the plant mitochondrial genome. For example, *urf13-T *which leads to CMS in maize T-CMS is located downstream of *atp6*, which provides the regulatory sequence, and the two are co-transcribed [25]. Similarly, *orf107*, the CMS gene in sorghum A3-CMS, forms a chimeric sequence by partially overlapping the 5'-end with that of *atp9 *[26,27]. Some of the Ks3-specific *KSorfs *have similar structures to known ORFs involved in CMS in other plant mtDNAs (Figure 5A, B).

**Table 3:** Unique ORFs of Ks3 mtDNA.

Predicted ORF^a^	Ks3 MC coordinates Copy-1	Copy-2	Copy-3	Size (bp)	Sequence homology	Unique region^b^
*orf94*	253294-253593			300	Two discrete segments of Km3 mtDNA	
orf1289*	631510-631202			309	None	U23
*orf1331*	642756-642442			315	Two discrete segments of Km3 mtDNA	
*orf299(orf540)*	317428-317745	388612-388929		318	Partial homology to Km3 mtDNA	Partial U12
*orf1459*	37609-37929			321	Partial homology to Km3 mtDNA	Partial U37
orf170*	279136-278813			324	None	U32
*orf167*	276799-276476			324	Partial homology to Km3 mtDNA	Partial U32
*orf237(orf478)*	297847-298185	369031-369369		339	Partial homology to Km3 mtDNA	Partial U25
*orf1357*	5620-5276			345	Two discrete segments of Km3 mtDNA	
*orf1484*	45478-45831			354	Two discrete segments of Km3 mtDNA	
orf1292*	631766-632140			375	*Triticum aestivum *chloroplast DNA	U23
orf1321*	639777-640151			375	*Secale cereale *mitochondrial pol-r gene	U36
*orf1240(orf1665*;*orf827)*	617878-618261	108375-108758	476066-475683	384	Partial homology to Km3 mtDNA	Partial U21
orf1950*	192718-192320			399	None	U38
*orf780(orf1714)*	462346-461939	122095-122502		408	Partial homology to Km3 mtDNA	Partial U31
orf1471*	41604-41194			411	None	U37
*orf1410*	20945-20457			489	Two discrete segments of Km3 mtDNA	
orf1319*	639287-638796			492	*Secale cereale *mitochondrial pol-r gene	U36
orf168*	276925-277623			699	None	U32
orf982(orf1131)*	528726-528013	578221-577508		714	None	U31
orf249(orf490)*	303665-302904	374849-374088		762	*orf256*	U17
***orf778(orf1716)***	459748-461400	124693-123041		1653	*Triticum aestivum *chloroplast DNA psaA gene	Partial U30

^a ^Boldface: ORFs that are homologous to wheat ctDNA.

^b ^ORFs are entirely or partially located in the corresponding unique regions of Km3 mtDNA.

* These ORFs do not show significant homology to Km3 mtDNA.

We also categorized Ks3-specific ORFs into two basic groups: those that were partially homologous to Km3 mtDNA (Figure 5C) and those that were almost entirely homologous to Km3 mtDNA (Figure 5D). Partial segments of seven ORFs (*KSorf299*, *KSorf1459*, *KSorf167*, *KSorf237*, *KSorf1240*, *KSorf780*, and *KSorf778*) were located in corresponding Ks3-specific regions, whereas another sequence of these ORFs was homologous to Km3 mtDNA (Table 3). In addition, with the exception of *KSorf167*, these ORFs were located in Ks3 mtDNA repeat regions (Figure 3). As shown in Figure 5B and 5D, five ORFs (*KSorf1357*, *KSorf94*, *KSorf1331*, *KSorf1410*, and *KSorf1484*) had remarkable homology to Km3 mtDNA, but homologous Km3 mtDNA were divided into two discrete segments in the Km3 mitochondrial genome, which indicates that they are likely derived from different parts of Km3 mtDNA. It is notable that five ORFs were not situated in repeat regions of Ks3 mtDNA (Figure 3).

### Comparison among angiosperm mtDNAs

We used MultiPipMaker to align similar regions in two or more DNA sequences using one of the DNA sequences as a reference; in our study, Ks3 mtDNA was used as the reference unless stated otherwise. Comparing Ks3 mtDNA to those of Km3, rice, maize, *Arabidopsis thaliana*, and rapeseed (Additional File 13), we noticed several interesting features. First, the alignable Ks3 sequence (87.6%) was 83% identical to that of Km3 mtDNA. For a more distant sequence comparison, only 34.6% and 32.2% of the Ks3 mtDNA matched those of maize and rice with an identity of more than 78%, respectively. Only 15.6% and 15.5% of the Ks3 mtDNA was shared with *Arabidopsis thaliana *and rapeseed, at more than 76% identity, respectively, and the longest fragment was only 2 kb. Nevertheless, due to greater evolutionary pressure, coding sequences in angiosperm mtDNA are more conservative, whereas the non-coding parts are highly divergent (Additional File 13) [9,28].

We also compared the copy number of mitochondrial genes among Ks3, Km3, maize, and rice (Additional File 14). Ks3 mtDNA appeared to have the most multi-copy genes, in contrast to Km3 mtDNA, in which only *atp6 *and *atp8 *had two copies. Ribosomal protein-coding genes and *trnA *genes appeared to be more divergent among angiosperm mtDNAs. For example, rice and Km3 mtDNAs contained *rpl5 *but maize and Ks3 mtDNAs did not. *KSrpl2*, *KSrps19*, *KMrpl2*, and *KMrps19*, as truncated pseudogenes, were not complete ORFs, whereas *rpl2 *and *rps19 *in rice mtDNA included complete ORFs; however, maize mtDNA did not include these sequences. Moreover, Km3 and maize possessed *trnA *in their mtDNAs, but Ks3 and rice did not.

We also compared gene order in Ks3 mtDNA to that in Km3, maize, and rice mtDNA, excluding tRNA genes (Additional File 15). First, *rrn5 *and *rrn18 *were inserted into the *nad5c-nad1e-matR-rps1-ccmFN *cluster shared by other grass mtDNAs to form a new cluster unique to Ks3 and Km3 mtDNAs. Second, 11 clusters were found to be syntenic in Ks3 and Km3 mtDNAs. Third, Ks3 mtDNA shared four two-gene clusters, *rrn5-rnn18*, *nad3-rps12*, *rps13-nad1bc*, and *nad9-nad2cde*, with rice and maize mtDNAs. Fourth, Ks3 mtDNA shared four two-gene clusters, *rps3a-rpl16*, *rnn26-cox1*, *nad6-rps4 *and *atp1-cox2ab*, with maize alone. Fifth, three other two-gene clusters, *rpl16-rps3b*, *nad4l-rps19*, and *nad5ab-rpl2*, were common only to Ks3 and rice mtDNAs but not to maize mtDNA. These variations in gene order were readily identified by syntenic analysis.

## Discussion

### Comparative analysis of Km3 and Ks3 mtDNAs

The Ks3 MC molecule was 192 kb larger than that of Km3; Ks3 had additional long repeat elements--four of them were more than 20 kb in size--and the longest repeat was 98,977 bp in length. Similar results were also reported in TK18-MS, a cytoplasmic male sterile line of sugar beet, which contains a pair of repeats of 86,816 bp in its MC molecule [29]. Although repeat content of mtDNA can account for more than 30% of total genome sequence length, as in *indica *rice *93-11*, where repeats greater than 2 kb in size constitute 27.7% of the total mtDNA [30], the size of mtDNAs of cytoplasmic male sterile lines seems to be dramatically larger than that of maintainer lines. The intergenic region of plant mtDNAs often contains retrotransposons transferred from nuclear and chloroplast genomes [31,32]. Ks3 mtDNA again had more retrotransposons than Km3: 12 vs. 5. However, the percentages of these retrotransposons in both Ks3 and Km3 were not as high as in maize mtDNA, where retrotransposons account for 4.44% of the total genome [33], but where the rate of gene transfer is generally deemed low [34].

**Figure 6:**
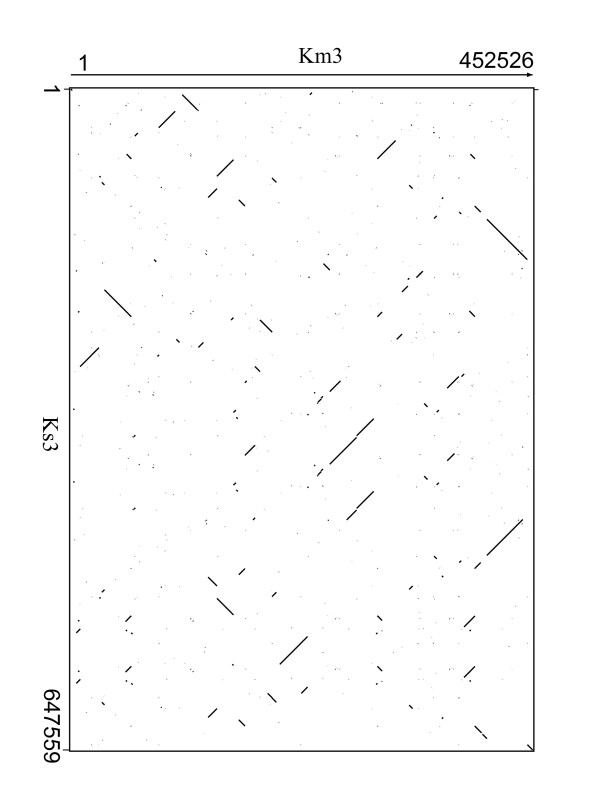
**Dot matrix alignment of the Km3 (x-axis, 1-452526) and Ks3 (y-axis, 1-647559) mtDNAs**.

Frequent recombination events have distorted the synteny between Ks3 and Km3 mtDNAs (Figure 6), as also seen among other plant mtDNAs [35]. Ks3 mtDNA had 11.38% unique sequences when compared to Km3 mtDNA; 7.3% of Ks3 mtDNA sequences are novel but most of these are located in intergenic regions that show a faster rate of evolution [9,28]. Furthermore, although many gene sequences were highly conserved between the two genomes, there were exceptions. *rpl5 *was missing in Ks3, and the sequences of *atp6*, *nad9*, and *nad6 *between Km3 and Ks3 mtDNAs were very different. In addition, the number of SNPs between the Ks3 and Km3 mtDNAs was also significant, compared to those in a CMS line of sugar beet (Owen CMS), which has 24 SNPs in 11 protein-coding genes compared to the fertile form [29]. Finally, there were 22 ORFs unique to Ks3 mtDNA. These differences in protein-coding sequences between Ks3 and Km3 mtDNAs are good candidates for contributing to the CMS phenotype.

### Structural diversity among plant mtDNAs

Our analysis of structural diversity is necessary to understanding the sequence diversity among plant mtDNAs [34]. We detected 29 repeats of more than 100 bp in Ks3 mtDNA, including direct repeats (DR) and inverted repeats (IR), and their roles in shaping subgenomic and isomeric structures in Ks3 mtDNA are of importance. It is believed that proteins encoded by nuclear genes are involved in mismatch repair and recombination of mtDNAs. A gene, *Msh1*, in the nuclear genome homologous to the *Escherichia coli MutS *mismatch repair component, *RecA3*, affects structural diversity in *A. thaliana *[10]. In maize, the P2 nuclear genotype is used as a system for understanding mutations in mtDNA, where abnormal recombination products remarkably increase as the copy number of subgenomic molecules of maize mtDNA increases [36]. Research has shown that when the gene homologous to *Msh1 *in tobacco and tomato is knocked out by RNAi, novel mitochondrial genome organizations are observed, and plants show a male sterility phenotype [37].

### Molecular mechanisms of wheat K-type CMS

We conducted extensive sequence comparison between Ks3 and Km3 mtDNAs to search for functional alterations of genes that were responsible for the CMS phenotype in plants. We noticed that Ks3 mtDNA encodes several partial subunits of the respiratory chain complex, including ATP4, ATP6, NAD3, NAD6, NAD9, COX1, and COX3 (Additional File 16). Any of these altered proteins may interfere with the normal function of respiratory chain reactions, weakening energy supplies and stalling pollen development [38]. In addition, we also observed amino acid variations among RPS1, RPS2, RPS3, RPS4, and ccmFN, as well as a missing RPL5 in Ks3 mtDNA. Whether these variations are related to wheat K-type CMS requires further study.

Research on the expression of novel ORFs in Ks3 mtDNA is also necessary [39], as the relevance of unknown ORFs to CMS has been reported, such as *urf13-T *in maize [40], *orf224 *and *orf222 *in rapeseed [41], *orf522 *in sunflower [42], *orf138 *in radish [43], *orf107 *in sorghum [26], and *orf79 *in rice [21]. The proteins encoded by these ORFs involved in CMS may have structures similar to ATP synthese subunits, which would lead to functional competition; *pcf *in petunia [44] and *orf456 *in pepper [45] were shown to be involved in recombination with the genes encoding cytochrome oxidase (*cox1 *and *cox2*). Another functional scenario is that these novel ORFs may be involved in CMS by damaging mitochondrial membrane structure. In maize, URF13, encoded by *T-urf13*, assembles into a tetramer that penetrates the mitochondrial membrane, and the resulting permeability change affects normal mitochondrial function [46,47].

Previous studies have shown that the process of anther abortion in K-type CMS occurs in the two-cell stage or the late period of the three-cell state of anther development, and the development of pollen is regulated by multiple genes [48]. Therefore, it is necessary to profile the expression of the CMS-specific ORFs in distinct developmental stages, including the microspore mother cell, tetrad, single cell pollen grains, two-cell pollen grains, and three-cell pollen grains. We are preparing to explore molecular mechanisms of wheat K-type CMS through a combination of genomic and proteomic tools, such as the analysis of the transcription and function of the unique ORFs found in Ks3 mtDNA.

## Conclusion

The complete mitochondrial genome of the wheat K-type CMS line Ks3 is very different from that of its maintainer line, Km3, especially in non-coding sequences. The Ks3 mtDNA is 647,559 bp and harbors 34 known protein-coding genes, three rRNAs (18 S, 26 S, and 5 S rRNAs), 16 different tRNAs, Ks3-specific mtDNA (> 100 bp, 11.38%), and repeats (> 100 bp, 29 units). In addition, *rpl5 *is missing, and 32 SNPs are involved in 13 protein-coding, albeit functionally irrelevant, genes, and 22 ORFs are unique in Ks3. All these sequence variations are candidates for CMS. Comparative analysis of the mtDNA of several angiosperms including Ks3, Km3, rice, maize, *Arabidopsis thaliana*, and rapeseed, indicates that non-coding sequences are the most frequently reorganized part of the mitochondrial genome during mtDNA evolution in higher plants.

## Methods

### Plant materials

A wheat CMS line with male-sterile cytoplasm from *Aegilops kotschyi *was designated as K-type Yumai 3 CMS line (abbreviated Ks3), and its isonuclear line with normal male-fertile cytoplasm was designated as K-type Yumai 3 (*Triticum aestivum cv*. Yumai 3) maintainer line (abbreviated Km3) [13]; both were harvested from winter crops in Henan Province, China.

### Mitochondrial DNA extraction

Mitochondria were isolated from etiolated 2-week-old seedlings of Km3 and Ks3 according to a previously published procedure [15]. Mitochondrial fractions were collected by differential centrifugation, incubated with DNase I for 1 h on ice to eliminate linear DNA, and further purified by centrifugation in a discontinuous sucrose-density gradient (1.2 M/1.6 M/2.0 M). The purified mitochondria band was carefully collected from the 1.6 M/1.2 M interface and washed with 0.4 M sucrose. The fraction was finally lysed in 2% Sarkosyl for mtDNA extraction, followed by phenol-chloroform extraction and ethanol precipitation.

### Genome library construction and sequencing

Mitochondrial genome BAC libraries for Ks3 and Km3 were constructed following a previously published procedure with minor modifications [49]. Mitochondria genomic DNA was partially digested with *Sau3AI*, size-fractioned by pulsed-field gel electrophoresis, and ligated to PIndigoBAC-5 *BamHI *cloning-ready vector (Epicentre Biotechnologies, Madison, WI, USA; http://www.epibio.com). The ligation mix was transformed into DH10B-competent cells through electroporation. High-density nylon filters (eight 384-well plates) were screened for a tiling path that covers the entire genome. Shotgun plasmid libraries were made from minimal tiling clones in the pUC-18 vector, and used for sequencing on ABI-3730xl DNA analyzers.

### Analysis of sequence data

The entire nucleotide sequences of Km3 mtDNA (accession number EU534409) and Ks3 mtDNA (accession number GU985444) were determined at the Beijing Institute of Genomics, Chinese Academy of Sciences, and DNA sequences were assembled using the software package phred/phrap/consed [50,51] on a PC/UNIX platform. Physical gaps were closed based on direct sequencing of selected clones. The final assembly of Ks3 mtDNA and Km3 mtDNA included 11,200 and 9931 sequences, respectively. Both genome sequences have nine-fold coverage on average, with a quality value Q20. The final master circle (MC) molecules were obtained with manual editing.

The mitochondrial sequences were annotated with Glimmer 3.0 and BLAST tools, and tRNA genes and their secondary structures were identified according to tRNA scan-SE [52]. The Pairwise BLAST program on our local server was used for comparison between Ks3 mtDNA and Km3 mtDNA and Ks3 mtDNA and the mitochondrial genomes of other plants, with an E-value cutoff at 0.001. A database search was executed using the BLAST network service http://blast.ncbi.nlm.nih.gov/Blast.cgi with default parameters.

Alignments were obtained using MultiPipMaker, a web-based tool for genomic sequence alignments http://bio.cse.psu.edu/pipmaker[53,54]. The annotated Ks3 mtDNA genomic sequence was used as a reference genome and compared with mtDNA sequences from Km3 (*Triticum aestivum *cv. Yumai 3; EU534409), rice (AB076665, AB076666), maize NB (*Zea mays ssp. Mays *cytotype NB; AY506529), Arabidopsis (*Arabidopsis thaliana*; NC001284), and rapeseed (*Brassica napus *L.; AP006444)

## Abbreviations

CMS: cytoplasmic male sterility; mtDNA: mitochondrial DNA; ctDNA: chloroplast DNA; MC: master circle; DR: direct repeats; IR: inverted repeats; ORF: open reading frame; *atp1*, *atp4*, *atp6*, *atp8*, and *atp9*: ATP synthase subunits 1, 4, 6, 8, and 9 genes; *cob*: apocytochrome b gene; *cox1*-*3*: cytochrome c oxidase subunits 1-3 genes; *nad1*-*7*, *9 *and *nad4L*: NADH dehydrogenase subunits 1-7, 9, and 4L genes; *rpl2-p*, *rpl5*, *rpl16*, *rps1-4*, *rps7*, *rps12-13*, and *rps19-p*: ribosomal protein large and small subunit genes; *rrnS *and *rrnL*: small and large subunit ribosomal RNA (rRNA) genes; *trnX*: transfer RNA (tRNA) genes, where X is the one-letter abbreviation of the corresponding amino acid; SNPs: single nucleotide polymorphisms; aa: amino acids; nt: nucleotides; bp: base pairs; kb: kilobase.

## Additional material

Supplementary data are available at BMC Genomics Online.

## Authors' contributions

HL, PC, QL, GZ, WY, and DL carried out the molecular experiments. KZ, XG, SH, JY, and AZ designed and coordinated all experiments. HL, PC, QL, and FD performed the genomic analyses. All authors contributed to the manuscript and then read and approved the final version.

## Supplementary Material

Additional file 1 **Genes in the Ks3 mitochondrial genome**. The file contains the list of size and MC coordinates of genes in the Ks3 mitochondrial genome, including Known protein-coding genes, rRNAs genes and tRNAs genes. Click here for file

Additional file 2 **Transposons in the Ks3 mitochondrial genome**. The file contains the list of size and MC coordinates of transposons in the Ks3 mitochondrial genome. These transposons were identical to known those of rice or wheat with different identity, respectively. Click here for file

Additional file 3 **List of Ks3 mtDNA unique regions (compared with Km3 mtDNA)**. Compared with Km3 mtDNA, Ks3 mtDNA has 38 specific regions. The file contains the list of size and MC coordinates of those Ks3-specific mtDNA regions. Some unique regions in Ks3 mtDNA were homologous to previously determined sequences in NCBI databases, while others could not be detected in NCBI databases. Click here for file

Additional file 4 **Map of homologous fragments in Ks3 mtDNA unique regions**. These fragments homologous to sequences in NCBI databases mapped to Ks3-specific mtDNA regions. Black bars show Ks3 mtDNA unique regions, and fragments of Ks3 mtDNA unique regions homologous to NCBI databases are indicated by broad red bars. The vertical numbers show the coordinates of the homologous fragments in Ks3 mtDNA unique regions. The letters A, B, and C indicate different annotated sequences in NCBI databases, shown in Additional File 3. Click here for file

Additional file 5 **List of Ks3 mtDNA sequences showing homology to ctDNA sequences**. The file contains the list of size and MC coordinates of Ks3 mtDNA sequences showing homology to ctDNA sequences with more than 81% identity and a size range of 24 to 2790 bp. Click here for file

Additional file 6 **List of Ks3 mtDNA sequences of more than 100 bp showing homology to ctDNA sequences**. The file contains the list of Ks3 mtDNA sequences of more than 100 bp showing homology to ctDNA sequences. These Ks3 mtDNA sequences showed homology to the ctDNA sequences bringing the corresponding chloroplast genes. Click here for file

Additional file 7 **List of wheat ctDNA sequences uniquely homologous to Km3 mtDNA and those uniquely homologous to Ks3 mtDNA**. The file contains the list of size and MC coordinates of wheat ctDNA sequences uniquely homologous to Km3 mtDNA and those uniquely homologous to Ks3 mtDNA with different identity. Click here for file

Additional file 8 **Dot matrix representation of the Ks3 MC molecule**. Repeats of more than 100 bp are marked on the map. Red and blue dots and lines represent direct and inverted repeats, respectively. Nine repeats of more than 500 bp, R1-R9, are marked with arrows. Click here for file

Additional file 9 **List of repeats larger than 100 bp found in Km3 mtDNA**. The file contains the list of type, size and MC coordinates of 16 repeats larger than 100 bp in Km3 mtDNA. Click here for file

Additional file 10 **Homology of mtDNA repeats between Ks3 and Km3**. The file contains the list of homology of mtDNA repeats between Ks3 and Km3. Four repeats were almost identical in the Ks3 mtDNA and Km3 mtDNA, while the relationship between the large repeats in two mitochondrial genomes is complicated. Click here for file

Additional file 11 **Comparison of mtDNA repeats between Ks3 and Km3**. (A) Blue bars above or below the horizontal lines indicate one direct or inverted copy of repeats of Ks3 mtDNA, respectively. Netted bars show repeats of Km3 mtDNA homologous to Ks3 mtDNA, and numbers in brackets indicate coordinates of partial homologous fragments in repeats of Km3 mtDNA. Red bars indicate that the partial fragments in repeats of Km3 mtDNA did not show significant homology to repeats of Ks3 mtDNA. (B) The specific mtDNA repeats of Km3 and Ks3 and mtDNA repeats shared by Ks3 and Km3. A pair of identical repeats is indicated by a left-right arrow. Click here for file

Additional file 12 **Alignments of atp6 (A), nad6 (B), nad9 (C), and rps19-p (D) between Ks3 and Km3**. the file contains the alignments of atp6 (**A**), nad6 (**B**), nad9 (**C**), and rps19-p (**D**) between Ks3 and Km3. The identical sequences are highlighted with a dark background. The vertical arrow in (**D**) indicates deletion in Ks3 mtDNA (Ks3_seq) and STOP shows the location of the terminal codon (TAG). Click here for file

Additional file 13 **MultiPipMaker analysis of the mtDNA of several angiosperms**. Ks3 mtDNA was used as the reference genome for comparison with those of Km3, rice, maize, *Arabidopsis thaliana*, and rapeseed. **(A) **Positions of genes or exons of Ks3 mtDNA are indicated with black bars, and their orientations are shown with arrows. The percentage identity is shown at the right side. **(B) **A schematic representation of **(A) **in which only representative genes of Ks3 mtDNA are marked. Red and green bars indicate identity with Km3 mtDNA and other mtDNAs, respectively. Click here for file

Additional file 14 **The number of copies of mitochondrial genes in Ks3, Km3, maize, and rice**. The file contains the list of the number of copies of mitochondrial genes in Ks3, Km3, maize, and rice. The identity of genes between Ks3 mtDNA and Km3 mtDNA was also shown. Click here for file

Additional file 15 **Correlation of gene order between the mitochondrial gene maps of Ks3 and Km3 (A), maize (B), and rice(C)**. The file contains the comparison of gene order in Ks3 mtDNA to that in Km3, maize, and rice mtDNA. The protein-coding and rRNA-coding genes are arranged from top to bottom for Ks3, and from left to right for Km3, rice, and maize, based on their order in the respective gene maps. Genes of Km3, rice, and maize are indicated by code numbers representing the corresponding Ks3 genes on the left margin of figures. Click here for file

Additional file 16 **Alignments of ATP6, NAD6, and NAD9 between Km3 and Ks3**. the file contains the alignments of (**A**) KMATP6 and KSATP6, (**B**) KMNAD6 and KSNAD6, and (**C**) KMNAD9 and KSNAD9. The identical sequences are highlighted with a dark background. Click here for file
