# Supplementary material for: Comparative analysis of mitochondrial genomes between a wheat K-type cytoplasmic male sterility (CMS) line and its maintainer line
Source: BMC Genomics. 2011 Mar 29;12:163. doi: 10.1186/1471-2164-12-163 (PMC3079663; doi:10.1186/1471-2164-12-163)
Supplement: Additional file 1 — Genes in the Ks3 mitochondrial genome. The file contains the list of size and MC coordinates of genes in the Ks3 mitochondrial genome, including Known protein-coding genes, rRNAs genes and tRNAs genes. [file 1471-2164-12-163-S1.DOCX]

**Additional File 1. Genes in the Ks3 mitochondrial genome**

| Gene | Size(bp) | MC coordinates  From to | | | Strand | No. of  amino acids |
| --- | --- | --- | --- | --- | --- | --- |
| Complex V |  |  |  |  |  |  |
| *atp1-1* | 1530 | 529785 | 531314 |  | - | 509 |
| *atp1-2* | 1530 | 579280 | 580809 |  | - | 509 |
| *atp4-1* | 579 | 237460 | 238038 |  | - | 192 |
| *atp4-2* | 579 | 595802 | 596380 |  | - | 192 |
| *atp6* | 1248 | 260544 | 261791 |  | + | 415 |
| *atp8* | 471 | 183989 | 184459 |  | + | 156 |
| *atp9-1* | 243 | 529080 | 529322 |  | - | 80 |
| *atp9-2* | 243 | 578575 | 578817 |  | - | 80 |
| Cytochrome c biogenesis genes |  |  |  |  |  |  |
| *ccmB* | 621 | 226454 | 227074 |  | - | 206 |
| *ccmC-1* | 723 | 70866 | 71588 |  | - | 240 |
| *ccmC-2* | 723 | 512853 | 513575 |  | + | 240 |
| ***ccmC-p^$^*** | 649 | 225594 | 226242 |  | - |  |
| *ccmFCa* | 755 | 23619 | 24373 |  | - |  |
| *ccmFCb* | 559 | 22053 | 22611 |  | - |  |
| ***ccmFC*** | **1314** |  |  |  |  | **438** |
| *ccmFN* | 1770 | 213919 | 215688 |  | - | 589 |
| Complex Ⅲ&Ⅳ genes |  |  |  |  |  |  |
| *cob* | 1197 | 44316 | 45512 |  | + | 398 |
| *cox1-1* | 1575 | 301215 | 302789 |  | - | 524 |
| *cox1-2* | 1575 | 372399 | 373973 |  | - | 524 |
| *cox2a* | 390 | 543217 | 543606 |  | + |  |
| *cox2b* | 393 | 544833 | 545225 |  | + |  |
| ***cox2*** | **783** |  |  |  |  | **260** |
| *cox3-1* | 798 | 498268 | 499065 |  | + | 265 |
| *cox3-2* | 798 | 85376 | 86173 |  | - | 265 |
| Other protein coding genes |  |  |  |  |  |  |
| *matR* | 2037 | 207968 | 210004 |  | - | 678 |
| *mttB* | 816 | 52863 | 53678 |  | - | 271 |
| Complex Ⅰgenes |  |  |  |  |  |  |
| *nad1a* | 386 | 61950 | 62335 |  | - |  |
| *nad1b* | 82 | 263240 | 263321 |  | + |  |
| *nad1c* | 192 | 264744 | 264935 |  | + |  |
| *nad1d-1* | 59 | 337445 | 337503 |  | + |  |
| *nad1d-2* | 59 | 408629 | 408687 |  | + |  |
| *nad1e* | 259 | 207184 | 207442 |  | - |  |
| **nad1** | **978** |  |  |  |  | **325** |
| *nad2a* | 153 | 273838 | 273990 |  | - |  |

**Additional File 1. (continued)**

| Gene | Size(bp) | MC coordinates  From to | | | Strand | No. of  Amino acids | |  |
| --- | --- | --- | --- | --- | --- | --- | --- | --- |
| *nad2b* | 392 | 272632 | 273023 |  | - |  | |  |
| *nad2c* | 161 | 558184 | 558344 |  | + |  | |  |
| *nad2d* | 573 | 560758 | 561330 |  | + |  | |  |
| *nad2e* | 188 | 562739 | 562926 |  | + |  | |  |
| ***nad2*** | **1467** |  |  |  |  | **488** | |  |
| *nad3* | 357 | 181883 | 182239 |  | + | 118 | |  |
| *nad4a-1* | 461 | 479256 | 479716 |  | + |  | |  |
| *nad4a-2* | 461 | 614228 | 614688 |  | - |  | |  |
| *nad4a-3* | 461 | 104725 | 105185 |  | - |  | |  |
| *nad4b-1* | 515 | 480741 | 481255 |  | + |  | |  |
| *nad4b-2* | 515 | 612689 | 613203 |  | - |  | |  |
| *nad4b-3* | 515 | 103186 | 103700 |  | - |  | |  |
| *nad4c-1* | 423 | 484690 | 485112 |  | + |  | |  |
| *nad4c-2* | 423 | 608832 | 609254 |  | - |  | |  |
| *nad4c-3* | 423 | 99329 | 99751 |  | - |  | |  |
| *nad4d-1* | 89 | 486768 | 486856 |  | + |  | |  |
| *nad4d-2* | 89 | 607088 | 607176 |  | - |  | |  |
| *nad4d-3* | 89 | 97585 | 97673 |  | - |  | |  |
| ***nad4*** | **1488** |  |  |  |  | **495** | |  |
| *nad4L-1* | 303 | 442748 | 443050 |  | + | 100 | |  |
| *nad4L-2* | 303 | 141391 | 141693 |  | - | 100 | |  |
| *nad5a-1* | 231 | 323719 | 323949 |  | + |  | |  |
| *nad5a-2* | 231 | 394903 | 395133 |  | + |  | |  |
| *nad5b-1* | 1216 | 324812 | 326027 |  | + |  | |  |
| *nad5b-2* | 1216 | 395996 | 397211 |  | + |  | |  |
| *nad5c* | 21 | 206800 | 206820 |  | - |  | |  |
| *nad5d* | 395 | 7966 | 8360 |  | + |  | |  |
| *nad5e* | 150 | 9294 | 9443 |  | + |  | |  |
| ***nad5*** | **2013** |  |  |  |  | **670** | |  |
| *nad6-1* | 918 | 338493 | 339410 |  | + | 305 | |  |
| *nad6-2* | 918 | 409677 | 410594 |  | + | 305 | |  |
| *nad7a-1* | 143 | 454701 | 454843 |  | - |  | |  |
| *nad7a-2* | 143 | 129598 | 129740 |  | + |  | |  |
| *nad7b-1* | 69 | 453819 | 453887 |  | - |  | |  |
| *nad7b-2* | 69 | 130554 | 130622 |  | + |  | |  |
| *nad7c-1* | 467 | 452040 | 452506 |  | - |  | |  |
| *nad7c-2* | 467 | 131935 | 132401 |  | + |  | |  |
| *nad7d-1* | 244 | 450798 | 451041 |  | - |  | |  |
| *nad7d-2* | 244 | 133400 | 133643 |  | + |  | |  |
| *nad7e-1* | 262 | 448837 | 449098 |  | - | |  | |

**Additional File 1. (continued)**

| Gene | Size(bp) | MC coordinates  From to | | | Strand | No. of  amino acids |
| --- | --- | --- | --- | --- | --- | --- |
| *nad7e-2* | 262 | 135343 | 135604 |  | + |  |
| ***nad7*** | **1185** |  |  |  |  | **394** |
| *nad9* | 573 | 556149 | 556721 |  | + | 190 |
| Ribosomal protein genes |  |  |  |  |  |  |
| *rpl16* | 558 | 33176 | 33733 |  | + | 185 |
| ***rpl2-p-1^$^*** | 169 | 336173 | 336341 |  | - |  |
| ***rpl2-p-2^$^*** | 169 | 407357 | 407525 |  | - |  |
| *rps1* | 525 | 213148 | 213672 |  | - | 174 |
| *rps12* | 378 | 182284 | 182661 |  | + | 125 |
| *rps13* | 351 | 261945 | 262295 |  | + | 116 |
| ***rps19-p-1^$^*** | 207 | 441639 | 441845 |  | + |  |
| ***rps19-p-2^$^*** | 207 | 142596 | 142802 |  | - |  |
| *rps2* | 1083 | 552262 | 553344 |  | - | 360 |
| *rps3a* | 74 | 29900 | 29973 |  | + |  |
| *rps3b* | 1612 | 31716 | 33327 |  | + |  |
| ***rps3*** | **1686** |  |  |  |  | **561** |
| *rps4-1* | 1074 | 346666 | 347739 |  | + | 357 |
| *rps4-2* | 1074 | 417850 | 418923 |  | + | 357 |
| *rps7* | 447 | 281836 | 282282 |  | + | 148 |
| rRNA genes |  |  |  |  |  |  |
| *rrn18-1* | 1955 | 516571 | 518525 |  | + |  |
| *rrn18-2* | 1955 | 566066 | 568020 |  | + |  |
| *rrn18-3* | 1955 | 65916 | 67870 |  | - |  |
| *rrn18-4* | 1955 | 220644 | 222598 |  | - |  |
| *rrn26-1* | 3467 | 287186 | 290652 |  | + |  |
| *rrn26-2* | 3467 | 358370 | 361836 |  | + |  |
| *rrn5-1* | 122 | 518640 | 518761 |  | + |  |
| *rrn5-2* | 122 | 568135 | 568256 |  | + |  |
| *rrn5-3* | 122 | 65680 | 65801 |  | - |  |
| *rrn5-4* | 122 | 220408 | 220529 |  | - |  |
| tRNA genes |  |  |  |  |  |  |
| *trnC** | 71 | 26403 | 26473 |  | - |  |
| *trnD-1* | 74 | 435254 | 435327 |  | + |  |
| *trnD-2* | 74 | 546272 | 546345 |  | + |  |
| *trnD-3* | 74 | 149114 | 149187 |  | - |  |
| *trnE* | 72 | 253715 | 253786 |  | + |  |
| *trnF** | 73 | 281556 | 281628 |  | + |  |
| *trnfM-1* | 74 | 516496 | 516569 |  | + |  |
| *trnfM-2* | 74 | 565991 | 566064 |  | + |  |

**Additional File 1. (continued)**

| Gene | Size(bp) | MC coordinates  From to | | | Strand | No. of  Amino acids |
| --- | --- | --- | --- | --- | --- | --- |
| *trnfM-3* | 74 | 47030 | 47103 |  | - |  |
| *trnfM-4* | 74 | 67872 | 67945 |  | - |  |
| *trnfM-5* | 74 | 222600 | 222673 |  | - |  |
| *trnH-1* | 65 | 301017 | 301081 |  | - |  |
| *trnH-2* | 65 | 372201 | 372265 |  | - |  |
| *trnH-3* | 70 | 643304 | 643373 |  | + |  |
| *trnI-1* | 74 | 434477 | 434550 |  | - |  |
| *trnI-2* | 74 | 149890 | 149963 |  | + |  |
| *trnk-1* | 73 | 350712 | 350784 |  | - |  |
| *trnk-2* | 73 | 421896 | 421968 |  | - |  |
| *trnk-3* | 73 | 162472 | 162544 |  | + |  |
| *trnM-1* | 73 | 428442 | 428514 |  | + |  |
| *trnM-2* | 73 | 155926 | 155998 |  | - |  |
| *trnN-1** | 72 | 435962 | 436033 |  | - |  |
| *trnN-2** | 74 | 46826 | 46899 |  | - |  |
| *trnN-3** | 72 | 148408 | 148479 |  | + |  |
| *trnP-1* | 75 | 14043 | 14117 |  | + |  |
| *trnP-2* | 75 | 63511 | 63585 |  | - |  |
| *trnQ-1* | 72 | 234158 | 234229 |  | + |  |
| *trnQ-2* | 72 | 354438 | 354509 |  | - |  |
| *trnQ-3* | 72 | 592500 | 592571 |  | + |  |
| *trnS-1** | 87 | 281048 | 281134 |  | + |  |
| *trnS-2* | 87 | 456087 | 456173 |  | - |  |
| *trnS-3* | 87 | 128268 | 128354 |  | + |  |
| *trnS-4* | 88 | 181283 | 181370 |  | + |  |
| *trnW** | 74 | 165392 | 165465 |  | + |  |
| *trnY* | 83 | 557465 | 557547 |  | + |  |

* Probable chloroplast origin.

^$^ Truncated pseudogenes.
