# Supplementary material for: Comparative analysis of mitochondrial genomes between a wheat K-type cytoplasmic male sterility (CMS) line and its maintainer line
Source: BMC Genomics. 2011 Mar 29;12:163. doi: 10.1186/1471-2164-12-163 (PMC3079663; doi:10.1186/1471-2164-12-163)
Supplement: Additional file 2 — Transposons in the Ks3 mitochondrial genome. The file contains the list of size and MC coordinates of transposons in the Ks3 mitochondrial genome. These transposons were identical to known those of rice or wheat with different identity, respectively. [file 1471-2164-12-163-S2.DOCX]

**Additional File 2. Transposons in the Ks3 mitochondrial genome**

| Ks3 MC coordinates | Size(bp) | Organism | Identity (%) | Annotation |
| --- | --- | --- | --- | --- |
| 34059-34138 | 80 | Oryza | 86 | rn_304-179 retrotransposon |
| 34270-34406 | 137 | Oryza | 81 | rn_304-179 retrotransposon |
| 175891-175994 | 104 | Oryza | 83 | osr35 retrotransposon |
| 201896-201992 | 97 | Oryza | 80 | rn_44-393 retrotransposon |
| 201927-202011 | 85 | Oryza | 82 | rn_16-305 retrotransposon |
| 202098-202195 | 98 | Oryza | 81 | rn_44-26 retrotransposon |
| 202284-202513 | 230 | Oryza | 79 | rn_16-305 retrotransposon |
| 202540-202684 | 145 | Oryza | 84 | rn_16-305 retrotransposon |
| 285922-286135 | 214 | Triticum | 98 | TREP259 retrotransposon,LTR, athila Sabrina_115G1-1 |
| 333217-333275 | 59 | Oryza | 84 | nt66055-66478 putative retrotransposon, Ty3-gypsy-like |
| 357152-357319 | 168 | Triticum | 98 | TREP259 retrotransposon, LTR, athila Sabrina_115G1-1 |
| 404401-404459 | 59 | Oryza | 84 | nt66055-66478 putative retrotransposon, Ty3-gypsy-like |
|  |  |  |  |  |
