# Supplementary material for: Comparative analysis of mitochondrial genomes between a wheat K-type cytoplasmic male sterility (CMS) line and its maintainer line
Source: BMC Genomics. 2011 Mar 29;12:163. doi: 10.1186/1471-2164-12-163 (PMC3079663; doi:10.1186/1471-2164-12-163)
Supplement: Additional file 3 — List of Ks3 mtDNA unique regions (compared with Km3 mtDNA). Compared with Km3 mtDNA, Ks3 mtDNA has 38 specific regions. The file contains the list of size and MC coordinates of those Ks3-specific mtDNA regions. Some unique regions in Ks3 mtDNA were homologous to previously determined sequences in NCBI databases, while others could not be detected in NCBI databases. [file 1471-2164-12-163-S3.DOCX]

**Additional File 3.** **List of Ks3 mtDNA unique regions (compared with Km3 mtDNA)**

| Unique^a^  regions | Ks3 MC coordinates^b^ | Size  (bp) | Sequences homology |
| --- | --- | --- | --- |
| U1 | 87239-87358(**Ⅰ**); 497083-497202(**Ⅱ**); | 120 | None ^c^ |
|  | 596742-596861(**Ⅲ**) |  |  |
| U2 | 91347-91479(**Ⅰ**); 492962-493094(**Ⅱ**) | 133 | None |
| U3 | 317971-318194(**Ⅰ**); 389155-389378(**Ⅱ**) | 224 | None |
| U4 | 314274-314519(**Ⅰ**);385458-385703(**Ⅱ**) | 246 | None |
| U5 | 93876-94155(**Ⅰ**); 490286-490565(**Ⅱ**); | 280 | None |
|  | 603379-603658**(Ⅲ)** |  |  |
| U6 | 341036-341317(**Ⅰ**);412220-412501(**Ⅱ**) | 282 | None |
| U7 | 260548-261007 | 460 | None |
| U8 | 177310-177773 | 464 | Triticum aestivum chromosome 3B-specific BAC library, contig ctg0528b (1-284) ^d^ |
| U9 | 86421-86885(**Ⅰ**);497600-498064(**Ⅱ**) | 465 | Aegilops columnaris cytochrome c oxidase subunit III (cox3) gene (1-33) |
| U10 | 21271-21785 | 515 | Zea mays genotype CMS-S mitochondrial DNA (206-328) |
| U11 | 306782-307395(**Ⅰ**);377966-378579(**Ⅱ**) | 614 | **A**: Oryza sativa Japonica Group Os01g0605850 (Os01g0605850) mRNA (130-239) |
|  |  |  | **B**: Triticum aestivum cDNA, clone: SET2_I23, cultivar: Chinese Spring (235-292) |
|  |  |  | **C**: Zea mays full-length cDNA clone ZM_BFb0016F12 mRNA (392-506) |
| U12 | 316866-317538(**Ⅰ**); 388050-388722(**Ⅱ**) | 673 | Bambusa oldhamii mitochondrion, complete genome (269-673) |
| U13 | 126791-127516(**Ⅰ**);456925-457650(**Ⅱ**) | 726 | Bambusa oldhamii mitochondrion, complete genome (90-726) |
| U14 | 184675-184992 | 318 | Hordeum vulgare receptor-like kinase (Hv3ARK) gene part (1-69) |
| U15 | 185040-185416 | 377 | None |
| U16 | 2647-3422 | 776 | **A**: Zea mays subsp. mays genotype CMS-S mitochondrial DNA (36-152)(584-743) |
|  |  |  | **B**: Tripsacum dactyloides cultivar Pete mitochondrial DNA(361-469) |
| U17 | 302822-303640(**Ⅰ**);374006-374824(**Ⅱ**) | 819 | Aegilops searsii hypothetical protein 256 (ELF) gene, complete |
|  |  |  | cds, and cytochrome C oxidase I (coxI) gene, partial cds (1-819) |
| U18 | 68334-69201 (**Ⅰ**);223062-223929(**Ⅱ**); | 868 | Bambusa oldhamii mitochondrion, complete genome (1-868) |
|  | 515240-516107(**Ⅲ**);564735-565602(**Ⅳ**) |  |  |
| U19 | 169254-170371 | 1118 | Zea luxurians mitochondrion, complete genome (1-1118) |
| U20 | 299976-300923(**Ⅰ**);371160-372107(**Ⅱ**) | 948 | None |

**Additional File 3. (continued)**

| Unique  region | Ks3 MC coordinates | Size  (bp) | Sequences homology |
| --- | --- | --- | --- |
| U21 | 107469-108559(**Ⅰ**);475882-476972(**Ⅱ**); | 1091 | Sorghum bicolor mitochondrial DNA (16-49) (134-952) |
|  | 616972-618062(**Ⅲ**) |  |  |
| U22 | 1281-2410 | 1130 | Sorghum bicolor mitochondrial DNA (879-1108) |
| U23 | **630879-632138** | **1260** | **Triticum aestivum chloroplast DNA (657-1260)** |
| U24 | 636483-637851 | 1369 | **A**: Triticum aestivum clone BAC 897M20, complete sequence (643-732) |
|  |  |  | **B**: Tripsacum dactyloides cultivar Pete mitochondrion, complete genome (892-980) |
| U25 | 298022-299406(**Ⅰ**);369206-370590(**Ⅱ**) | 1385 | Bambusa oldhamii mitochondrion, complete genome (890-1385) |
| U26 | **46028-47440** | **1413** | **A: Triticum aestivum chloroplast DNA, complete genome (550-872)** |
|  |  |  | **B**: Bambusa oldhamii mitochondrion, complete genome (873-937) (989-1077) |
| U27 | 238545-240022 | 1478 | **A**: Oryza sativa Indica Group mitochondrial DNA (897-1478) (230-444) |
|  |  |  | **B**: Zea mays subsp. mays genotype CMS-S mitochondrial DNA (35-127) (785-892) |
|  |  |  | **C**: Bambusa oldhamii mitochondrion, complete genome (433-680) |
| U28 | 170526-171024 | 499 | **A**: Tripsacum dactyloides cultivar Pete mitochondrion, complete genome (1-32) |
|  |  |  | **B**: Zea luxurians mitochondrion, complete genome (23-499) |
| U29 | 311394-313323(**Ⅰ**);382578-384507(**Ⅱ**) | 1930 | Triticum aestivum cultivar Chinese Yumai mitochondrial DNA (944-989) (1688-1725) |
| U30 | **122266-124229**(**Ⅰ**)**;460212-462175**(**Ⅱ**) | **1964** | **Triticum aestivum chloroplast DNA, complete genome (433-1964)** |
| U31 | 526887-528847(**Ⅰ**);576382-578342(**Ⅱ**) | 1961 | L.perenne mitochondrial atp9 gene (384-995) |
| U32 | 276849-279124 | 2276 | Brachypodium distachyon clone BAC DH085B13, complete sequence (1768-1976) |
| U33 | 319200-321570(**Ⅰ**);390384-392754(**Ⅱ**) | 2371 | None |
| U34 | 534014-536471(**Ⅰ**);583509-585966(**Ⅱ**) | 2458 | **A**: Tripsacum dactyloides cultivar Pete mitochondrial DNA (1926-2138) |
|  |  |  | **B**: Bambusa oldhamii mitochondrion, complete genome (2225-2404) |
| U35 | 47699-50412 | 2714 | Oryza sativa Japonica Group mitochondrial DNA (2499-2714) |
| U36 | 638083-642451 | 4369 | Secale cereale mitochondrial pol-r gene, cultivar Halo (44-3344) |
| U37 | 37860-42279 | 4420 | **A**: Zea perennis mitochondrial DNA (1-615) |
|  |  |  | **B**: Triticum aestivum cultivar Chinese Yumai mitochondrial DNA (1810-1863) (2610-2647) |
| U38 | 186625-192995 | 6371 | Oryza sativa Japonica Group mitochondrial DNA (5149-5220) |

^a^ Boldface: wheat chloroplast DNA showed specific homology to Ks3 mtDNA in these unique regions over Km3.

^b^ The MC coordinates of Ks3 mtDNA unique regions and the serial number (Ⅰ,Ⅱ,Ⅲ and Ⅳ) of different copy of unique region.

^c^ These unique regions didn’t show significant homology to previously determined sequences in NCBI databases.

^d^ The fragments located in Ks3 unique mtDNA regions with coordinates in brackets showed significant homology to previously determined sequences in NCBI databases.
