# Supplementary figures and images for: Comparative analysis of mitochondrial genomes between a wheat K-type cytoplasmic male sterility (CMS) line and its maintainer line
Source: BMC Genomics. 2011 Mar 29;12:163. doi: 10.1186/1471-2164-12-163 (PMC3079663; doi:10.1186/1471-2164-12-163)

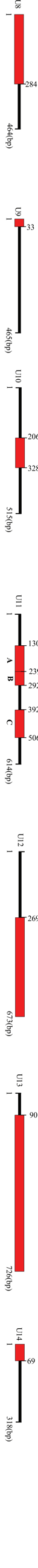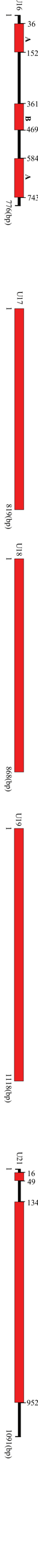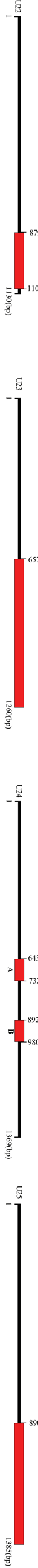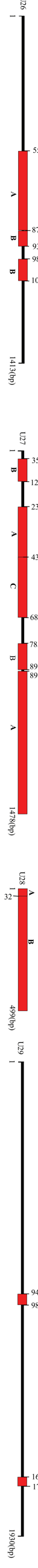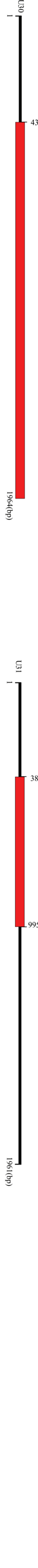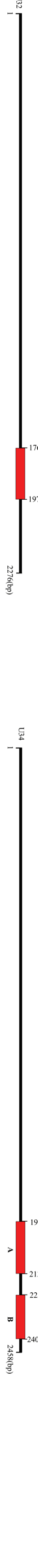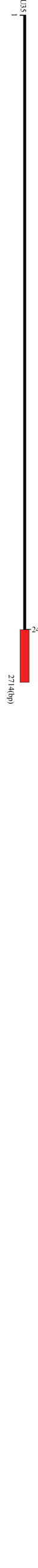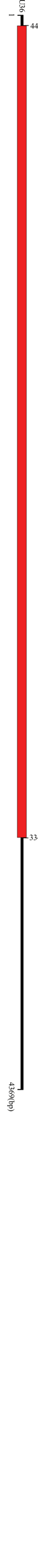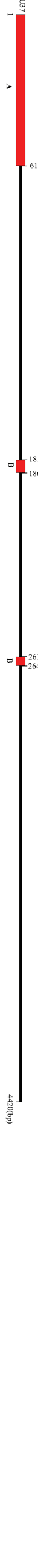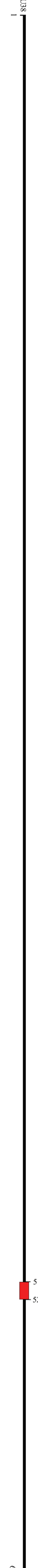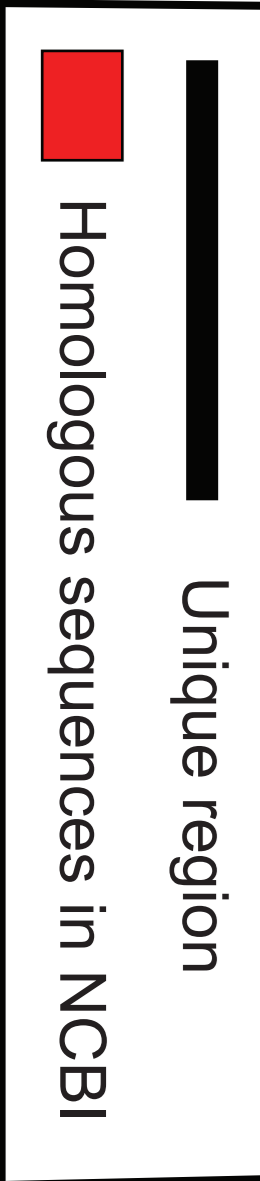

Supplement: Additional file 4 — Map of homologous fragments in Ks3 mtDNA unique regions. These fragments homologous to sequences in NCBI databases mapped to Ks3-specific mtDNA regions. Black bars show Ks3 mtDNA unique regions, and fragments of Ks3 mtDNA unique regions homologous to NCBI databases are indicated by broad red bars. The vertical numbers show the coordinates of the homologous fragments in Ks3 mtDNA unique regions. The letters A, B, and C indicate different annotated sequences in NCBI databases, shown in Additional File 3. [file 1471-2164-12-163-S4.PDF]

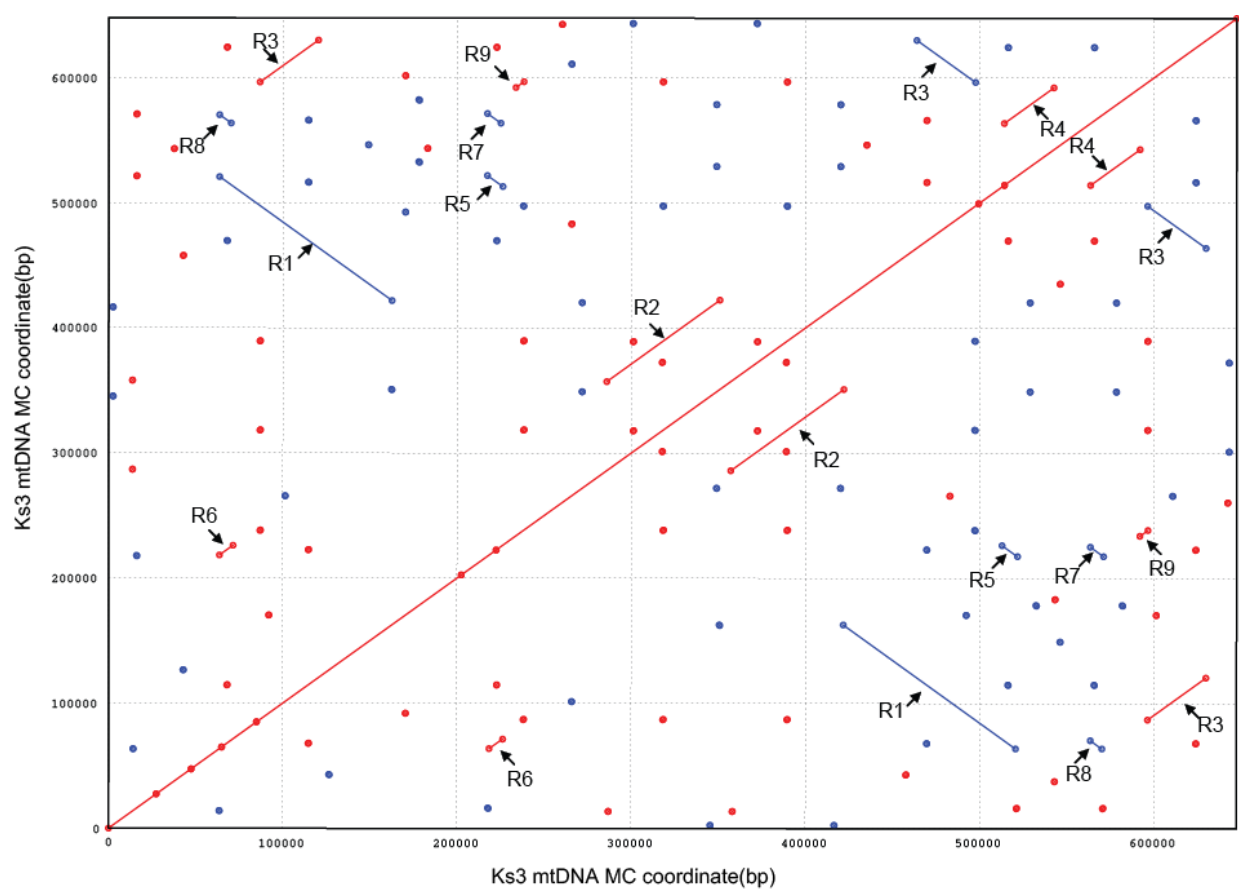

Supplement: Additional file 8 — Dot matrix representation of the Ks3 MC molecule. Repeats of more than 100 bp are marked on the map. Red and blue dots and lines represent direct and inverted repeats, respectively. Nine repeats of more than 500 bp, R1-R9, are marked with arrows. [file 1471-2164-12-163-S8.PDF]

A

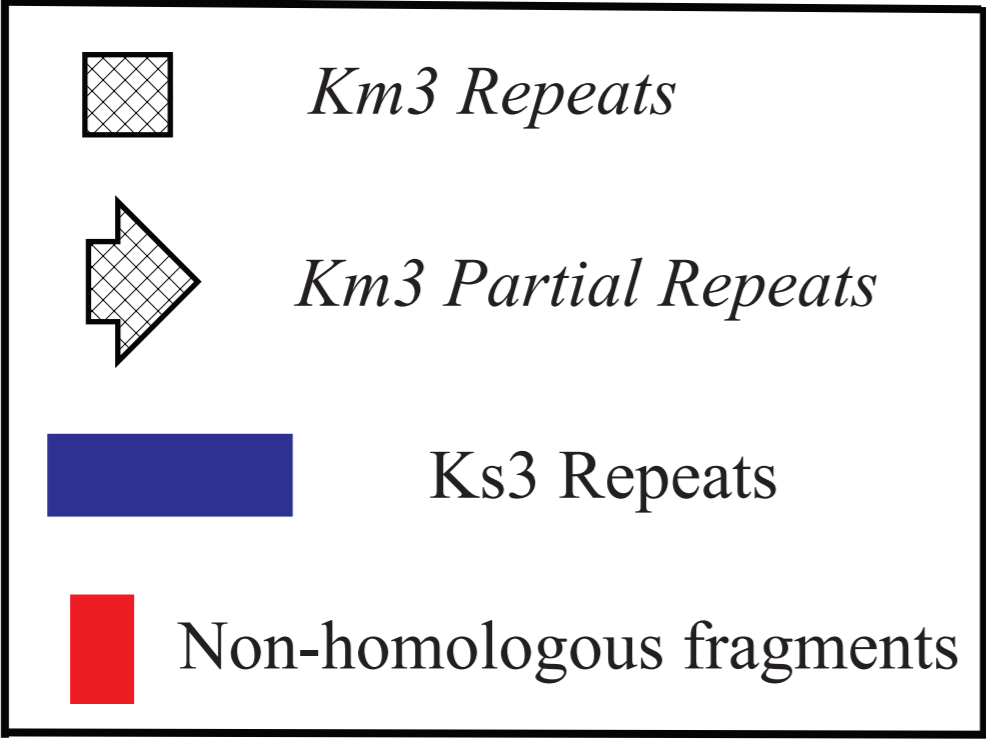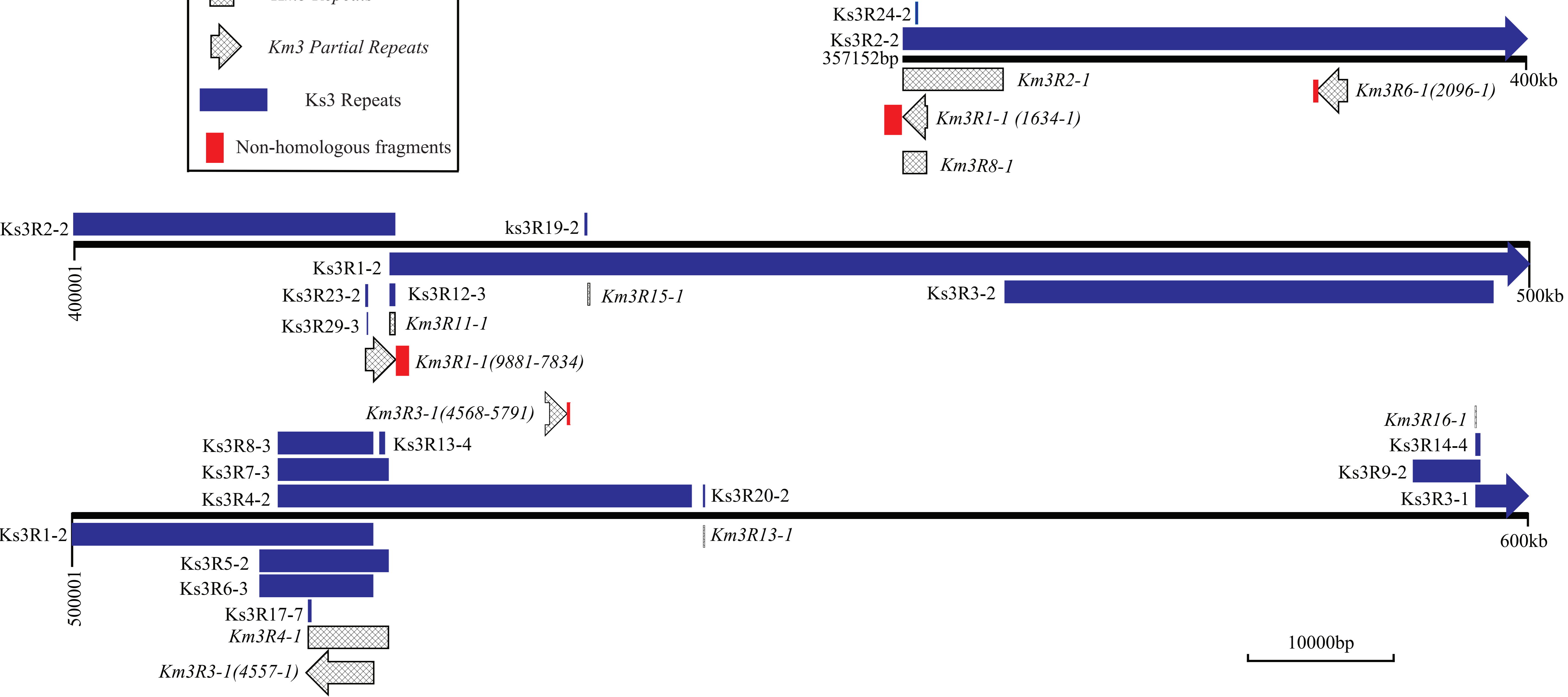

B

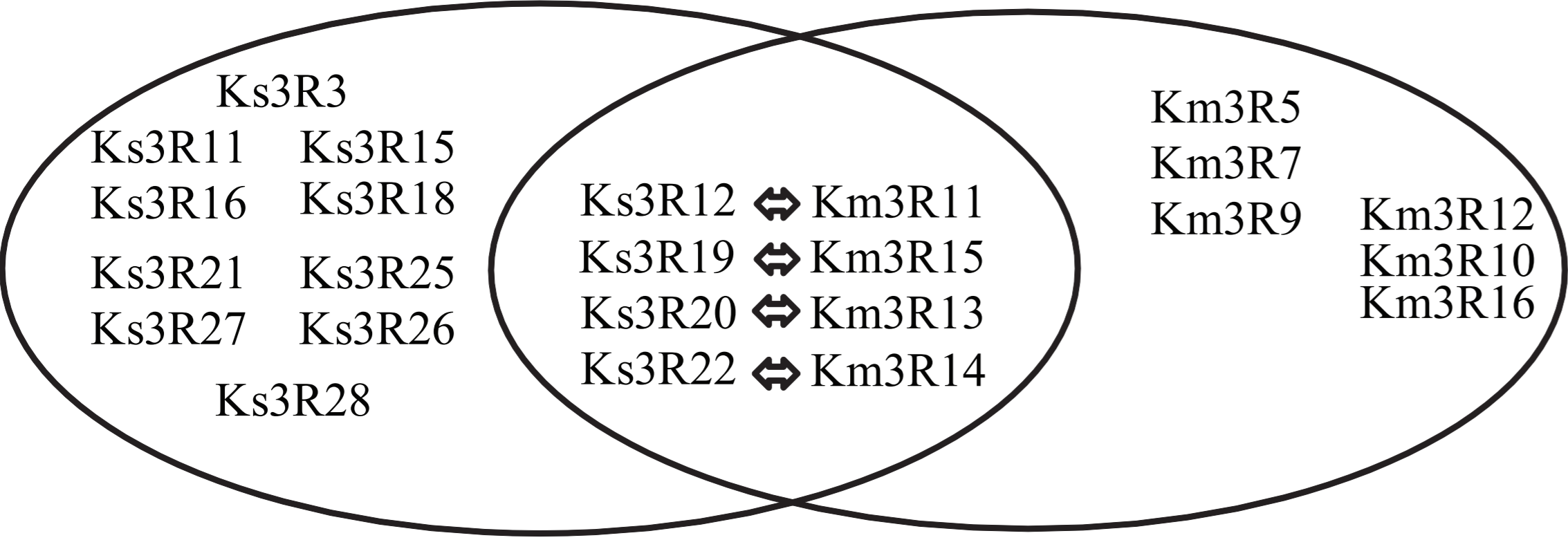

Supplement: Additional file 11 — Comparison of mtDNA repeats between Ks3 and Km3. (A) Blue bars above or below the horizontal lines indicate one direct or inverted copy of repeats of Ks3 mtDNA, respectively. Netted bars show repeats of Km3 mtDNA homologous to Ks3 mtDNA, and numbers in brackets indicate coordinates of partial homologous fragments in repeats of Km3 mtDNA. Red bars indicate that the partial fragments in repeats of Km3 mtDNA did not show significant homology to repeats of Ks3 mtDNA. (B) The specific mtDNA repeats of Km3 and Ks3 and mtDNA repeats shared by Ks3 and Km3. A pair of identical repeats is indicated by a left-right arrow. [file 1471-2164-12-163-S11.PDF]

A

Wheat Ks3

Wheat Km3

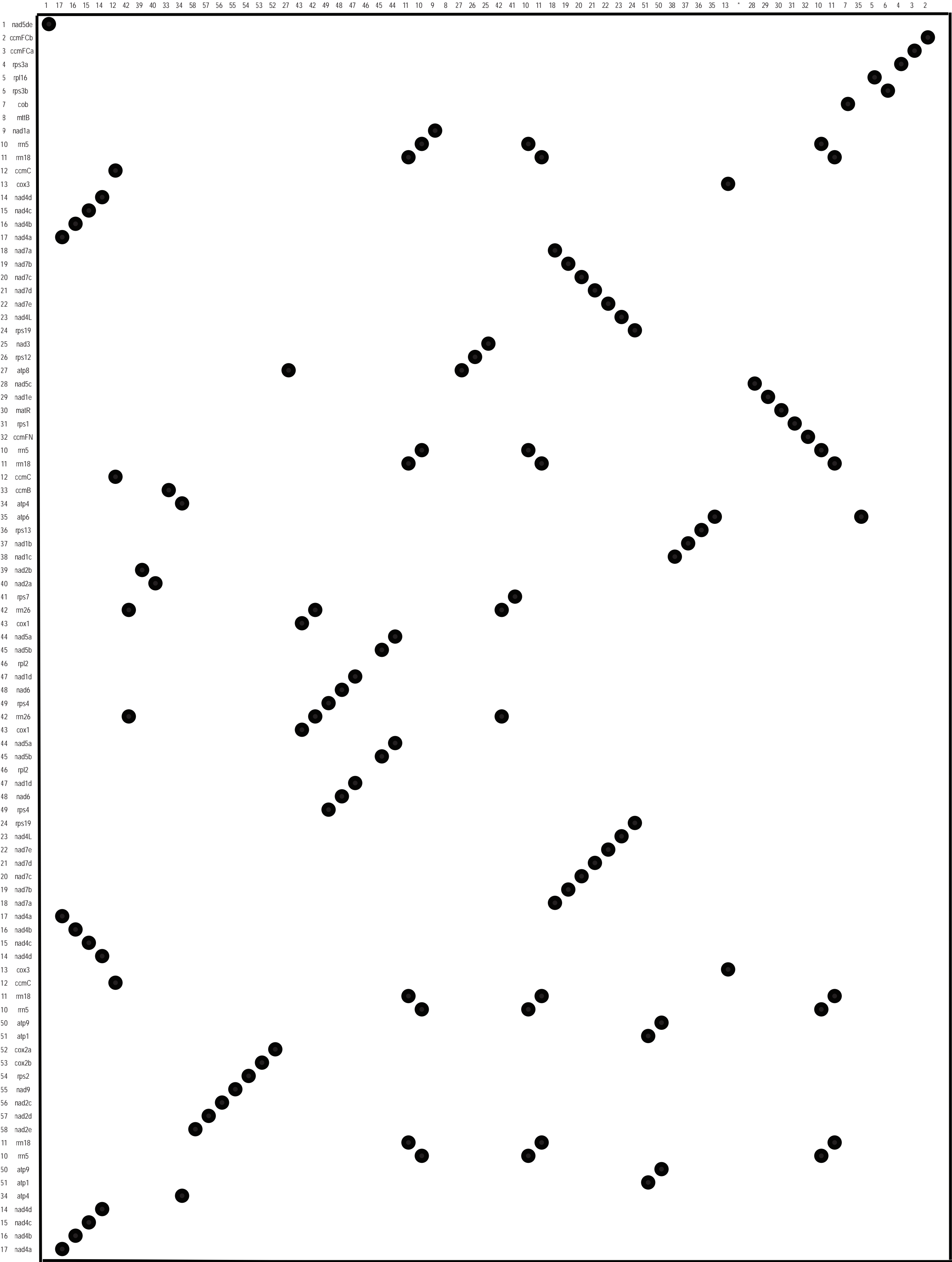

B

Wheat Ks3

Maize

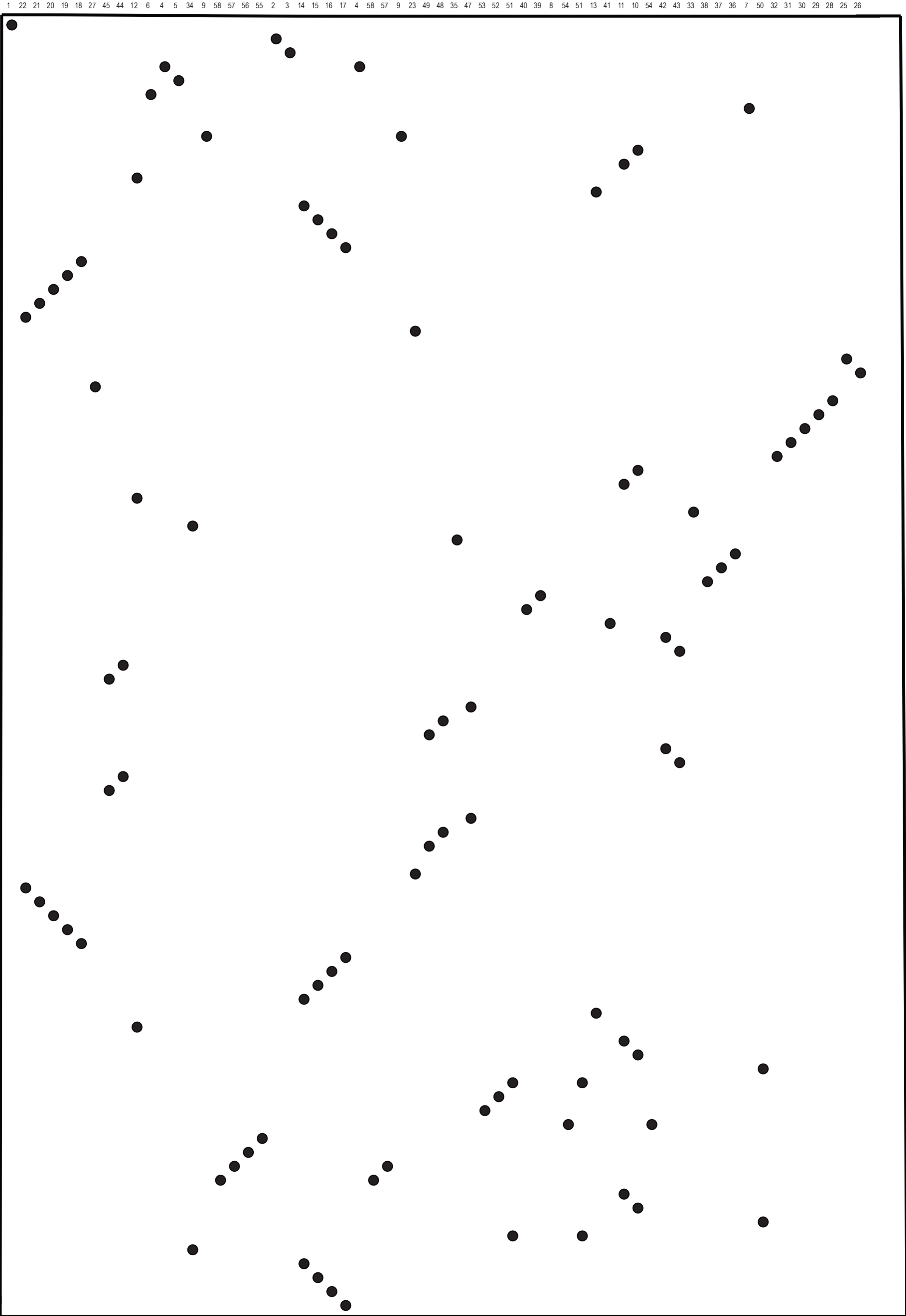

Wheat Ks3

C

Rice

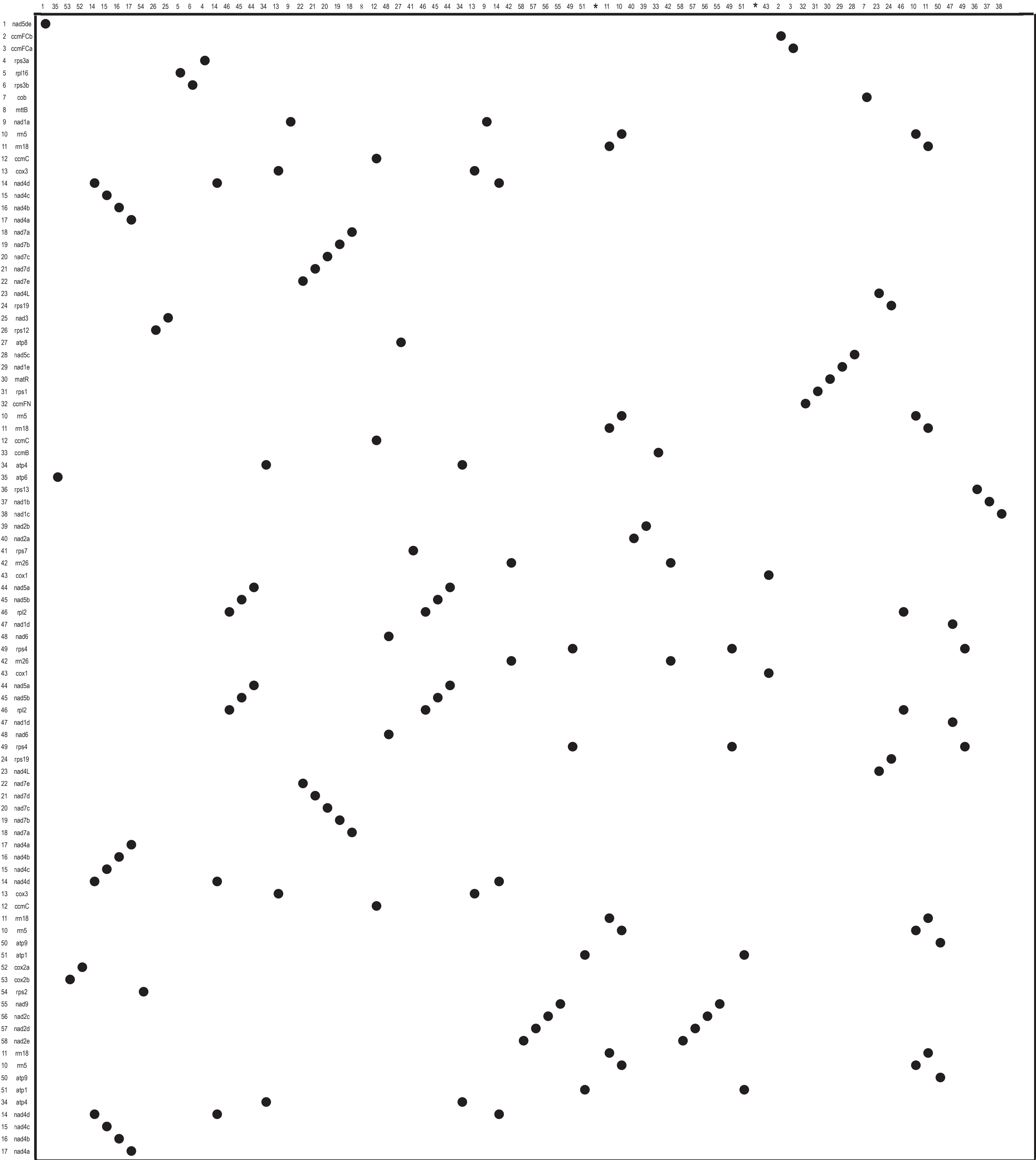

Supplement: Additional file 15 — Correlation of gene order between the mitochondrial gene maps of Ks3 and Km3 (A), maize (B), and rice(C). The file contains the comparison of gene order in Ks3 mtDNA to that in Km3, maize, and rice mtDNA. The protein-coding and rRNA-coding genes are arranged from top to bottom for Ks3, and from left to right for Km3, rice, and maize, based on their order in the respective gene maps. Genes of Km3, rice, and maize are indicated by code numbers representing the corresponding Ks3 genes on the left margin of figures. [file 1471-2164-12-163-S15.PDF]
