# Supplementary material for: Comparative analysis of mitochondrial genomes between a wheat K-type cytoplasmic male sterility (CMS) line and its maintainer line
Source: BMC Genomics. 2011 Mar 29;12:163. doi: 10.1186/1471-2164-12-163 (PMC3079663; doi:10.1186/1471-2164-12-163)
Supplement: Additional file 5 — List of Ks3 mtDNA sequences showing homology to ctDNA sequences. The file contains the list of size and MC coordinates of Ks3 mtDNA sequences showing homology to ctDNA sequences with more than 81% identity and a size range of 24 to 2790 bp. [file 1471-2164-12-163-S5.DOCX]

**Additional File 5. List of Ks3 mtDNA sequences showing homology to ctDNA sequences**

| mtDNA sequence  MC coordinates^a^ | Size(bp) |  | Homologous ctDNA sequence(CopyⅠ)  ctDNA coordinates | Size(bp) | Homologous ctDNA sequence(Copy Ⅱ)  ctDNA coordinates | Size(bp) |  | Nucleotide  sequence  Identity (%) |
| --- | --- | --- | --- | --- | --- | --- | --- | --- |
| 995-1157 | 163 |  | 62057-62218 | 162 |  |  |  | 92 |
| 15883-15935 | 53 |  | 35550-35602 | 53 |  |  |  | 92 |
| 15957-16203 | 247 |  | 35647-35901 | 255 |  |  |  | 90 |
| 16209-16370 | 162 |  | 35916-36072 | 157 |  |  |  | 85 |
| 24554-24633 | 80 |  | 76754-76833 | 80 |  |  |  | 91 |
| 24754-25076 | 323 |  | 77024-77353 | 330 |  |  |  | 82 |
| 26384-24676 | 93 |  | 18662-18754 | 93 |  |  |  | 95 |
| **46577-46899** | 323 |  | 98568-98895 | 328 | 116326-115999 | 328 |  | 92 |
| 47148-47173 | 26 |  | 39956-39981 | 26 |  |  |  | 100 |
| 52599-52636 | 38 |  | 21305-21268 | 38 |  |  |  | 97 |
| 63707-64034 | 328 |  | 35245-34918 | 328 |  |  |  | 96 |
| 64263-64289 | 27 |  | 48098-48072 | 27 |  |  |  | 100 |
| 65942-65974 | 33 |  | 92513-92481 | 33 | 122381-122413 | 33 |  | 96 |
| 66091-66163 | 73 |  | 92410-92337 | 74 | 122484-122557 | 74 |  | 87 |
| 66259-66327 | 69 |  | 92240-92172 | 69 | 122654-122722 | 69 |  | 85 |
| 66712-66786 | 75 |  | 92118-92044 | 75 | 122776-122850 | 75 |  | 85 |
| 66832-66950 | 119 |  | 91997-91879 | 119 | 122897-123015 | 119 |  | 91 |
| 67288-67333 | 46 |  | 91535-91490 | 46 | 123359-123404 | 46 |  | 91 |
| 67417-67502 | 86 |  | 91408-91323 | 86 | 123486-123571 | 86 |  | 91 |

**Additional File 5. (continued)**

| mtDNA sequence  MC coordinates | Size(bp) |  | Homologous ctDNA sequence(CopyⅠ)  ctDNA coordinates | Size(bp) | Homologous ctDNA sequence(Copy Ⅱ)  ctDNA coordinates | Size(bp) |  | Nucleotide  sequence  Identity (%) |
| --- | --- | --- | --- | --- | --- | --- | --- | --- |
| 67795-67854 | 60 |  | 91120-91061 | 60 | 123774-123833 | 60 |  | 88 |
| 70800-70831 | 32 |  | 82945-82976 | 32 | 131949-131918 | 32 |  | 96 |
| 74031-74084 | 54 |  | 83963-84016 | 54 | 130931-130878 | 54 |  | 98 |
| 81506-81568 | 63 |  | 109526-109592 | 67 |  |  |  | 85 |
| 119368-119399 | 32 |  | 49041-49072 | 32 |  |  |  | 96 |
| 120487-120666 | 180 |  | 68077-68256 | 180 |  |  |  | 95 |
| **122698-124675** | 1978 |  | 39110-41098 | 1989 |  |  |  | 97 |
| 137002-137293 | 292 |  | 89045-88744 | 302 | 125849-126150 | 302 |  | 89 |
| 137310-138413 | 1104 |  | 88716-87613 | 1104 | 126178-127281 | 1104 |  | 98 |
| 138456-141240 | 2785 |  | 87569-84780 | 2790 | 127325-130114 | 2790 |  | 98 |
| 141279-141320 | 42 |  | 84752-84711 | 42 | 130142-130183 | 42 |  | 100 |
| 148407-148492 | 86 |  | 98896-98811 | 86 | 115998-116083 | 86 |  | 98 |
| 155925-155997 | 73 |  | 52107-52035 | 73 |  |  |  | 94 |
| 165151-165180 | 30 |  | 64290-64261 | 30 |  |  |  | 96 |
| 165234-165267 | 34 |  | 64084-64051 | 34 |  |  |  | 100 |
| 165388-165469 | 82 |  | 63927-63846 | 82 |  |  |  | 96 |
| 173388-173432 | 45 |  | 51183-51225 | 43 |  |  |  | 91 |
| 179765-179931 | 167 |  | 111403-111572 | 170 |  |  |  | 88 |

|  |  |  |  |  |  |  |  |
| --- | --- | --- | --- | --- | --- | --- | --- |

**Additional File 5. (continued)**

| mtDNA sequence  MC coordinates | | Size(bp) |  | | Homologous ctDNA sequence(CopyⅠ)  ctDNA coordinates | | Size(bp) | | Homologous ctDNA sequence(Copy Ⅱ)  ctDNA coordinates | Size(bp) | |  | | Nucleotide  sequence  Identity (%) |  |
| --- | --- | --- | --- | --- | --- | --- | --- | --- | --- | --- | --- | --- | --- | --- | --- |
| 217647-218762 | 1116 | |  | 36037-34918 | | 1120 | |  | |  |  | | 97 | | |
| 218991-219017 | 27 | |  | 48098-48072 | | 27 | |  | |  |  | | 100 | | |
| 220670-220702 | 33 | |  | 92513-92418 | | 33 | | 122381-122413 | | 33 |  | | 96 | | |
| 220819-220891 | 73 | |  | 92410-92337 | | 74 | | 122484-122557 | | 74 |  | | 87 | | |
| 220987-221055 | 69 | |  | 92240-92172 | | 69 | | 122654-122722 | | 69 |  | | 85 | | |
| 221440-221514 | 75 | |  | 92118-92044 | | 75 | | 122776-122850 | | 75 |  | | 85 | | |
| 221560-221678 | 119 | |  | 91997-91879 | | 119 | | 122897-123015 | | 119 |  | | 91 | | |
| 222016-222061 | 46 | |  | 91535-91490 | | 46 | | 123359-123404 | | 46 |  | | 91 | | |
| 222145-222230 | 86 | |  | 91408-91323 | | 86 | | 123486-123571 | | 86 |  | | 91 | | |
| 222523-222582 | 60 | |  | 91120-91061 | | 60 | | 123774-123833 | | 60 |  | | 88 | | |
| 225528-225559 | 32 | |  | 82945-82976 | | 32 | | 131949-131918 | | 32 |  | | 96 | | |
| 240030-240164 | 135 | |  | 75509-75643 | | 135 | |  | |  |  | | 94 | | |
| 250326-250352 | 27 | |  | 10289-10315 | | 27 | |  | |  |  | | 96 | | |
| 280975-281157 | 183 | |  | 44967-45148 | | 182 | |  | |  |  | | 91 | | |
| 281164-281223 | 60 | |  | 47629-47688 | | 60 | |  | |  |  | | 93 | | |
| 281306-281342 | 37 | |  | 47776-47812 | | 37 | |  | |  |  | | 91 | | |
| 281500-281633 | 134 | |  | 48000-48133 | | 134 | |  | |  |  | | 94 | | |
| 282712-282818 | 107 | |  | 43932-44039 | | 108 | |  | |  |  | | 87 | | |
| 282834-282921 | 88 | |  | 44062-44149 | | 88 | |  | |  |  | | 94 | | |

**Additional File 5. (continued)**

| mtDNA sequence  MC coordinates | Size(bp) |  | Homologous ctDNA sequence(CopyⅠ)  ctDNA coordinates | Size(bp) | Homologous ctDNA sequence(Copy Ⅱ)  ctDNA coordinates | Size(bp) |  | Nucleotide  sequence  Identity (%) |
| --- | --- | --- | --- | --- | --- | --- | --- | --- |
| 287781-287827 | 47 |  | 95276-95322 | 47 | 119618-119572 | 47 |  | 93 |
| 288399-288458 | 60 |  | 95907-95966 | 60 | 118987-118928 | 60 |  | 85 |
| 289490-289534 | 45 |  | 96602-96646 | 45 | 118292-118248 | 45 |  | 95 |
| 289578-289660 | 83 |  | 96683-96765 | 83 | 118211-118129 | 83 |  | 83 |
| 289690-289783 | 94 |  | 96805-96898 | 94 | 118089-117996 | 94 |  | 88 |
| 290150-290397 | 248 |  | 97281-97528 | 248 | 117613-117366 | 248 |  | 81 |
| 300922-300982 | 61 |  | 35290-35230 | 61 |  |  |  | 95 |
| 305871-306095 | 225 |  | 111368-111144 | 225 |  |  |  | 90 |
| 306103-306249 | 147 |  | 111119-110973 | 147 |  |  |  | 90 |
| 309287-309310 | 24 |  | 67935-67912 | 24 |  |  |  | 100 |
| 310874-310905 | 32 |  | 33288-33319 | 32 |  |  |  | 93 |
| 321900-321957 | 58 |  | 34309-34366 | 58 |  |  |  | 96 |
| 358965-359011 | 47 |  | 95276-95322 | 47 | 119618-119572 | 47 |  | 93 |
| 359583-359642 | 60 |  | 95907-95966 | 60 | 118987-118928 | 60 |  | 85 |
| 360674-360718 | 45 |  | 96602-96646 | 45 | 118292-118248 | 45 |  | 95 |
| 360762-360844 | 83 |  | 96683-96765 | 83 | 118211-118129 | 83 |  | 83 |
| 360874-360967 | 94 |  | 96805-96898 | 94 | 118089-117996 | 94 |  | 88 |
| 361334-361581 | 248 |  | 97281-97528 | 248 | 117613-117366 | 248 |  | 81 |
| 372106-372166 | 61 |  | 35290-35230 | 61 |  |  |  | 95 |

**Additional File 5. (continued)**

| mtDNA sequence  MC coordinates | Size(bp) |  | Homologous ctDNA sequence(CopyⅠ)  ctDNA coordinates | Size(bp) | Homologous ctDNA sequence(Copy Ⅱ)  ctDNA coordinates | Size(bp) |  | Nucleotide  sequence  Identity (%) |
| --- | --- | --- | --- | --- | --- | --- | --- | --- |
| 377055-377279 | 225 |  | 111368-111144 | 225 |  |  |  | 90 |
| 377287-377433 | 147 |  | 111119-110973 | 147 |  |  |  | 90 |
| 380471-380494 | 24 |  | 67935-67912 | 24 |  |  |  | 100 |
| 382058-382089 | 32 |  | 33288-333319 | 32 |  |  |  | 93 |
| 393084-393141 | 58 |  | 34309-34366 | 58 |  |  |  | 96 |
| 428443-428515 | 73 |  | 52035-52107 | 73 |  |  |  | 94 |
| 435949-436034 | 86 |  | 98811-98896 | 86 | 116083-115998 | 86 |  | 98 |
| 443121-443162 | 42 |  | 84711-84752 | 42 | 130183-130142 | 42 |  | 100 |
| 443201-445985 | 2785 |  | 84780-87569 | 2790 | 130114-127325 | 2790 |  | 98 |
| 446028-447131 | 1104 |  | 87613-88716 | 1104 | 127281-126178 | 1104 |  | 98 |
| 446806-446869 | 64 |  | 14700-14763 | 64 |  |  |  | 90 |
| 447148-447439 | 292 |  | 88744-89045 | 302 | 126150-125849 | 302 |  | 89 |
| **459766-461743** | 1978 |  | 41098-39110 | 1989 |  |  |  | 97 |
| 463775-463954 | 180 |  | 68256-68077 | 180 |  |  |  | 95 |
| 465042-465073 | 32 |  | 49072-49041 | 32 |  |  |  | 96 |
| 502873-502935 | 63 |  | 109592-109526 | 67 |  |  |  | 85 |
| 510357-510410 | 54 |  | 84016-83963 | 54 | 130878-130931 | 54 |  | 98 |
| 513610-513641 | 32 |  | 82976-82945 | 32 | 131918-131949 | 32 |  | 96 |

|  |  |  |  |  |  |  |  |  |
| --- | --- | --- | --- | --- | --- | --- | --- | --- |

**Additional File 5. (continued)**

| mtDNA sequence  MC coordinates | Size(bp) |  | Homologous ctDNA sequence(CopyⅠ)  ctDNA coordinates | Size(bp) | Homologous ctDNA sequence(Copy Ⅱ)  ctDNA coordinates | Size(bp) |  | Nucleotide  sequence  Identity (%) |
| --- | --- | --- | --- | --- | --- | --- | --- | --- |
| 516587-516646 | 60 |  | 91061-91120 | 60 | 123833-123774 | 60 |  | 88 |
| 516939-517024 | 86 |  | 91323-91408 | 86 | 123571-123486 | 86 |  | 91 |
| 517108-517153 | 46 |  | 91490-91535 | 46 | 123404-123359 | 46 |  | 91 |
| 517491-517609 | 119 |  | 91879-91997 | 119 | 123015-122897 | 119 |  | 91 |
| 517655-517729 | 75 |  | 92044-92118 | 75 | 122850-122776 | 75 |  | 85 |
| 518114-518182 | 69 |  | 92172-92240 | 69 | 122722-122654 | 69 |  | 85 |
| 518278-518350 | 73 |  | 92337-92410 | 74 | 122557-122484 | 74 |  | 87 |
| 518467-518499 | 33 |  | 92481-92513 | 33 | 122413-122381 | 33 |  | 96 |
| 520152-520178 | 27 |  | 48072-48098 | 27 |  |  |  | 100 |
| 520407-521522 | 1116 |  | 34918-36037 | 1120 |  |  |  | 97 |
| 524144-524172 | 29 |  | 34513-34485 | 29 |  |  |  | 100 |
| 530257-530314 | 58 |  | 35696-35639 | 58 |  |  |  | 89 |
| 530373-530438 | 66 |  | 35580-35515 | 66 |  |  |  | 89 |
| 566082-566141 | 60 |  | 91061-91120 | 60 | 123833-123774 | 60 |  | 88 |
| 566434-566519 | 86 |  | 91323-91408 | 86 | 123571-123486 | 86 |  | 91 |
| 566603-566648 | 46 |  | 91490-91535 | 46 | 123404-123359 | 46 |  | 91 |
| 566986-567104 | 119 |  | 91879-91997 | 119 | 123015-122897 | 119 |  | 91 |
| 567150-567224 | 75 |  | 92044-92118 | 75 | 122850-122776 | 75 |  | 85 |

**Additional File 5. (continued)**

| mtDNA sequence  MC coordinates | Size(bp) |  | Homologous ctDNA sequence(CopyⅠ)  ctDNA coordinates | Size(bp) | Homologous ctDNA sequence(Copy Ⅱ)  ctDNA coordinates | Size(bp) |  | Nucleotide  sequence  Identity (%) |
| --- | --- | --- | --- | --- | --- | --- | --- | --- |
| 567609-567677 | 69 |  | 92172-92240 | 69 | 122722-122654 | 69 |  | 85 |
| 567773-567845 | 73 |  | 92337-92410 | 74 | 122557-122484 | 74 |  | 87 |
| 567962-567994 | 33 |  | 92481-92513 | 33 | 122413-122381 | 33 |  | 96 |
| 569647-569673 | 27 |  | 48072-48098 | 27 |  |  |  | 100 |
| 569902-571017 | 1116 |  | 34918-36037 | 1120 |  |  |  | 97 |
| 573639-573667 | 29 |  | 34513-34485 | 29 |  |  |  | 100 |
| 579752-579809 | 58 |  | 35696-35639 | 58 |  |  |  | 89 |
| 579868-579933 | 66 |  | 35580-35515 | 66 |  |  |  | 89 |
| 628871-628902 | 32 |  | 49041-49072 | 32 |  |  |  | 96 |
| **631535-632138** | 604 |  | 90639-90036 | 604 | 124255-124858 | 604 |  | 98 |
| 637852-637983 | 132 |  | 93428-93297 | 132 | 121466-121597 | 132 |  | 94 |
| 647199-647387 | 189 |  | 61844-62032 | 189 |  |  |  | 88 |

^a^ Boldface: the wheat ctDNA showed specific homology to the partial pieces of these segments or entire segments in Ks3 mtDNA over Km3.

Because a 14-bp sequence overlap between two segments of the mtDNA coordinates: 45677-46812 and 46799-46899, total size of 25624 bp is 14-bp smaller than the sum of all the segments (25,638 bp) in the third column in the table.
