# Supplementary material for: Comparative analysis of mitochondrial genomes between a wheat K-type cytoplasmic male sterility (CMS) line and its maintainer line
Source: BMC Genomics. 2011 Mar 29;12:163. doi: 10.1186/1471-2164-12-163 (PMC3079663; doi:10.1186/1471-2164-12-163)
Supplement: Additional file 6 — List of Ks3 mtDNA sequences of more than 100 bp showing homology to ctDNA sequences. The file contains the list of Ks3 mtDNA sequences of more than 100 bp showing homology to ctDNA sequences. These Ks3 mtDNA sequences showed homology to the ctDNA sequences bringing the corresponding chloroplast genes. [file 1471-2164-12-163-S6.DOCX]

**Additional File 6. List of Ks3 mtDNA sequences of more than 100bp showing homology to ctDNA sequences**

| No.^a^ | mtDNA sequence | Size(bp) | mt gene | Homologous ctDNA sequence(CopyⅠ) | Size(bp) | Homologous ctDNA sequence(Copy Ⅱ) | Size(bp) | ct gene | Nucleotide sequence |
| --- | --- | --- | --- | --- | --- | --- | --- | --- | --- |
|  | MC coordinates |  | located | ctDNA coordinates |  | ctDNA coordinates |  | located | Identity (%) |
| Ct1 | 46799-46899 | 101 | *trnN-2(ct)^a^* | 98797-98895 | 99 | 116097-115999 | 99 | *trnN*# | 94 |
| Ct2 | 282712-282818 | 107 | None^b^ | 43932-44039 | 108 |  |  | *ycf3*$ | 87 |
| Ct3-1 | 66832-66950 | 119 | *rrn18-3$* | 91997-91879 | 119 | 122897-123015 | 119 | *rrn16*$ | 91 |
| Ct3-2 | 221560-221678 | 119 | *rrn18-4$* | 91997-91879 | 119 | 122897-123015 | 119 | *rrn16*$ | 91 |
| Ct3R-1 | 517491-517609 | 119 | *rrn18-1$* | 91879-91997 | 119 | 123015-122897 | 119 | *rrn16*$ | 91 |
| Ct3R-2 | 566986-567104 | 119 | *rrn18-2$* | 91879-91997 | 119 | 123015-122897 | 119 | *rrn16*$ | 91 |
| Ct4 | 637852-637983 | 132 | None | 93428-93297 | 132 | 121466-121597 | 132 | *trnI** | 94 |
| Ct5 | 281500-281633 | 134 | *trnF(ct)* | 48000-48133 | 134 |  |  | *trnF*# | 94 |
| Ct6 | 240030-240164 | 135 | None | 75509-75643 | 135 |  |  | *rps11*$ | 94 |
| Ct7-1 | 306103-306249 | 147 | None | 111119-110973 | 147 |  |  | *ndhA*$(copy-1),*ndhA*$(copy-2) | 90 |
| Ct7-2 | 377287-377433 | 147 | None | 111119-110973 | 147 |  |  | *ndhA*$(copy-1),*ndhA*$(copy-2) | 90 |
| Ct8 | 16209-16370 | 162 | None | 35916-36072 | 157 |  |  | *atpA*$ | 85 |
| Ct9 | 995-1157 | 163 | None | 62057-62218 | 162 |  |  | *psbF** | 92 |
| Ct10 | 179765-179931 | 167 | None | 111403-111572 | 170 |  |  | *ndhA*$(copy-1) | 88 |
| Ct11 | 120487-120666 | 180 | None | 68077-68256 | 180 |  |  | *clpP*$ | 95 |
| Ct11R | 463775-463954 | 180 | None | 68256-68077 | 180 |  |  | *clpP*$ | 95 |
| Ct12 | 280975-281157 | 183 | *trnS-1(ct)* | 44967-45148 | 182 |  |  | *trnS*# | 91 |
| Ct13 | 647199-647387 | 189 | None | 61844-62032 | 189 |  |  | *psbF**,*psbE** | 88 |
| Ct14-1 | 305871-306095 | 225 | None | 111368-111144 | 225 |  |  | *ndhA*$(copy-1),*ndhA*$(copy-2) | 90 |
| Ct14-2 | 377055-377279 | 225 | None | 111368-111144 | 225 |  |  | *ndhA*$(copy-1),*ndhA*$(copy-2) | 90 |
|  |  |  |  |  |  |  |  |  |  |

**Additional File 6. (continued)**

| No. | mtDNA sequence  MC coordinates | Size(bp) | mt gene  located | Homologous ctDNA sequence(CopyⅠ)  ctDNA coordinates | Size(bp) | Homologous ctDNA sequence(Copy Ⅱ)  ctDNA coordinates | Size(bp) | ct gene  located | Nucleotide sequence  Identity (%) |
| --- | --- | --- | --- | --- | --- | --- | --- | --- | --- |
| Ct15 | 15957-16203 | 247 | None | 35647-35901 | 255 |  |  | *atpA*$ | 90 |
| Ct16-1 | 290150-290397 | 248 | *rrn26-1*$ | 97281-97528 | 248 | 117613-117366 | 248 | *rrn23*$ | 81 |
| Ct16-2 | 361334-361581 | 248 | *rrn26-2*$ | 97281-97528 | 248 | 117613-117366 | 248 | *rrn23*$ | 81 |
| Ct17 | 137002-137293 | 292 | None | 89045-88744 | 302 | 125849-126150 | 302 | None | 89 |
| Ct17R | 447148-447439 | 292 | None | 88744-89045 | 302 | 126150-125849 | 302 | None | 89 |
| **Ct18** | 46577-46899 | 323 | None | 98568-98895 | 328 | 116326-115999 | 328 | None | 92 |
| Ct19 | 24754-25076 | 323 | None | 77024-77353 | 330 |  |  | *rpl14** | 82 |
| Ct20 | 63707-64034 | 328 | None | 35245-34918 | 328 |  |  | *atpA*$ | 96 |
| **Ct21** | 631535-632138 | 604 | None | 90639-90036 | 604 | 124255-124858 | 604 | None | 98 |
| Ct22 | 137310-138413 | 1104 | None | 88716-87613 | 1104 | 126178-127281 | 1104 | *rps7*# | 98 |
| Ct22R | 446028-447131 | 1104 | None | 87613-88716 | 1104 | 127281-126178 | 1104 | *rps7*# | 98 |
| Ct23 | 217647-218762 | 1116 | None | 36037-34918 | 1120 |  |  | *atpA** | 97 |
| Ct23R-1 | 520407-521522 | 1116 | None | 34918-36037 | 1120 |  |  | *atpA** | 97 |
| Ct23R-2 | 569902-571017 | 1116 | None | 34918-36037 | 1120 |  |  | *atpA** | 97 |
| **Ct24** | 122698-124675 | 1978 | None | 39110-41098 | 1989 |  |  | *psaB*$,*psaA** | 97 |
| **Ct24R** | 459766-461743 | 1978 | None | 41098-39110 | 1989 |  |  | *psaB*$ *psaA** | 97 |
| Ct25 | 138456-141240 | 2785 | None | 87569-84780 | 2790 | 127325-130114 | 2790 | *ndhB*# | 98 |
| Ct25R | 443201-445985 | 2785 | None | 84780-87569 | 2790 | 130114-127325 | 2790 | *ndhB*# | 98 |

^a^ Boldface: the wheat ctDNA showed specific homology to the partial pieces of these segments or entire segments in Ks3 mtDNA over Km3.

$: A small portion located in the respective DNA sequences.

*: larger portion is located in the respective DNA sequences.

#: Complete or nearly complete gene sequence included in the respective DNA sequences.
