# Supplementary material for: Comparative analysis of mitochondrial genomes between a wheat K-type cytoplasmic male sterility (CMS) line and its maintainer line
Source: BMC Genomics. 2011 Mar 29;12:163. doi: 10.1186/1471-2164-12-163 (PMC3079663; doi:10.1186/1471-2164-12-163)
Supplement: Additional file 7 — List of wheat ctDNA sequences uniquely homologous to Km3 mtDNA and those uniquely homologous to Ks3 mtDNA. The file contains the list of size and MC coordinates of wheat ctDNA sequences uniquely homologous to Km3 mtDNA and those uniquely homologous to Ks3 mtDNA with different identity. [file 1471-2164-12-163-S7.DOCX]

**Additional File 7. List of wheat ctDNA sequences uniquely homologous to Km3 mtDNA and those uniquely homologous to Ks3 mtDNA**

| mtDNA | mtDNA sequence | Size(bp) | Homologous ctDNA | Homologous ctDNA | Size(bp) |  | Nucleotide | Unique region |
| --- | --- | --- | --- | --- | --- | --- | --- | --- |
|  | MC coordinates |  | sequence(CopyⅠ) | sequence(CopyⅡ) |  |  | sequence | in Ks3 mtDNA |
|  |  |  | ctDNA coordinates | ctDNA coordinates |  |  | Identity (%) |  |
| Km3 | 75999-74171 | 1829 | 119839-121668 | 95055-93226 | 1830 |  | 98 | - |
| Km3 | 79405-79305 | 101 | 63336-63437 |  | 102 |  | 96 | - |
| Ks3 | 46577-46899 | 323 | 98568-98895 | 116326-115999 | 328 |  | 92 | U26 |
| Ks3 | 631535-632138 | 604 | 124255-124858 | 90639-90036 | 604 |  | 98 | U23 |
| Ks3 | 461743-460212 | 1532 | 39110-40652 |  | 1543 |  | 99 | U30-Ⅱ |
| Ks3 | 122698-124229 | 1532 | 39110-40652 |  | 1543 |  | 99 | U30-Ⅰ |
|  |  |  |  |  |  |  |  |  |
