# Supplementary material for: Comparative analysis of mitochondrial genomes between a wheat K-type cytoplasmic male sterility (CMS) line and its maintainer line
Source: BMC Genomics. 2011 Mar 29;12:163. doi: 10.1186/1471-2164-12-163 (PMC3079663; doi:10.1186/1471-2164-12-163)
Supplement: Additional file 9 — List of repeats larger than 100 bp found in Km3 mtDNA. The file contains the list of type, size and MC coordinates of 16 repeats larger than 100 bp in Km3 mtDNA. [file 1471-2164-12-163-S9.DOCX]

| No. | Type^a^ | Size (bp) | MC coordinates^b^  Copy-1 | Copy-2 | Copy-3 |
| --- | --- | --- | --- | --- | --- |
| Km3R1 | DR | 9881 | 170630-180510 | 262526-272406 |  |
| Km3R2 | DR | 7035 | 257125-264159 | 368863-375897 |  |
| Km3R3 | IR | 6064 | 54623-60686 | **304970-298907** |  |
| Km3R4 | DR | 5469 | 53584-59052 | 390549-396017 |  |
| Km3R5 | IR | 4430 | **304970-300541** | 391588-396017 |  |
| Km3R6 | DR | 2460 | 159587-162046 | 358521-360980 |  |
| Km3R7 | IR | 2045 | 32869-34913 | **326586-324542** |  |
| Km3R8 | DR | 1634 | 170630-172263 | 262526-264159 | 374264-375897 |
| Km3R9 | DR | 1341 | 19042-20382 | 84918-86257 |  |
| Km3R10 | DR | 493 | 233393-233885 | 338884-339376 |  |
| Km3R11 | DR | 385 | 178463-178847 | 270359-270743 | 442526-442910 |
| Km3R12 | DR | 207 | 63737-63943 | 233151-233357 |  |
| Km3R13 | DR | 193 | 224889-225081 | 340162-340354 |  |
| Km3R14 | DR | 190 | 117643-117832 | 305043-305232 |  |
| Km3R15 | DR | 186 | 222068-222253 | 429332-429517 |  |
| Km3R16 | DR | 104 | 20239-20342 | 86115-86218 | 197106-197209 |

**Additional File 9. List of repeats larger than 100 bp found in the Km3 mtDNA**

^a^ DR and IR : direct and inverted repeats.

^b^ Boldface: IR copy.
