# Supplementary material for: Comparative analysis of mitochondrial genomes between a wheat K-type cytoplasmic male sterility (CMS) line and its maintainer line
Source: BMC Genomics. 2011 Mar 29;12:163. doi: 10.1186/1471-2164-12-163 (PMC3079663; doi:10.1186/1471-2164-12-163)
Supplement: Additional file 10 — Homology of mtDNA repeats between Ks3 and Km3. The file contains the list of homology of mtDNA repeats between Ks3 and Km3. Four repeats were almost identical in the Ks3 mtDNA and Km3 mtDNA, while the relationship between the large repeats in two mitochondrial genomes is complicated. [file 1471-2164-12-163-S10.DOCX]

| Ks3 ^a^ repeats | Repeats  size  (bp) | Ks3 mtDNA  Repeat coordinates | Km3 repeats | Repeats  size  (bp) | Homologous  Km3 mtDNA  Repeats coordinates | Nucleotide  sequence  Identity (%) |
| --- | --- | --- | --- | --- | --- | --- |
| Ks3R1-2 | 98977 | 1-1900 | Km3R3-**1** | 6064 | 1-1900 | 98 |
| Ks3R1-2 | 98977 | 1960-4627 | Km3R3-**1** | 6064 | 1890-4557 | 99 |
| Ks3R1-2 | 98977 | 1-1900 | Km3R4-**1** | 5469 | 1040-2939 | 98 |
| Ks3R1-2 | 98977 | 1960-4500 | Km3R4-**1** | 5469 | 2929-5469 | 99 |
| Ks3R1-2 | 98977 | 98593-98977 | Km3R11-**1** | 385 | 1-385 | 97 |
| Ks3R1-2 | 98977 | 85399-85584 | Km3R15-**1** | 186 | 1-186 | 100 |
| Ks3R2-2 | 64991 | 1-4819 | Km3R2-**1** | 7035 | 7035-2223 | 99 |
| Ks3R2-2 | 64991 | 4845-6752 | Km3R2-**1** | 7035 | 2197-290 | 99 |
| Ks3R2-2 | 64991 | 6769-7042 | Km3R2-**1** | 7035 | 279-1 | 97 |
| Ks3R2-2 | 64991 | 28553-30644 | Km3R6-**1** | 2460 | 2096-1 | 99 |
| Ks3R2-2 | 64991 | 1-1640 | Km3R1-**1** | 9881 | 1634-1 | 98 |
| Ks3R2-2 | 64991 | 62949-64991 | Km3R1-**1** | 9881 | 9881-7834 | 98 |
| Ks3R2-2 | 64991 | 1-1640 | Km3R8-**1** | 1634 | 1634-1 | 98 |
| Ks3R2-2 | 64991 | 64607-64991 | Km3R11-**1** | 385 | 385-1 | 97 |
| Ks3R3-1 | 33602 | 1-53 | Km3R16-**1** | 104 | 52-104 | 100 |
| Ks3R4-2 | 28476 | 2093-4633 | Km3R4-**1** | 5469 | 5469-2929 | 99 |
| Ks3R4-2 | 28476 | 4693-7637 | Km3R4-**1** | 5469 | 2939-1 | 98 |
| Ks3R4-2 | 28476 | 1966-4633 | Km3R3-**1** | 6064 | 4557-1890 | 99 |
| Ks3R4-2 | 28476 | 4693-6592 | Km3R3-**1** | 6064 | 1900-1 | 98 |
| Ks3R4-2 | 28476 | 18648-19871 | Km3R3-**1** | 6064 | 4568-5791 | 99 |
| Ks3R5-2 | 8853 | 1-2945 | Km3R4-**1** | 5469 | 1-2939 | 98 |
| Ks3R5-2 | 8853 | 3005-5545 | Km3R4-**1** | 5469 | 2929-5469 | 99 |
| Ks3R5-2 | 8853 | 1046-2945 | Km3R3-**1** | 6064 | 1-1900 | 98 |
| Ks3R5-2 | 8853 | 3005-5672 | Km3R3-**1** | 6064 | 1890-4557 | 99 |
| Ks3R6-3 | 7808 | 1-1900 | Km3R3-**1** | 6064 | 1-1900 | 98 |
| Ks3R6-3 | 7808 | 1960-4627 | Km3R3-**1** | 6064 | 1890-4557 | 99 |
| Ks3R6-3 | 7808 | 1-1900 | Km3R4-**1** | 5469 | 1040-2939 | 98 |
| Ks3R6-3 | 7808 | 1960-4500 | Km3R4-**1** | 5469 | 2929-5469 | 99 |

**Additional File 10. Homology of mtDNA repeats between** **Ks3 and Km3**

**Additional File 10. (continued)**

| Ks3 repeats | Repeats  size  (bp) | Ks3 mtDNA  Repeat coordinates | Km3 repeats | Repeats  size  (bp) | Homologous  Km3 mtDNA  Repeats coordinates | Nucleotide  sequence  Identity (%) |
| --- | --- | --- | --- | --- | --- | --- |
| Ks3R7-3 | 7637 | 2093-4633 | Km3R4-**1** | 5469 | 5469-2929 | 99 |
| Ks3R7-3 | 7637 | 4693-7637 | Km3R4-**1** | 5469 | 2939-1 | 98 |
| Ks3R7-3 | 7637 | 1966-4633 | Km3R3-**1** | 6064 | 4557-1890 | 99 |
| Ks3R7-3 | 7637 | 4693-6592 | Km3R3-**1** | 6064 | 1900-1 | 98 |
| Ks3R8-3 | 6592 | 1966-4633 | Km3R3-**1** | 6064 | 4557-1890 | 99 |
| Ks3R8-3 | 6592 | 4693-6952 | Km3R3-**1** | 6064 | 1900-1 | 98 |
| Ks3R8-3 | 6592 | 2093-4633 | Km3R4-**1** | 5469 | 5469-2929 | 99 |
| Ks3R8-3 | 6592 | 4693-6592 | Km3R4-**1** | 5469 | 2939-1040 | 98 |
| Ks3R9-2 | 4645 | 4220-4323 | Km3R16-**1** | 104 | 1-104 | 100 |
| Ks3R12-3 | 385 | 1-385 | Km3R11-**1** | 385 | 1-385 | 97 |
| Ks3R12-3 | 385 | 1-385 | Km3R1-**1** | 9881 | 7834-8218 | 97 |
| Ks3R13-4 | 391 | 1-391 | Km3R4-**1** | 5469 | 652-262 | 98 |
| Ks3R14-4 | 375 | 1-53 | Km3R16-**1** | 104 | 52-104 | 100 |
| Ks3R17-7 | 275 | 1-275 | Km3R3-**1** | 6064 | 4156-4430 | 95 |
| Ks3R17-7 | 275 | 1-275 | Km3R4-**1** | 5469 | 5195-5469 | 95 |
| Ks3R19-2 | 201 | 1-186 | Km3R15-**1** | 186 | 186-1 | 100 |
| Ks3R22-1 | 190 | 1-190 | Km3R14-**1** | 190 | 1-190 | 96 |
| Ks3R20-2 | 197 | 1-193 | Km3R13-**1** | 193 | 193-1 | 100 |
| Ks3R23-2 | 189 | 1-189 | Km3R1-**1** | 9881 | 9693-9881 | 97 |
| Ks3R24-2 | 185 | 1-185 | Km3R2-**1** | 7035 | 6186-6002 | 100 |
| Ks3R24-2 | 185 | 1-185 | Km3R8-**1** | 1634 | 785-601 | 100 |
| Ks3R24-2 | 185 | 1-185 | Km3R1-**1** | 9881 | 785-601 | 100 |
| Ks3R29-3 | 119 | 1-119 | Km3R1-**1** | 9881 | 9681-9799 | 96 |

^a^ Boldface: the No. of copy in the repeats.
