# Supplementary material for: Comparative analysis of mitochondrial genomes between a wheat K-type cytoplasmic male sterility (CMS) line and its maintainer line
Source: BMC Genomics. 2011 Mar 29;12:163. doi: 10.1186/1471-2164-12-163 (PMC3079663; doi:10.1186/1471-2164-12-163)
Supplement: Additional file 12 — Alignments of atp6 (A), nad6 (B), nad9 (C), and rps19-p (D) between Ks3 and Km3. the file contains the alignments of atp6 (A), nad6 (B), nad9 (C), and rps19-p (D) between Ks3 and Km3. The identical sequences are highlighted with a dark background. The vertical arrow in (D) indicates deletion in Ks3 mtDNA (Ks3_seq) and STOP shows the location of the terminal codon (TAG). [file 1471-2164-12-163-S12.PDF]

A

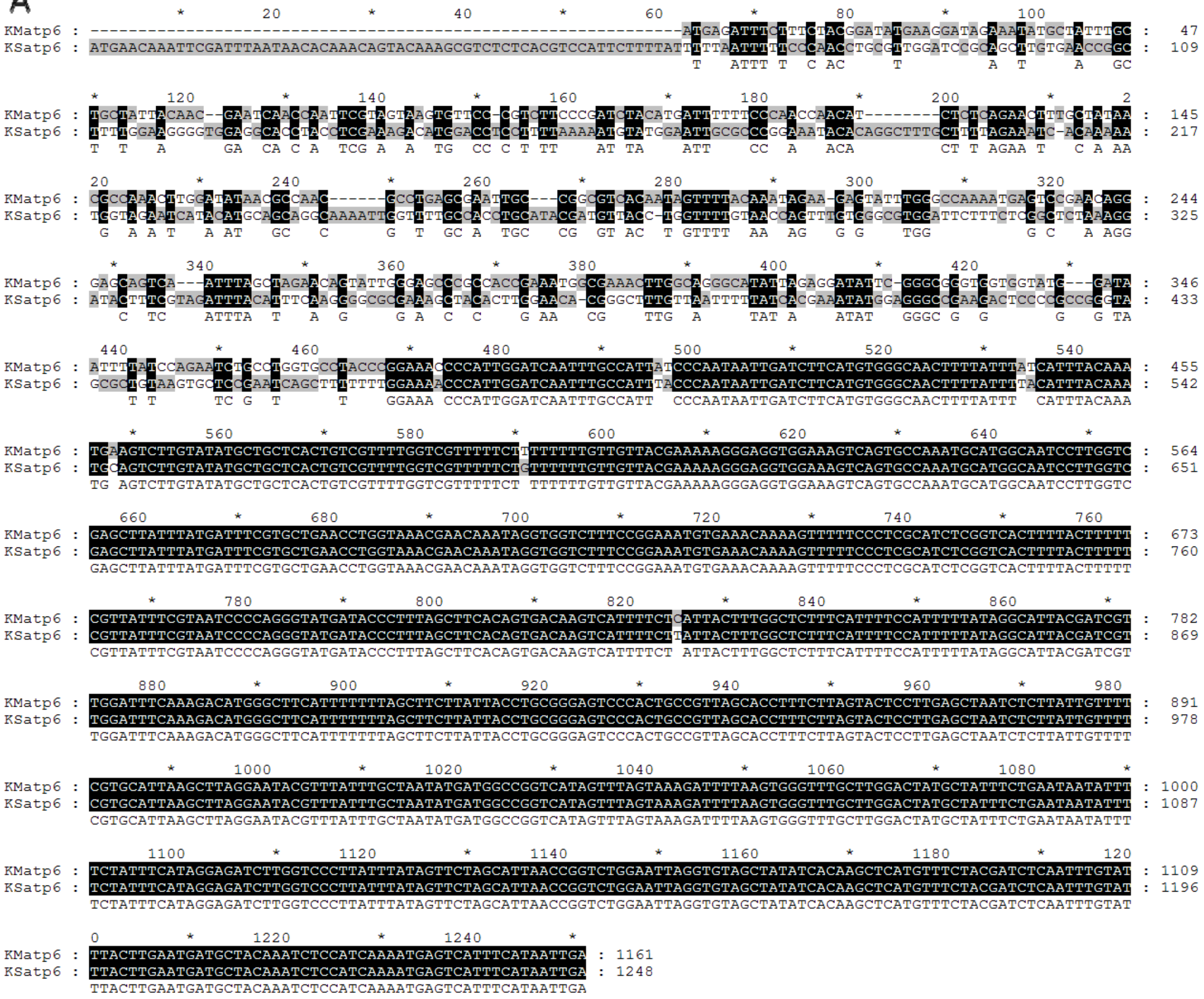

B

```
                   *        20         *        40         *        60         *        80         *       100         *
KMnad6 : ATGCGTCTTCTTGCTCCAGCATTCAAGTTCCATTTCAAGGGAGGACGACGTACCATGATACTTTCTGTTTTGTCGAGCCCTGCTTTGGTCTCTGGTTTGATGGTTGTACG : 110
KSnad6 : ATGCGTCTTCTTGCTCCAGCATTCAAGTTCCATTTCAAGGGAGGACGACGTACCATGATACTTTCTGTTTTGTCGAGCCCTGCTTTGGTCTCTGGTTTGATGGTTGTACG : 110
         ATGCGTCTTCTTGCTCCAGCATTCAAGTTCCATTTCAAGGGAGGACGACGTACCATGATACTTTCTGTTTTGTCGAGCCCTGCTTTGGTCTCTGGTTTGATGGTTGTACG

              120         *       140         *       160         *       180         *       200         *       220
KMnad6 : TGCTAAAAATCCGGTACATTCCGTTTTGTTTCCCATCCTAGTCTTTTGCGACACTTCTGGTTTACTTATTTTGTTAGGTCTCGACTTCTCCGCTATGATCTCCCCAGTAG : 220
KSnad6 : TGCTAAAAATCCGGTACATTCCGTTTTGTTTCCCATCCTAGTCTTTTGCGACACTTCTGGTTTACTTATTTTGTTAGGTCTCGACTTCTCCGCTATGATCTCCCCAGTAG : 220
         TGCTAAAAATCCGGTACATTCCGTTTTGTTTCCCATCCTAGTCTTTTGCGACACTTCTGGTTTACTTATTTTGTTAGGTCTCGACTTCTCCGCTATGATCTCCCCAGTAG

                   *       240         *       260         *       280         *       300         *       320         *
KMnad6 : TTCATATAGGAGCTATTGCCGTTTCATTCCTATTCGTGGTTATGATGTTCAATATTCAAATAGCGGAGATTCACGAAGAAGTATTGCGCTATTTACCAGTGAGTGGTATT : 330
KSnad6 : TTCATATAGGAGCTATTGCCGTTTCATTCCTATTCGTGGTTATGATGTTCAATATTCAAATAGCGGAGATTCACGAAGAAGTATTGCGCTATTTACCAGTGAGTGGTATT : 330
         TTCATATAGGAGCTATTGCCGTTTCATTCCTATTCGTGGTTATGATGTTCAATATTCAAATAGCGGAGATTCACGAAGAAGTATTGCGCTATTTACCAGTGAGTGGTATT

              340         *       360         *       380         *       400         *       420         *       440
KMnad6 : ATTGGACTGATCTTTTGGTGGGAAATGTTCTTCATTTTAGATAATGAAACCATTCCATTACTACCAACCCACAGAAATACGACCTCTCTGAGATATACGGTTTATGCCGG : 440
KSnad6 : ATTGGACTGATCTTTTGGTGGGAAATGTTCTTCATTTTAGATAATGAAACCATTCCATTACTACCAACCCACAGAAATACGACCTCTCTGAGATATACGGTTTATGCCGG : 440
         ATTGGACTGATCTTTTGGTGGGAAATGTTCTTCATTTTAGATAATGAAACCATTCCATTACTACCAACCCACAGAAATACGACCTCTCTGAGATATACGGTTTATGCCGG

                   *       460         *       480         *       500         *       520         *       540         *
KMnad6 : AAAGGTACGAAGTTGGACTAATTTGGAAACATTGGGCAATTTACTTTATACCTACTATTCCGTCTGGTTTTTGGTTTCTAGTCTGATTTTATTAGTTGCTATGATTGGGG : 550
KSnad6 : AAAGGTACGAAGTTGGACTAATTTGGAAACATTGGGCAATTTGCTTTATACCTACTATTCCGTCTGGTTTTTGGTTTCTAGTCTGATTTTATTAGTAGCTATGATTGGGG : 550
         AAAGGTACGAAGTTGGACTAATTTGGAAACATTGGGCAATTT CTTTATACCTACTATTCCGTCTGGTTTTTGGTTTCTAGTCTGATTTTATTAGT GCTATGATTGGGG

              560         *       580         *       600         *       620         *       640         *       660
KMnad6 : CTATAGTACTTACTATGCATAGGACTACAAAGGTGAAAAGACAGGATGTATTCCGACGAAATGCCTTGGATTCTAGGAGCCATATAATGAACAGGACTATTTCTCCTTTT : 660
KSnad6 : CTATAGTACTTACTATGCATAGGACTACAAAGGTGAAAAGACAGGATGTATTCCGACGAAATGCCTTGGATTCTAGGAGGAATATAATGAACAGGACTATTTCTCCTTTT : 660
         CTATAGTACTTACTATGCATAGGACTACAAAGGTGAAAAGACAGGATGTATTCCGACGAAATGCCTTGGATTCTAGGAG  ATATAATGAACAGGACTATTTCTCCTTTT

                   *       680         *       700         *       720         *       740         *       760         *
KMnad6 : GGCCATAGCCATAGAAGAAGCTTCTCCTCCGGAGCGGGGGGACCG---CCTGACAATTACAAAGAAACCTTTAAAATGTGGATTTAG----------------------- : 744
KSnad6 : GGCCATAGCCATAGAAGAAGCTTCTCCTCCAAAGCGGAGGGAGGGGAATCTCAGGATTCCTATGACCCTGCTTATATCTCTTTTTTAAGAGCTCGTTTAGGGTTTTCCC : 770
         GGCCATAGCCATAGAAGAAGCTTCTCCTCC  AGCGG GGGA  G    CT A  ATT C A GA  C   T A AT T   TTT

              780         *       800         *       820         *       840         *       860         *       880
KMnad6 : -------------------------------------------------------------------------------------------------------------- :   -
KSnad6 : GGGTTTGGTACCAAATCTGGATAAACTCCTTTCGGTTCTTAAACCTGAGGAAATTCTATTTCTGGCTTTTCGCTTCCCGCGGGATGCGAACATGTTTAAATTGCCCCCTA : 880

                   *       900         *
KMnad6 : -------------------------------------- :   -
KSnad6 : AAGAAGATCAAAGATATGATTCAAGAAGCAATGGCTAA : 918
```

C

```
                     *        20         *        40         *        60         *        80         *       100
KMrps19-p : AGAGAAAGTCTGAGGAGCAGGAAAATTTGGTCACGTAGATCTTCTATTTCGCCGGAATTCGTTGATTGCTCCGTACTCATTTACAATGGAAAAACTCCTGTTCGTTGTA : 109
KSrps19-p : AGAGAAAGTCTGAGGAGCAGGAAAATTTGGTCACGTAGATCTTCTATTTCGCCGGAATTCGTTGATTGCTCCGTACTCATTTACAATGGAAAAACTCCTGTTCGTTGTA : 109
            AGAGAAAGTCTGAGGAGCAGGAAAATTTGGTCACGTAGATCTTCTATTTCGCCGGAATTCGTTGATTGCTCCGTACTCATTTACAATGGAAAAACTCCTGTTCGTTGTA

            *        120         *       140         *       160         *       180         *       200
KMrps19-p : AGATCACTGAAGG---------TCATAAATTTGGAGAGTTTGCTTTTACACGGAGACGAAGACCCTATCGAACAAATAGAGGAAAGGGGAAAAAGTAA : 198
KSrps19-p : AGATCACTGAAGGAAAGGTTGGTCATAAATTTGGAGAGTTTGCTTTTACACGGAGACGAAGACCCTATCGAACAAATAGAGGAAAGGGGAAAAAGTAA : 207
            AGATCACTGAAGG         TCATAAATTTGGAGAGTTTGCTTTTACACGGAGACGAAGACCCTATCGAACAAATAGAGGAAAGGGGAAAAAGTAA
```

D

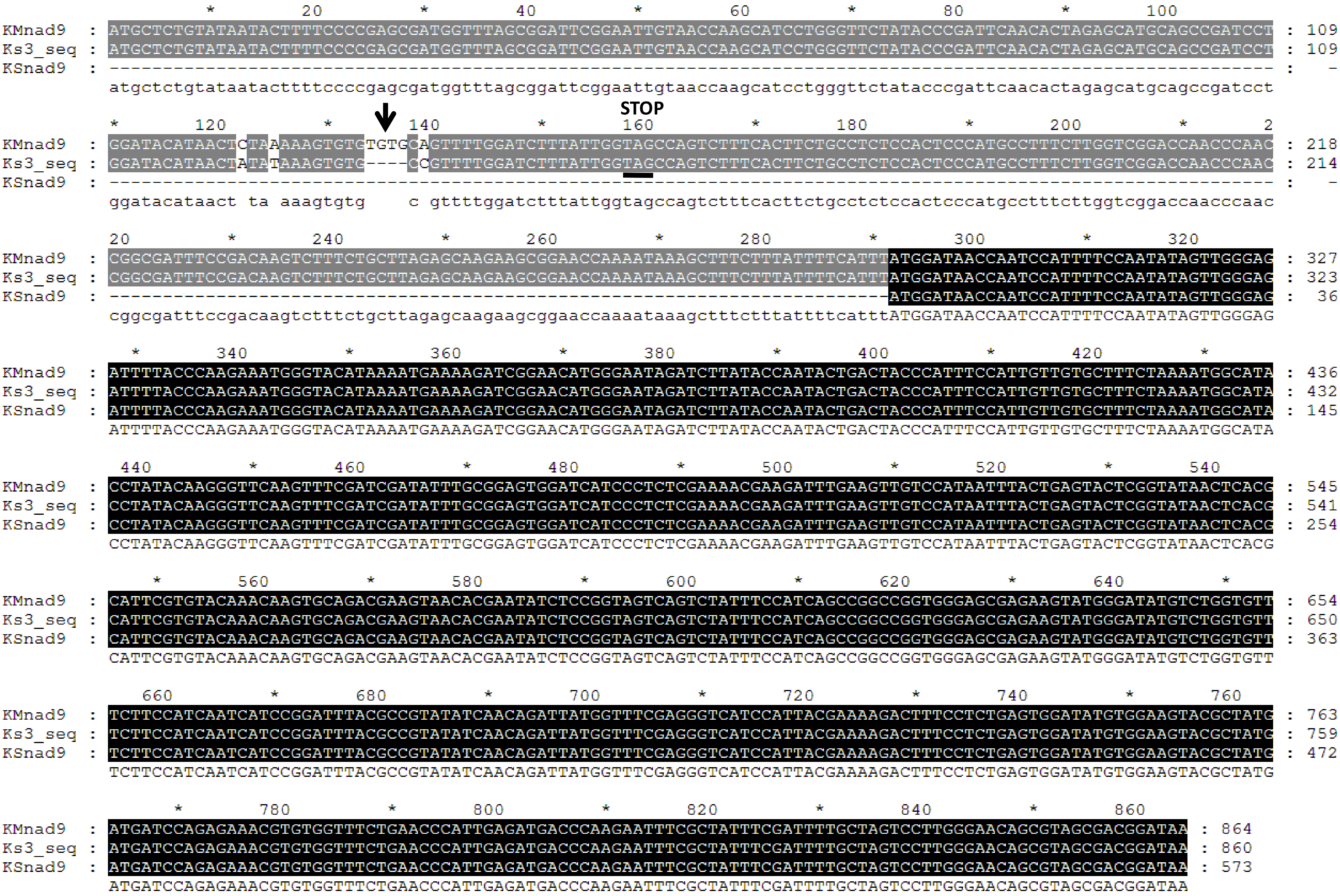

* 20 * 40 * 60 * 80 * 100
KMnad9 : ATGCTCTGTATAATACTTTTCCCCGAGCGATGGTTTAGCGGATTCGGAATTGTAACCAAGCATCCTGGGTTCTATACCCGATTCAACACTAGAGCATGCAGCCGATCCT : 109
Ks3_seq : ATGCTCTGTATAATACTTTTCCCCGAGCGATGGTTTAGCGGATTCGGAATTGTAACCAAGCATCCTGGGTTCTATACCCGATTCAACACTAGAGCATGCAGCCGATCCT : 109
KSnad9 : --- : -
atgctctgtataatacttttccccgagcgatggtttagcggattcggaattgtaaccaagcatcctgggttctatacccgattcaacactagagcatgcagccgatcct
STOP
* 120 * 140 * 160 * 180 * 200 * 2
KMnad9 : GGATACATAACTCTAAAAAGTGTGTGTGCAGTTTTGGATCTTTATTGGTAGCCAGTCTTTCACTTCTGCCTCTCCACTCCCATGCCTTTCTTGGTCGGACCAACCCAAC : 218
Ks3_seq : GGATACATAACTATATAAAGTGTG----CCGTTTTGGATCTTTATTGGTAGCCAGTCTTTCACTTCTGCCTCTCCACTCCCATGCCTTTCTTGGTCGGACCAACCCAAC : 214
KSnad9 : --- : -
ggatacataact ta aaagtgtg c gttttggatctttattggtagccagtctttcacttctgcctctccactcccatgcctttcttggtcggaccaacccaac
20 * 240 * 260 * 280 * 300 * 320
KMnad9 : CGGCGATTTCCGACAAGTCTTTCTGCTTAGAGCAAGAAGCGGAACCAAAATAAAGCTTTCTTTATTTTCATTTATGGATAACCAATCCATTTTCCAATATAGTTGGGAG : 327
Ks3_seq : CGGCGATTTCCGACAAGTCTTTCTGCTTAGAGCAAGAAGCGGAACCAAAATAAAGCTTTCTTTATTTTCATTTATGGATAACCAATCCATTTTCCAATATAGTTGGGAG : 323
KSnad9 : ---ATGGATAACCAATCCATTTTCCAATATAGTTGGGAG : 36
cggcgatttccgacaagtctttctgcttagagcaagaagcggaaccaaaataaagctttctttattttcatttATGGATAACCAATCCATTTTCCAATATAGTTGGGAG
* 340 * 360 * 380 * 400 * 420 *
KMnad9 : ATTTACCCAAGAAATGGGTACATAAAATGAAAAGATCGGAACATGGGAATAGATCTTATACCAATACTGACTACCCATTTCCATTGTTGTGCTTTCTAAAATGGCATA : 436
Ks3_seq : ATTTACCCAAGAAATGGGTACATAAAATGAAAAGATCGGAACATGGGAATAGATCTTATACCAATACTGACTACCCATTTCCATTGTTGTGCTTTCTAAAATGGCATA : 432
KSnad9 : ATTTACCCAAGAAATGGGTACATAAAATGAAAAGATCGGAACATGGGAATAGATCTTATACCAATACTGACTACCCATTTCCATTGTTGTGCTTTCTAAAATGGCATA : 145
ATTTACCCAAGAAATGGGTACATAAAATGAAAAGATCGGAACATGGGAATAGATCTTATACCAATACTGACTACCCATTTCCATTGTTGTGCTTTCTAAAATGGCATA
440 * 460 * 480 * 500 * 520 * 540
KMnad9 : CCTATACAAGGGTTCAAGTTTCGATCGATATTTGCGGAGTGGATCATCCCTCTCGAAAACGAAGATTTGAAGTTGTCCATAATTTACTGAGTACTCGGTATAACTCACG : 545
Ks3_seq : CCTATACAAGGGTTCAAGTTTCGATCGATATTTGCGGAGTGGATCATCCCTCTCGAAAACGAAGATTTGAAGTTGTCCATAATTTACTGAGTACTCGGTATAACTCACG : 541
KSnad9 : CCTATACAAGGGTTCAAGTTTCGATCGATATTTGCGGAGTGGATCATCCCTCTCGAAAACGAAGATTTGAAGTTGTCCATAATTTACTGAGTACTCGGTATAACTCACG : 254
CCTATACAAGGGTTCAAGTTTCGATCGATATTTGCGGAGTGGATCATCCCTCTCGAAAACGAAGATTTGAAGTTGTCCATAATTTACTGAGTACTCGGTATAACTCACG
* 560 * 580 * 600 * 620 * 640 *
KMnad9 : CATTCGTGTACAAACAAGTGCAGACGAAGTAACACGAATATCTCCGGTAGTCAGTCTATTTCCATCAGCCGGCCGGTGGGAGCGAGAAGTATGGGATATGTCTGGTGTT : 654
Ks3_seq : CATTCGTGTACAAACAAGTGCAGACGAAGTAACACGAATATCTCCGGTAGTCAGTCTATTTCCATCAGCCGGCCGGTGGGAGCGAGAAGTATGGGATATGTCTGGTGTT : 650
KSnad9 : CATTCGTGTACAAACAAGTGCAGACGAAGTAACACGAATATCTCCGGTAGTCAGTCTATTTCCATCAGCCGGCCGGTGGGAGCGAGAAGTATGGGATATGTCTGGTGTT : 363
CATTCGTGTACAAACAAGTGCAGACGAAGTAACACGAATATCTCCGGTAGTCAGTCTATTTCCATCAGCCGGCCGGTGGGAGCGAGAAGTATGGGATATGTCTGGTGTT
660 * 680 * 700 * 720 * 740 * 760
KMnad9 : TCTTCCATCAATCATCCGGATTTACGCCGTATATCAACAGATTATGGTTTCGAGGGTCATCCATTACGAAAAGACTTTCCTCTGAGTGGATATGTGGAAGTACGCTATG : 763
Ks3_seq : TCTTCCATCAATCATCCGGATTTACGCCGTATATCAACAGATTATGGTTTCGAGGGTCATCCATTACGAAAAGACTTTCCTCTGAGTGGATATGTGGAAGTACGCTATG : 759
KSnad9 : TCTTCCATCAATCATCCGGATTTACGCCGTATATCAACAGATTATGGTTTCGAGGGTCATCCATTACGAAAAGACTTTCCTCTGAGTGGATATGTGGAAGTACGCTATG : 472
TCTTCCATCAATCATCCGGATTTACGCCGTATATCAACAGATTATGGTTTCGAGGGTCATCCATTACGAAAAGACTTTCCTCTGAGTGGATATGTGGAAGTACGCTATG
* 780 * 800 * 820 * 840 * 860
KMnad9 : ATGATCCAGAGAAACGTGTGGTTTCTGAACCCATTGAGATGACCCAAGAATTTCGCTATTTCGATTTTGCTAGTCCTTGGGAACAGCGTAGCGACGGATAA : 864
Ks3_seq : ATGATCCAGAGAAACGTGTGGTTTCTGAACCCATTGAGATGACCCAAGAATTTCGCTATTTCGATTTTGCTAGTCCTTGGGAACAGCGTAGCGACGGATAA : 860
KSnad9 : ATGATCCAGAGAAACGTGTGGTTTCTGAACCCATTGAGATGACCCAAGAATTTCGCTATTTCGATTTTGCTAGTCCTTGGGAACAGCGTAGCGACGGATAA : 573
ATGATCCAGAGAAACGTGTGGTTTCTGAACCCATTGAGATGACCCAAGAATTTCGCTATTTCGATTTTGCTAGTCCTTGGGAACAGCGTAGCGACGGATAA
