# Supplementary material for: Comparative analysis of mitochondrial genomes between a wheat K-type cytoplasmic male sterility (CMS) line and its maintainer line
Source: BMC Genomics. 2011 Mar 29;12:163. doi: 10.1186/1471-2164-12-163 (PMC3079663; doi:10.1186/1471-2164-12-163)
Supplement: Additional file 13 — MultiPipMaker analysis of the mtDNA of several angiosperms. Ks3 mtDNA was used as the reference genome for comparison with those of Km3, rice, maize, Arabidopsis thaliana, and rapeseed. (A) Positions of genes or exons of Ks3 mtDNA are indicated with black bars, and their orientations are shown with arrows. The percentage identity is shown at the right side. (B) A schematic representation of (A) in which only representative genes of Ks3 mtDNA are marked. Red and green bars indicate identity with Km3 mtDNA and other mtDNAs, respectively. [file 1471-2164-12-163-S13.PDF]

A

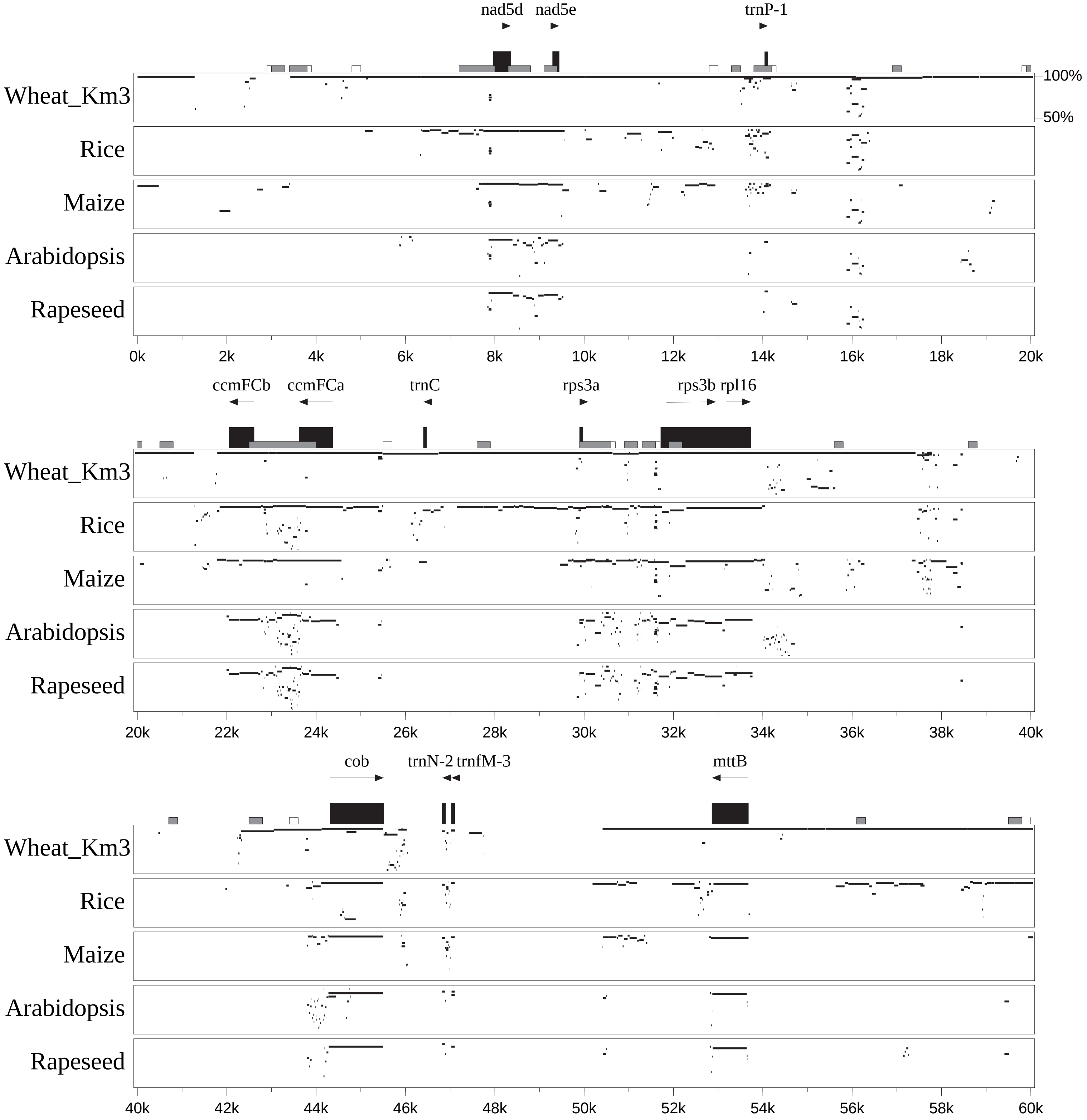

nad5d
nad5e
trnP-1
Wheat_Km3
Rice
Maize
Arabidopsis
Rapeseed
100%
50%
0k
2k
4k
6k
8k
10k
12k
14k
16k
18k
20k
ccmFCb
ccmFCa
trnC
rps3a
rps3b
rpl16
22k
24k
26k
28k
30k
32k
34k
36k
38k
40k
cob
trnN-2
trnfM-3
mttB
42k
44k
46k
48k
50k
52k
54k
56k
58k
60k

A (continued)

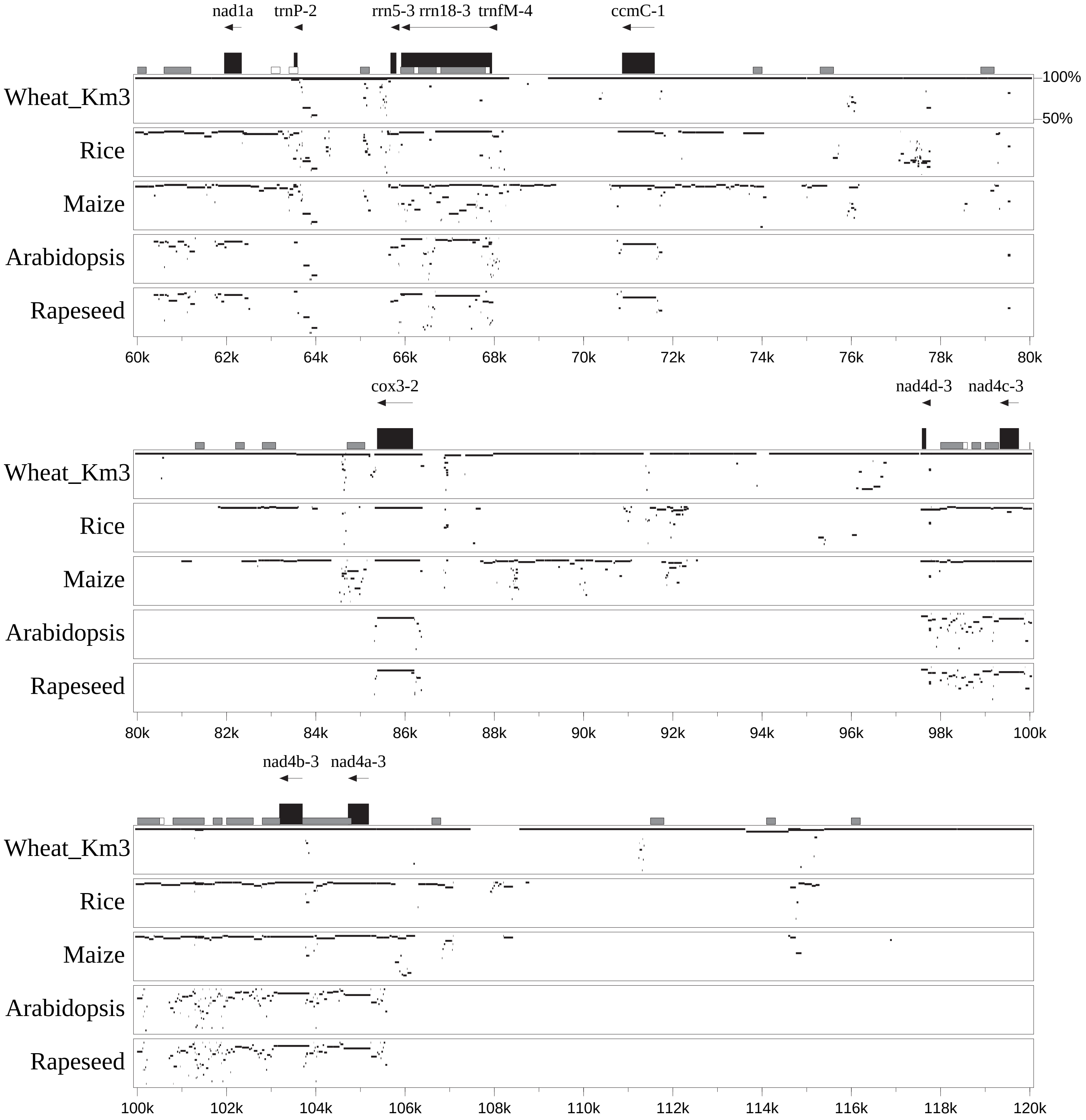
nad1a
trnP-2
rrn5-3
rrn18-3
trnfM-4
ccmC-1
Wheat_Km3
Rice
Maize
Arabidopsis
Rapeseed
100%
50%
60k
62k
64k
66k
68k
70k
72k
74k
76k
78k
80k
cox3-2
nad4d-3
nad4c-3
80k
82k
84k
86k
88k
90k
92k
94k
96k
98k
100k
nad4b-3
nad4a-3
100k
102k
104k
106k
108k
110k
112k
114k
116k
118k
120k

A (continued)

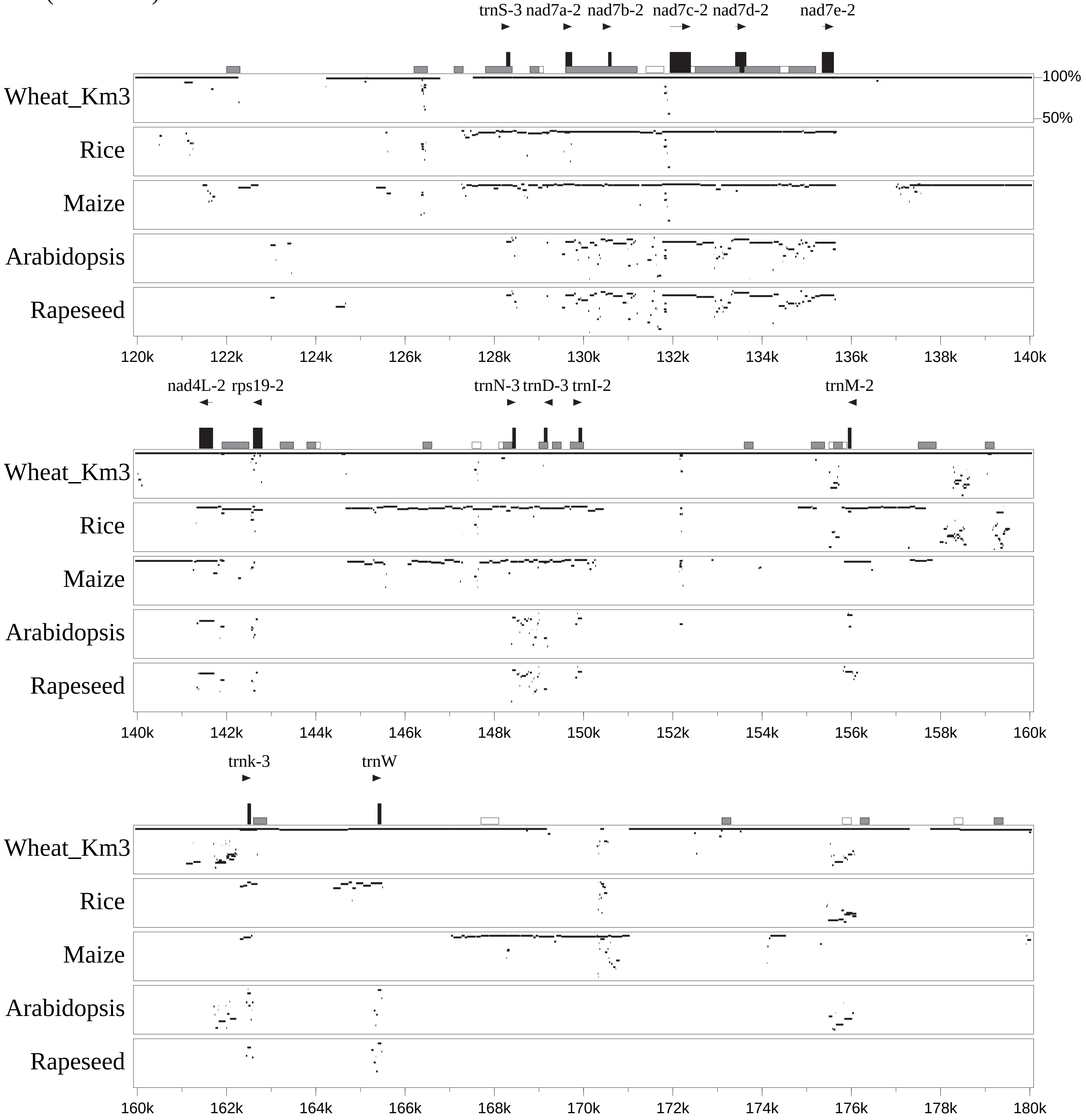

trnS-3
nad7a-2
nad7b-2
nad7c-2
nad7d-2
nad7e-2
100%
50%
Wheat_Km3
Rice
Maize
Arabidopsis
Rapeseed
120k
122k
124k
126k
128k
130k
132k
134k
136k
138k
140k
nad4L-2
rps19-2
trnN-3
trnD-3
trnI-2
trnM-2
Wheat_Km3
Rice
Maize
Arabidopsis
Rapeseed
140k
142k
144k
146k
148k
150k
152k
154k
156k
158k
160k
trnk-3
trnW
Wheat_Km3
Rice
Maize
Arabidopsis
Rapeseed
160k
162k
164k
166k
168k
170k
172k
174k
176k
178k
180k

A (continued)

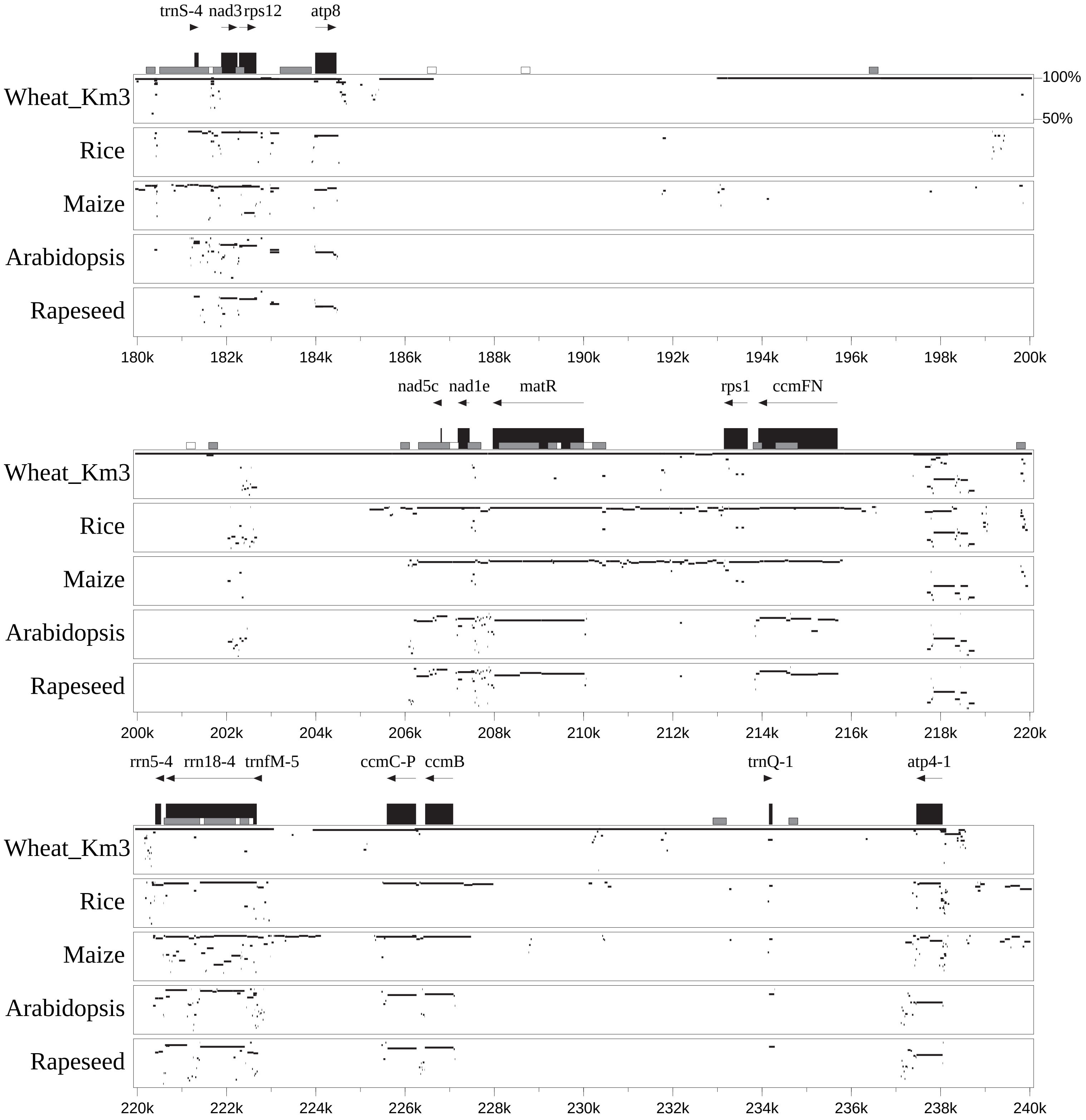
trnS-4
nad3
rps12
atp8
Wheat_Km3
Rice
Maize
Arabidopsis
Rapeseed
100%
50%
180k
182k
184k
186k
188k
190k
192k
194k
196k
198k
200k
nad5c
nad1e
matR
rps1
ccmFN
202k
204k
206k
208k
210k
212k
214k
216k
218k
220k
rrn5-4
rrn18-4
trnfM-5
ccmC-P
ccmB
trnQ-1
atp4-1
222k
224k
226k
228k
230k
232k
234k
236k
238k
240k

A (continued)

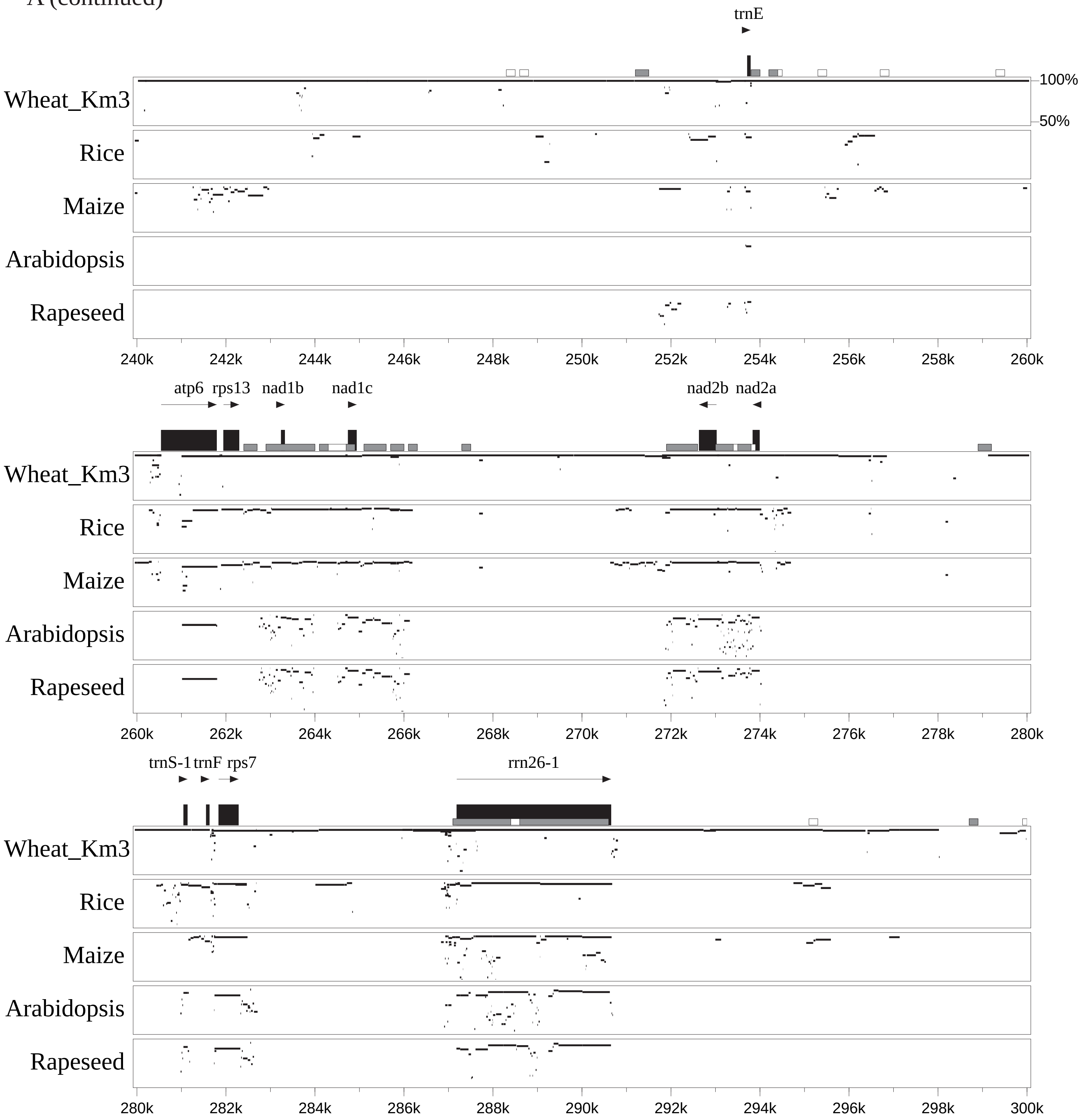
trnE
Wheat_Km3
Rice
Maize
Arabidopsis
Rapeseed
100%
50%
240k
242k
244k
246k
248k
250k
252k
254k
256k
258k
260k
atp6
rps13
nad1b
nad1c
nad2b
nad2a
262k
264k
266k
268k
270k
272k
274k
276k
278k
280k
trnS-1
trnF
rps7
rrn26-1
282k
284k
286k
288k
290k
292k
294k
296k
298k
300k

A (continued)

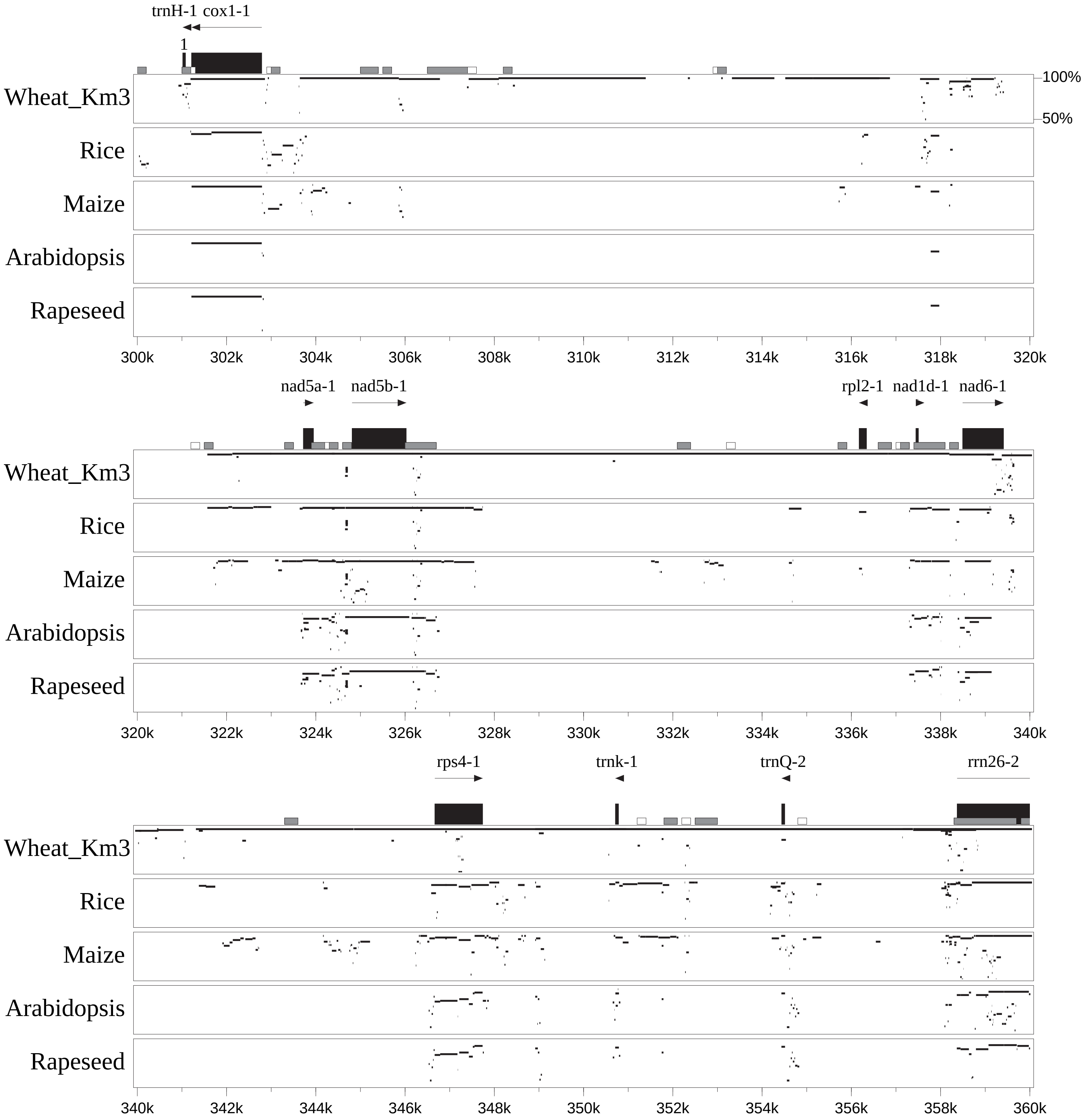
trnH-1
cox1-1
1
Wheat_Km3
Rice
Maize
Arabidopsis
Rapeseed
100%
50%
300k
302k
304k
306k
308k
310k
312k
314k
316k
318k
320k
nad5a-1
nad5b-1
rpl2-1
nad1d-1
nad6-1
320k
322k
324k
326k
328k
330k
332k
334k
336k
338k
340k
rps4-1
trnk-1
trnQ-2
rrn26-2
340k
342k
344k
346k
348k
350k
352k
354k
356k
358k
360k

A (continued)

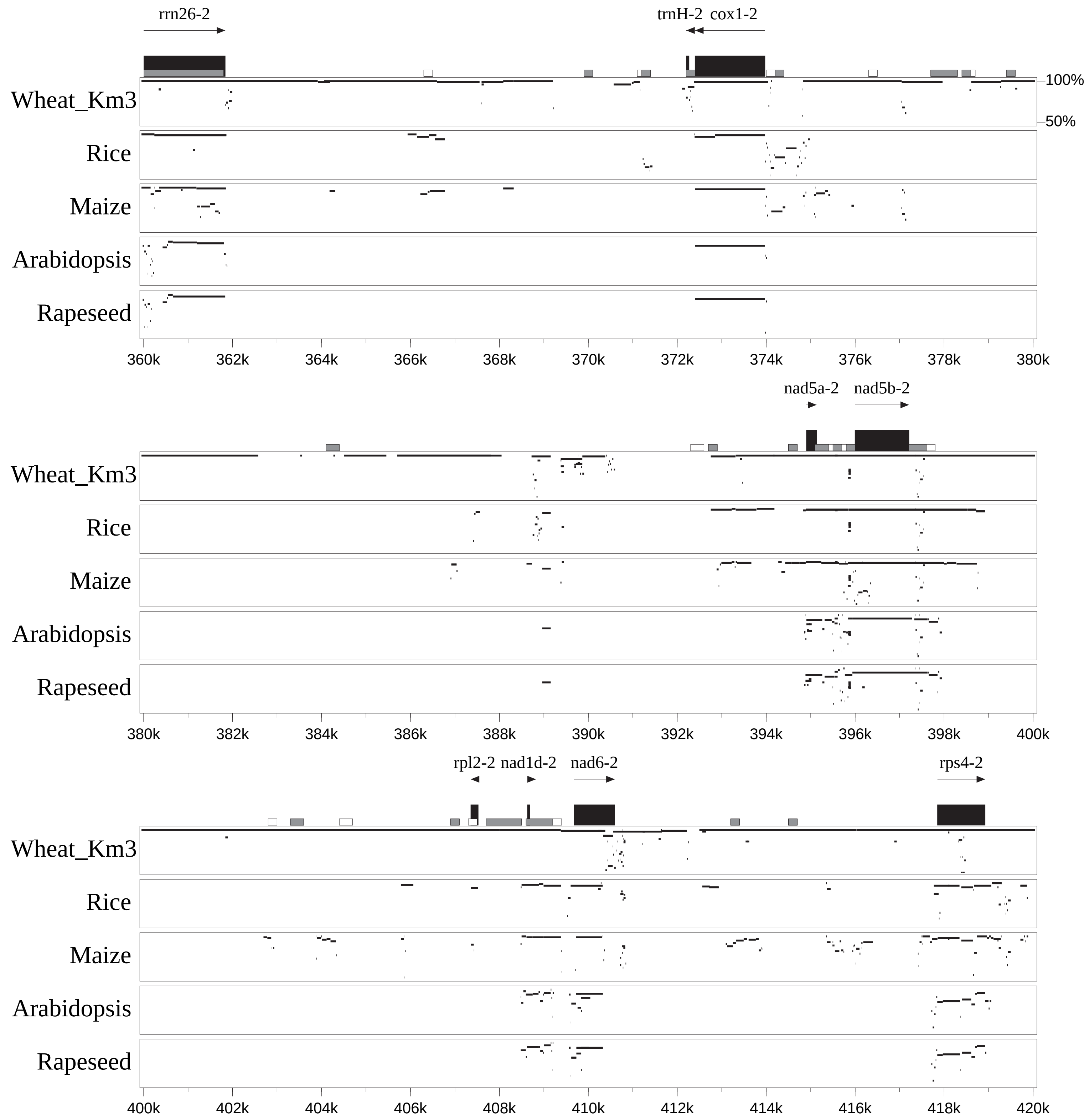

rrn26-2
trnH-2
cox1-2
Wheat_Km3
Rice
Maize
Arabidopsis
Rapeseed
100%
50%
360k
362k
364k
366k
368k
370k
372k
374k
376k
378k
380k
nad5a-2
nad5b-2
380k
382k
384k
386k
388k
390k
392k
394k
396k
398k
400k
rpl2-2
nad1d-2
nad6-2
rps4-2
400k
402k
404k
406k
408k
410k
412k
414k
416k
418k
420k

A (continued)

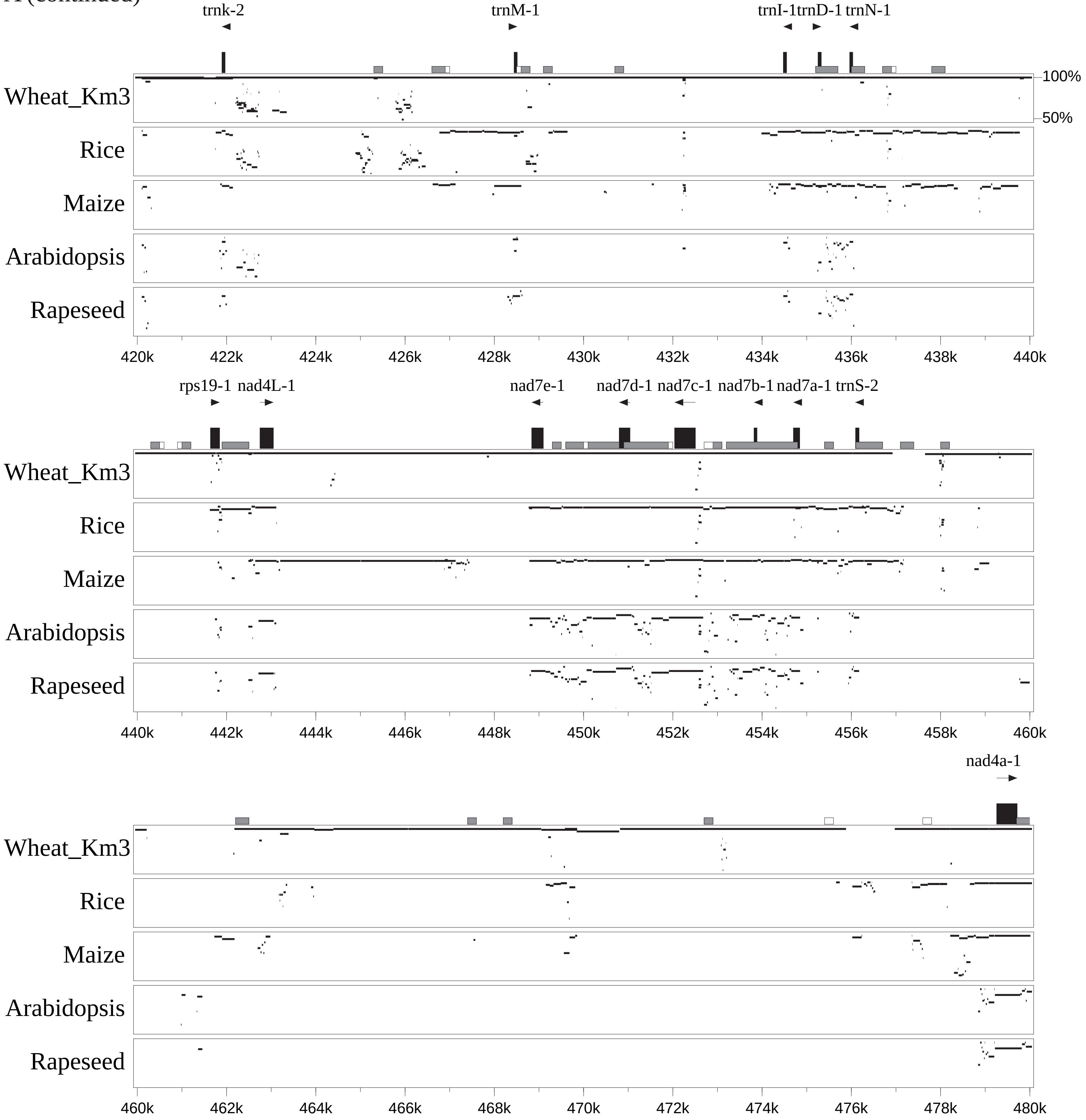

trnk-2
trnM-1
trnI-1
trnD-1
trnN-1
100%
50%
Wheat_Km3
Rice
Maize
Arabidopsis
Rapeseed
420k
422k
424k
426k
428k
430k
432k
434k
436k
438k
440k
rps19-1
nad4L-1
nad7e-1
nad7d-1
nad7c-1
nad7b-1
nad7a-1
trnS-2
Wheat_Km3
Rice
Maize
Arabidopsis
Rapeseed
440k
442k
444k
446k
448k
450k
452k
454k
456k
458k
460k
nad4a-1
Wheat_Km3
Rice
Maize
Arabidopsis
Rapeseed
460k
462k
464k
466k
468k
470k
472k
474k
476k
478k
480k

A (continued)

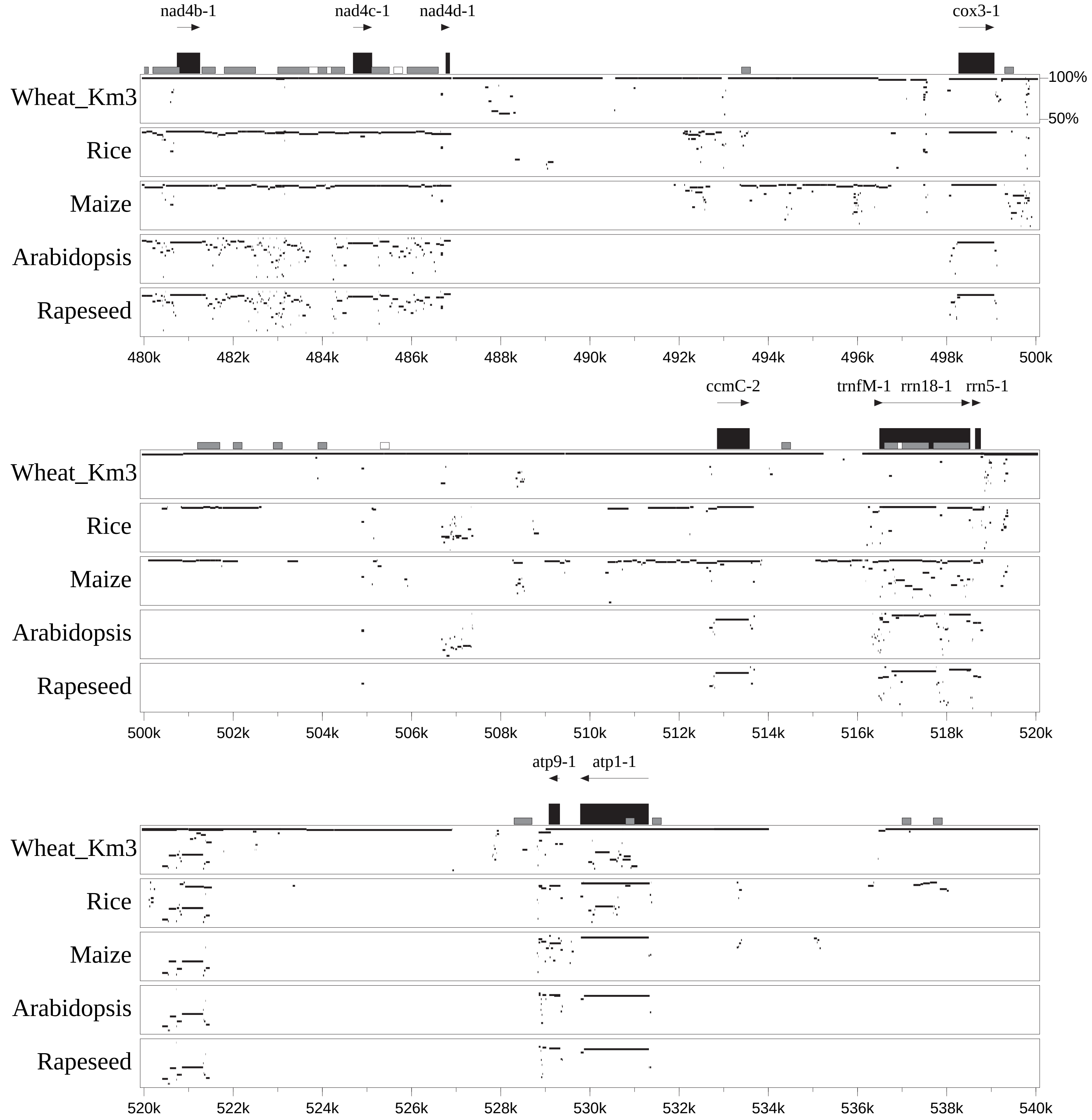

nad4b-1
nad4c-1
nad4d-1
cox3-1
100%
50%
Wheat_Km3
Rice
Maize
Arabidopsis
Rapeseed
480k
482k
484k
486k
488k
490k
492k
494k
496k
498k
500k
ccmC-2
trnfM-1
rrn18-1
rrn5-1
500k
502k
504k
506k
508k
510k
512k
514k
516k
518k
520k
atp9-1
atp1-1
520k
522k
524k
526k
528k
530k
532k
534k
536k
538k
540k

A (continued)

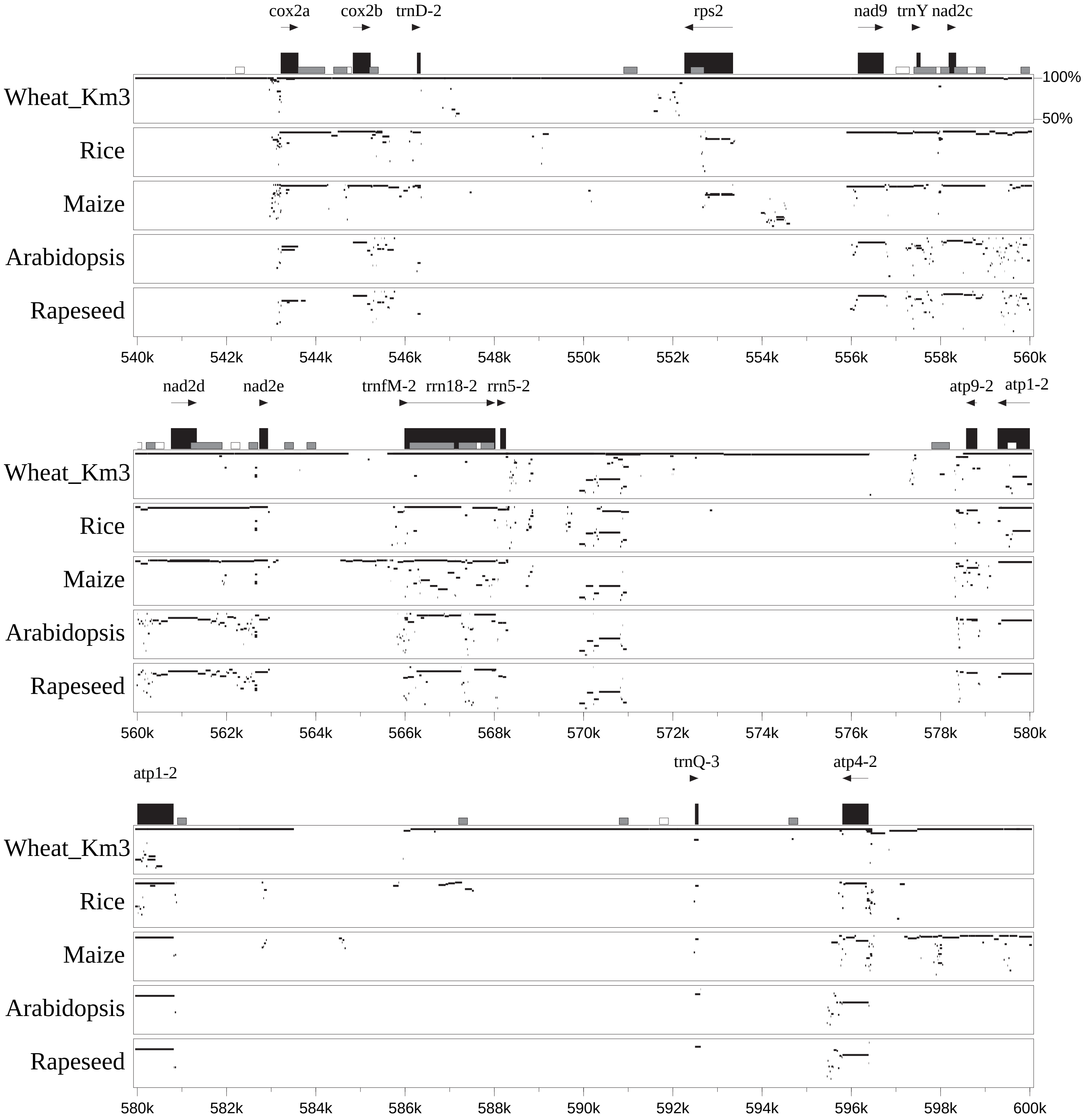
cox2a
cox2b
trnD-2
rps2
nad9
trnY
nad2c
Wheat_Km3
Rice
Maize
Arabidopsis
Rapeseed
100%
50%
540k
542k
544k
546k
548k
550k
552k
554k
556k
558k
560k
nad2d
nad2e
trnfM-2
rrn18-2
rrn5-2
atp9-2
atp1-2
562k
564k
566k
568k
570k
572k
574k
576k
578k
580k
trnQ-3
atp4-2
582k
584k
586k
588k
590k
592k
594k
596k
598k
600k

A (continued)

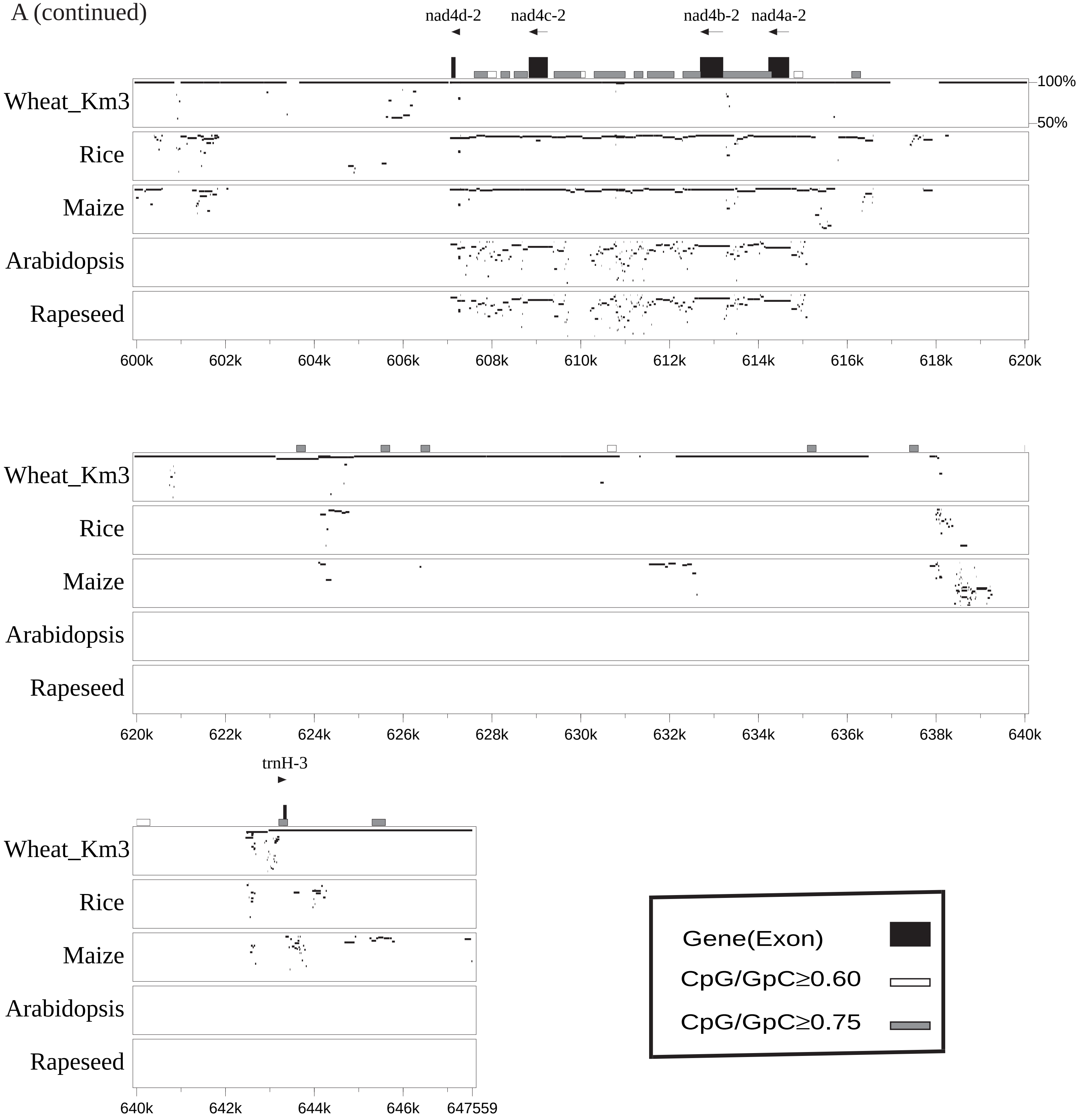

nad4d-2
nad4c-2
nad4b-2
nad4a-2
100%
50%
Wheat_Km3
Rice
Maize
Arabidopsis
Rapeseed
600k
602k
604k
606k
608k
610k
612k
614k
616k
618k
620k
622k
624k
626k
628k
630k
632k
634k
636k
638k
640k
trnH-3
642k
644k
646k
647559
Gene(Exon)
CpG/GpC≥0.60
CpG/GpC≥0.75

B

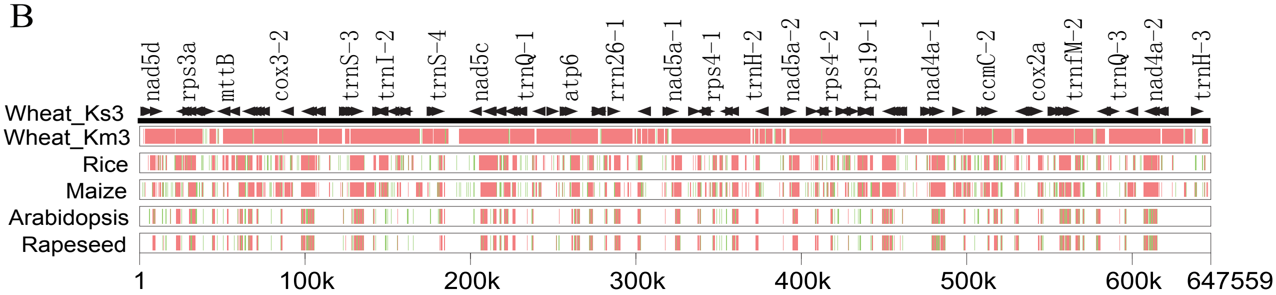

nad5d
rps3a
mttB
cox3-2
trnS-3
trnI-2
trnS-4
nad5c
trnQ-1
atp6
rrn26-1
nad5a-1
rps4-1
trnH-2
nad5a-2
rps4-2
rps19-1
nad4a-1
ccmC-2
cox2a
trnfM-2
trnQ-3
nad4a-2
trnH-3
Wheat_Ks3
Wheat_Km3
Rice
Maize
Arabidopsis
Rapeseed
1
100k
200k
300k
400k
500k
600k
647559
