# Supplementary material for: Comparative analysis of mitochondrial genomes between a wheat K-type cytoplasmic male sterility (CMS) line and its maintainer line
Source: BMC Genomics. 2011 Mar 29;12:163. doi: 10.1186/1471-2164-12-163 (PMC3079663; doi:10.1186/1471-2164-12-163)
Supplement: Additional file 14 — The number of copies of mitochondrial genes in Ks3, Km3, maize, and rice. The file contains the list of the number of copies of mitochondrial genes in Ks3, Km3, maize, and rice. The identity of genes between Ks3 mtDNA and Km3 mtDNA was also shown. [file 1471-2164-12-163-S14.DOCX]

**Additional File 14. The number of copies of mitochondrial genes in Ks3, Km3, maize, and rice**

| Gene | Total number of copies***^e^***  Ks3 Km3 | | Rice | Maize | Difference between  Ks3 and Km3 |
| --- | --- | --- | --- | --- | --- |
| Complex V |  |  |  |  |  |
| *atp1* | 2 | 1 | 2 | 2 | 1-bp mismatch^c^ |
| *atp4* | 2 | 1 | 2 | 1 | 3-bp mismatch |
| *atp6* | 1 | 2 | 1 | 1 | # |
| *atp8* | 1 | 2 | 1 | 1 | identical^d^ |
| *atp9* | 2 | 1 | 1 | 1 | identical |
| Cytochrome c biogenesis genes |  |  |  |  |  |
| *ccmB* | 1 | 1 | 1 | 1 | identical |
| *ccmC* | 2+***ccmC-p*** | 1 | 1 | 1 | identical |
| *ccmFC* | 1 | 1 | 1 | 1 | identical |
| *ccmFN* | 1 | 1 | 1 | 1 | 3-bp mismatch |
| Complex Ⅲ&Ⅳ genes |  |  |  |  |  |
| *cob* | 1 | 1 | 1 | 1 | identical |
| *cox1* | 2 | 1 | 1 | 1 | 2-bp mismatch |
| *cox2* | 1 | 1 | 1 | 1 | identical |
| *cox3* | 2 | 1 | 2 | 1 | 3-bp mismatch; |

**Additional File 14.** (continued)

| Gene | Total number of copies  Ks3 Km3 | | Rice | Maize | Difference between  Ks3 and Km3 |
| --- | --- | --- | --- | --- | --- |
| Other protein coding genes |  |  |  |  |  |
| *matR* | 1 | 1 | 1 | 1 | 1-bp mismatch |
| *mttB* | 1 | 1 | 1 | 1 | identical |
| Complex Ⅰgenes |  |  |  |  |  |
| *nad1a* | 1 | 1 | 2 | 2 | identical |
| *nad1b* | 1 | 1 | 1 | 1 | identical |
| *nad1c* | 1 | 1 | 1 | 1 | identical |
| *nad1d* | 2 | 1 | 1 | 1 | identical |
| *nad1e* | 1 | 1 | 1 | 1 | identical |
| *nad2a* | 1 | 1 | 1 | 1 | identical |
| *nad2b* | 1 | 1 | 1 | 1 | identical |
| *nad2c* | 1 | 1 | 2 | 1 | identical |
| *nad2d* | 1 | 1 | 2 | 2 | identical |
| *nad2e* | 1 | 1 | 2 | 2 | identical |
| *nad3* | 1 | 1 | 1 | 1 | 1-bp mismatch |
| *nad4a* | 3 | 1 | 1 | 1 | identical |
| *nad4b* | 3 | 1 | 1 | 1 | 2-bp mismatch |
| *nad4c* | 3 | 1 | 1 | 1 | 1-bp mismatch |
| *nad4d* | 3 | 1 | 3 | 1 | 1-bp mismatch |
| *nad4L* | 2 | 1 | 1 | 1 | identical |
| *nad5a* | 2 | 1 | 2 | 1 | identical |
| *nad5b* | 2 | 1 | 2 | 1 | identical |
| *nad5c* | 1 | 1 | 1 | 1 | identical |
| *nad5d* | 1 | 1 | 1 | 1 | identical |

**Additional File 14.** (continued)

| Gene | Total number of copies  Ks3 Km3 | | Rice | Maize | Difference between  Ks3 and Km3 |
| --- | --- | --- | --- | --- | --- |
| *nad5e* | 1 | 1 | 1 | 1 | identical |
| *nad6* | 2 | 1 | 1 | 1 | # |
| *nad7a* | 2 | 1 | 1 | 1 | identical |
| *nad7b* | 2 | 1 | 1 | 1 | identical |
| *nad7c* | 2 | 1 | 1 | 1 | identical |
| *nad7d* | 2 | 1 | 1 | 1 | identical |
| *nad7e* | 2 | 1 | 1 | 1 | identical |
| *nad9* | 1 | 1 | 2 | 1 | # |
| Ribosomal protein genes |  |  |  |  |  |
| *rpl5* | 0 | 1 | 2 | 0 |  |
| *rpl16* | 1 | 1 | 1 | 1 | identical |
| *rpl2* | 2(***rpl2-p***) | 1(***rpl2-p***) | 3 | 0 | identical |
| *rps1* | 1 | 1 | 1 | 1 | 1-bp mismatch |
| *rps12* | 1 | 1 | 1 | 1 | identical |
| *rps13* | 1 | 1 | 1 | 1 | 1-bp mismatch |
| *rps19* | 2(***rps19-p***) | 1(***rps19-p***) | 1 | 0 | # |
| *rps2* | 1 | 1 | 1 | 2 | 6-bp mismatch |
| *rps3a* | 1 | 1 | 1 | 2 | identical |
| *rps3b* | 1 | 1 | 1 | 1 | 4-bp mismatch |
| *rps4* | 2 | 1 | 3 | 1 | 2-bp mismatch |
| *rps7* | 1 | 1 | 1 | 1 | identical |
| rRNA genes |  |  |  |  |  |
| *rrn18* | 4 | 3 | 2 | 1 | identical |
| *rrn26* | 2 | 2+***rrn26-p*** | 2 | 1 | identical |
|  |  |  |  |  |  |

**Additional File 14.** (continued)

| Gene | Total number of copies  Ks3 Km3 | | Rice | Maize | Difference between  Ks3 and Km3 |
| --- | --- | --- | --- | --- | --- |
| *rrn5* | 4 | 3 | 2 | 1 | identical |
| tRNA genes |  |  |  |  |  |
| *trnA* | 0 | ct | 0 | ct |  |
| *trnC* | ct | ct | ct | ct |  |
| *trnD* | 3-mt^a^ | 2-mt | mt | 3-mt, |  |
| *trnE* | mt | mt | 2-mt | 2-mt |  |
| *trnF* | ct | ct | 2-ct^b^ | ct |  |
| *trnfM* | 5-mt | 3-mt | mt | mt |  |
| *trnH* | 3-ct | 0 | 2-ct | ct |  |
| *trnI* | 2-mt | mt | mt, ct | 2-mt,2-ct |  |
| *trnk* | 3-mt | 3-mt | mt | mt |  |
| *trnM* | 2-mt | mt | mt,2ct | ct |  |
| *trnN* | 3-ct | ct | ct | 2-ct |  |
| *trnP* | 2-mt | 2-mt | 2-mt,ct | 2-mt,ct |  |
| *trnQ* | 3-mt | 3-mt | 2-mt | mt |  |
| *trnS* | 3-mt,ct | 2-mt,ct | 2-mt,2-ct, | 2-mt |  |
| *trnW* | ct | ct | 2-ct | ct |  |
| *trnY* | mt | mt | 2-mt | mt |  |
| *trnV* |  |  | ct | ct |  |
| *trnR* |  |  | ct | mt, ct |  |

# : Significant differences in genes between Ks3 and Km3.

^a^ Mitochondrial origin, figure is the number of copies.

^b^ Chloroplast origin, figure is the number of copies.

^c^ The number of different bases of genes between Ks3 and Km3.

^d^ The Same genes between Ks3 and Km3.

^e^ Boldface: truncated pseudogenes.
