# Supplementary material for: Comparative analysis of mitochondrial genomes between a wheat K-type cytoplasmic male sterility (CMS) line and its maintainer line
Source: BMC Genomics. 2011 Mar 29;12:163. doi: 10.1186/1471-2164-12-163 (PMC3079663; doi:10.1186/1471-2164-12-163)
Supplement: Additional file 16 — Alignments of ATP6, NAD6, and NAD9 between Km3 and Ks3. the file contains the alignments of (A) KMATP6 and KSATP6, (B) KMNAD6 and KSNAD6, and (C) KMNAD9 and KSNAD9. The identical sequences are highlighted with a dark background. [file 1471-2164-12-163-S16.PDF]

A

```
                  *         20         *         40         *         60         *         80         *        100         *        120
KMATP6 : ---MRFLSTDMKDRNMIFAAIT-TNQPIRS--KCSRLPDLHDFFPTN----ISQNFAITPN------LDITPTPER-IAGVTIVLQ----IEEYLGCNESEQGAVNLARTVLGARHRNGE--TWQG-- : 103
KSATP6 : MNKFDLITQTVQSVSHVHSFIFNFSQPALDPQLVNRLLEGVEAPTSKDMDLLLKMYGIAPGNTQALLIEITKMVESYMQQAKLVLPPAYDVTWFCNQFVGVDSFSALKDTFVDLHFKGRESYTWNTGF : 128
                            I    QP        RL                        I P       L IT   E        VL          Q         L  T         E  TW

                   *       140         *       160         *       180         *       200         *       220         *       240         *
KMATP6 : ---ILEDIRAGGGMDNFIQNLP-GAYPETPLDQFAIIPIIDLHVGNFYLSFTNEVLYMLLTVVLVVFLFFVVTKKGGGKSVPNAWQSLVELIYDFVLNLVNEQIGGLSGNVKQKFFPRISVTFTFSLF : 227
KSATP6 : VNFYHEIWRAEDSPPGSAVSAENQLPLENPLDQFAIYPIIDLHVGNFYFTFTNAVLYMLLTVVLVVFLFFVVTKKGGGKSVPNAWQSLVELIYDFVLNLVNEQIGGLSGNVKQKFFPRISVTFTFSLF : 256
               E  RA           P     E PLDQFAI PIIDLHVGNFY  FTN VLYMLLTVVLVVFLFFVVTKKGGGKSVPNAWQSLVELIYDFVLNLVNEQIGGLSGNVKQKFFPRISVTFTFSLF

                  260         *       280         *       300         *       320         *       340         *       360         *       380
KMATP6 : RNPQGMIPFSFTVTSHFLITLALSFSIFIGITIVGFQRHGLHFFSFLLPAGVPLPLAPFLVLLELISYCFRALSLGIRLFANMMAGHSLVKILSGFAWTMLFLNNIFYFIGDLGPLFIVLALTGLELG : 355
KSATP6 : RNPQGMIPFSFTVTSHFLITLALSFSIFIGITIVGFQRHGLHFFSFLLPAGVPLPLAPFLVLLELISYCFRALSLGIRLFANMMAGHSLVKILSGFAWTMLFLNNIFYFIGDLGPLFIVLALTGLELG : 384
         RNPQGMIPFSFTVTSHFLITLALSFSIFIGITIVGFQRHGLHFFSFLLPAGVPLPLAPFLVLLELISYCFRALSLGIRLFANMMAGHSLVKILSGFAWTMLFLNNIFYFIGDLGPLFIVLALTGLELG

                       *       400         *
KMATP6 : VAISQAHVSTISICIYLNDATNLHQNESFHN- : 386
KSATP6 : VAISQAHVSTISICIYLNDATNLHQNESFHN- : 415
         VAISQAHVSTISICIYLNDATNLHQNESFHN
```

B

```
                  *         20         *         40         *         60         *         80         *        100         *        120
KMNAD6 : MRLLAPAFKFHFKGGRRTMILSVLSSPALVSGLMVVRAKNPVHSVLFPILVFCDTSGLLILLGLDFSAMISPVVHIGAIAVSFLFVVMMFNIQIAEIHEEVLRYLPVSGIIGLIFWWEMFFILDNETI : 128
KSNAD6 : MRLLAPAFKFHFKGGRRTMILSVLSSPALVSGLMVVRAKNPVHSVLFPILVFCDTSGLLILLGLDFSAMISPVVHIGAIAVSFLFVVMMFNIQIAEIHEEVLRYLPVSGIIGLIFWWEMFFILDNETI : 128
         MRLLAPAFKFHFKGGRRTMILSVLSSPALVSGLMVVRAKNPVHSVLFPILVFCDTSGLLILLGLDFSAMISPVVHIGAIAVSFLFVVMMFNIQIAEIHEEVLRYLPVSGIIGLIFWWEMFFILDNETI

                   *       140         *       160         *       180         *       200         *       220         *       240         *
KMNAD6 : PLLPTHRNTTSLRYTVYAGKVRSWTNLETLGNLLYTYYSVWFLVSSLILLVAMIGAIVLTMHRTTKVKRQDVFRRNALDSRSHIMNRTISPFGHSHRRSFSSGAGG--PPDNYKETEKMWI------- : 247
KSNAD6 : PLLPTHRNTTSLRYTVYAGKVRSWTNLETLGNLLYTYYSVWFLVSSLILLVAMIGAIVLTMHRTTKVKRQDVFRRNALDSRRNIMNRTISPFGHSHRRSFSSKAEGGESQDSYDPAYISFTRARLGFF : 256
         PLLPTHRNTTSLRYTVYAGKVRSWTNLETLGNLLYTYYSVWFLVSSLILLVAMIGAIVLTMHRTTKVKRQDVFRRNALDSR  IMNRTISPFGHSHRRSFSS A G    D Y

                  260         *       280         *       300
KMNAD6 : ---------------------------------------------------- :   -
KSNAD6 : PGLVPNLDKLLSVLKPEEILFLAFRFPRDANMFKLPPKEDQRYDSRSNG- : 305
```

C

```
                           *         20         *         40         *         60         *         80         *        100         *        120
KMNAD9 : MLCIILFPERWFSGFGIVTKHPGFYTRFNTRACSRSWIHNSKKCVCSFGSLLVASLSLLPLHSHAFLGRTNPTGDFRQVFLLRARSGTKIKLSLFSFMDNQSIFQYSWEILPKKWVHKMKRSEHGNRS : 128
KSNAD9 : ------------------------------------------------------------------------------------------------------MDNQSIFQYSWEILPKKWVHKMKRSEHGNRS :  31
                                                                                                               MDNQSIFQYSWEILPKKWVHKMKRSEHGNRS

                            *       140         *       160         *       180         *       200         *       220         *       240         *
KMNAD9 : YTNTDYPFPLLCFLKWHTYTRVQVSIDICGVDHPSRKRRFEVVHNLLSTRYNSRIRVQTSADEVTRISPVVSLFPSAGRWEREVWDMSGVSSINHPDLRRISTDYGFEGHPLRKDFPLSGYVEVRYDD : 256
KSNAD9 : YTNTDYPFPLLCFLKWHTYTRVQVSIDICGVDHPSRKRRFEVVHNLLSTRYNSRIRVQTSADEVTRISPVVSLFPSAGRWEREVWDMSGVSSINHPDLRRISTDYGFEGHPLRKDFPLSGYVEVRYDD : 159
          YTNTDYPFPLLCFLKWHTYTRVQVSIDICGVDHPSRKRRFEVVHNLLSTRYNSRIRVQTSADEVTRISPVVSLFPSAGRWEREVWDMSGVSSINHPDLRRISTDYGFEGHPLRKDFPLSGYVEVRYDD

                           260         *       280
KMNAD9 : PEKRVVSEPIEMTQEFRYFDFASPWEQRSDG- : 287
KSNAD9 : PEKRVVSEPIEMTQEFRYFDFASPWEQRSDG- : 190
         PEKRVVSEPIEMTQEFRYFDFASPWEQRSDG
```
